# Redox-mediated domino electrosynthesis of *N,N*-dimethylformamide with industrial-relevant productivity and modularized cathodic integration

**DOI:** 10.1038/s41467-026-71637-z

**Published:** 2026-04-17

**Authors:** Yingchun He, Qing Li, Shao Zhang, Bo Zhang, Dong-Dong Ma, Xin-Tao Wu, Qi-Long Zhu

**Affiliations:** 1 State Key Laboratory of Structural Chemistry, Fujian Institute of Research on the Structure of Matter, Chinese Academy of Sciences, Fuzhou, China; 2 School of Materials Science and Engineering, Zhejiang Sci-Tech University, Hangzhou, China; 3 University of Chinese Academy of Science, Beijing, China; 4 Hubei Key Laboratory of Low Dimensional Optoelectronic Materials and Devices, Hubei University of Arts and Science, Xiangyang, China

**Keywords:** Electrocatalysis, Heterogeneous catalysis, Electrocatalysis

## Abstract

Electrosynthesis of *N*,*N*-dimethylformamide (DMF) combines sustainability with economic viability. However, its implementation is hindered by challenges in electrode engineering and reaction control. Herein, a redox-mediated domino electrosynthesis strategy is explored for efficient C–N coupling production of DMF over a binder-free single-molecule-integrated electrode engineered with designed cobalt phthalocyanines. This integrated electrode, featuring enhanced site accessibility and charge transfer capacity, exhibits high and selective methanol oxidation activity, initiating the domino process synergistically facilitated by in situ electrogenerated iodine as a redox mediator. The system operates stably at 50 mA cm^–2^ for over 150 h, achieving >90% Faradaic efficiency and a consistent DMF productivity of 839 μmol h^–1^ cm^–2^. In a flow cell, pairing anodic DMF production with cathodic CO_2_-to-amide conversion yields industrial-scale DMF productivity up to 2.75 mmol h^–1^ cm^–2^ and gram-level amide-containing generic drug production at –200 mA cm^–2^. Furthermore, comprehensive spectroscopic analysis and theoretical calculations elucidate a domino reaction sequence involving several key intermediates, namely *CH_2_O, iododiamine and iminium cation for DMF generation.

## Abstract (web summary)

Converting basic chemicals into high-value products via electrosynthesis combines sustainability with economic benefits. Here, the authors report a redox-mediated domino electrosynthesis of *N*,*N*-dimethylformamide with industrial-relevant productivity and modularized cathodic integration.

## Introduction

**Fig. 1:**
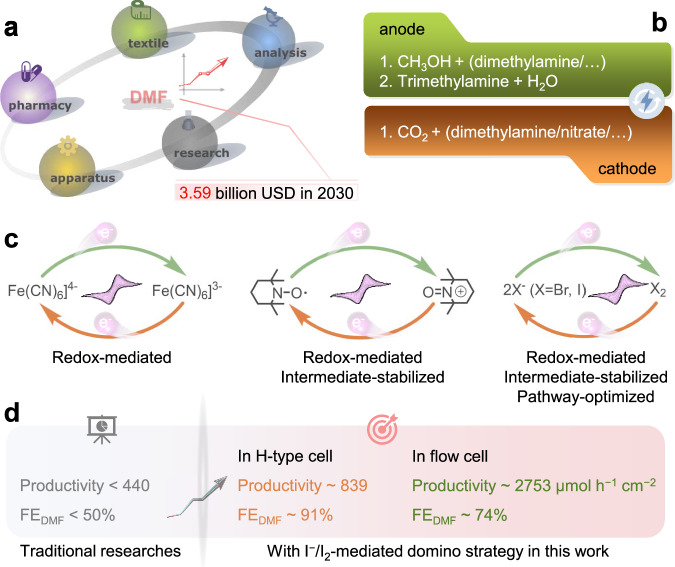
Methods and advances for DMF electrosynthesis. **a** Applications and market size of DMF; **b** reported methodologies for DMF electrosynthesis; **c** illustration of redox mediators; **d** DMF electrosynthesis with I^−^/I_2_-mediated domino strategy in this work.

*N,N*-dimethylformamide (DMF) has become an indispensable solvent and reactive precursor in industrial applications spanning pharmaceuticals, textiles, and advanced manufacturing (Fig. 1a), owing to its beneficial physicochemical properties, including high boiling point, strong polarity, and broad-spectrum solvation capacity^1–3^. The global market for DMF is projected to reach 3.59 billion USD by 2030^4^. However, conventional industrial DMF production relies on thermochemical processes conducted under harsh conditions with elevated temperature and pressure, which are inherently associated with energy-intensive operation protocols and significant environmental burdens (Supplementary Fig. 1)^5^. In contrast, electrochemical DMF synthesis has garnered substantial interest as a sustainable alternative, offering multiple advantages such as ambient reaction conditions, reduced carbon footprint, and precise process control.

Recent advancements in the electrochemical synthesis of DMF have demonstrated promising progress across both anodic and cathodic strategies (Fig. 1b). Within anodic methodologies, Jin et al. achieved a Faradaic efficiency for DMF (FE_DMF_) of 40% via direct electrolysis of trimethylamine using graphite flake as the catalyst^6^. Subsequent work by Li et al. elevated the FE_DMF_ to 50% by employing methanol and dimethylamine (DMA) as feedstocks in conjunction with WO_2_-NiOOH/Ni as the catalyst^7^. On the cathodic side, Fan et al. reported a FE_DMF_ of 37.5% through co-reduction of CO_2_ and DMA^8^, whereas Yan et al. attained a modest FE_DMF_ of 0.32% using NO_3_^–^ and CO_2_ as the nitrogen and carbon sources^9^. Compared to the cathodic DMF electrosynthesis, the anodic tactics offer higher Faradaic efficiencies and other advantages‌. In particular, the electrosynthesis of DMF from “liquid sunlight” methanol is a sustainable option^10^. Nevertheless, critical challenges persist, including suboptimal FE_DMF_ values and inadequate productivity and long-term operational stability across both electrolytic routes. Consequently, achieving substantial enhancements in both activity and selectivity remains imperative for the industrial-scale deployment of electrochemical DMF production.

Notably, our current studies indicate that *N,N,N’,N’-*tetramethylmethanediamine (TMDM), formed spontaneously from DMA and methanol-evolved *CH_2_O species, serves as a pivotal intermediate in the electrochemical synthesis of DMF (Supplementary Fig. 2)^11,12^. Thereby, achieving high DMF production efficiency ‌depends on both‌ the selective methanol oxidation for the generation of TMDM and its rapid conversion to DMF under electro-oxidative conditions. However, it is a challenge that the high-activity catalysts generally tend to over-oxidize methanol to formate, while the low-activity catalysts cannot efficiently promote TMDM electrolysis to generate DMF. Fortunately, these challenges can be effectively addressed by introducing redox mediators (e.g., iodine (I_2_)) to optimize reaction pathways‌ and enhance process efficiency (Fig. 1c)^13–17^. For instance, Wang et al. employed I_2_ as a catalytic mediator to promote the conversion of sulfides/disulfides into electrophilic sulfur species, thereby enabling direct asymmetric α-thiolation of aldehydes^18^. Compared with the traditional methods, in situ electrochemical generation of I_2_ from cost-effective iodide salts (e.g., KI) at the anode has emerged as a promising strategy for electro-thermo-coupled synthesis^19^. This approach not only circumvents the drawbacks of bulk I_2_ usage but also enhances atomic economy and process sustainability.

In addition to the introduction of redox mediators, the rationally designed electrocatalysts can significantly enhance both the selectivity and efficiency of the electrocatalytic process. Traditional powder-based catalyst systems, typically formulated as binder-incorporated inks (e.g., Nafion-based composites) and loaded on conductive substrates, present inherent limitations including inhomogeneous catalyst distribution, active site occlusion, and scalability constraints^20,21^. Notably, our group has recently reported a class of single-molecule-integrated electrodes that utilize strong interfacial interactions between metal phthalocyanine and graphitic carbons to achieve rapid and large-scale preparation of the self-supported catalysts^22,23^. These electrodes exhibit a high degree of active site exposure and good malleability, and can be tailored for specific electrocatalytic reactions through precise control of their sizes, compositions, microenvironments, and nanostructures. By circumventing the limitations of conventional powder-based catalyst systems, this design is expected to provide a versatile platform to optimize the electrochemical conversion kinetics, offering opportunities to advance the industrial viability of electrochemical DMF synthesis.

Based on the aforementioned considerations, we proposed a redox-mediated domino electrosynthesis strategy for high-efficiency C‒N coupling production of DMF through the use of a pyrrolidone-functionalized cobalt phthalocyanine-based single-molecule-integrated electrode (PyCoPc/GF), achieving industrial-relevant productivity with high Faradaic efficiency. The 3D porous architecture of the integrated electrode with PyCoPc molecules implanted onto graphite felt (GF) at the single-molecule level significantly improves the active site accessibility while reducing both mass and charge transfer resistances. During the electrolysis, the selective electro-oxidation of methanol triggers the domino reaction sequence, where the in situ generated I_2_ optimizes the reaction pathway, thereby significantly accelerating the DMF formation. As a result, PyCoPc/GF achieves the high electrocatalytic performance for consistent DMF production over 150 h at 50 mA cm^–2^, retaining a stable FE_DMF_ of >90% and an average productivity of 839 μmol h^–1^ cm^–2^, which is competitive with reported literature (Fig. 1d and Supplementary Table 1). To further validate its industrial application prospects, we developed an electrolysis-paired tandem system with PyCoPc/GF as a bifunctional catalyst to integrate anodic DMF production with cathodic CO_2_-to-amides conversion. A long-term electrolysis experiment, operating at a high current density of 200 mA cm^–2^ in a flow cell, achieved a DMF productivity of 2.75 mmol h^–1^ cm^–2^ at the anode, while simultaneously producing the amide-containing generic drug (**A5**) with a 86% yield via the CO-relayed aminocarbonylation at the cathode. Furthermore, in situ attenuated total reflection-infrared (ATR-IR) spectroscopy, coupled with controlled experiments and density functional theory (DFT) calculations, provides compelling evidence with captured key intermediates for the domino reaction mechanism.

## Results

### Synthesis and characterization

**Fig. 2:**
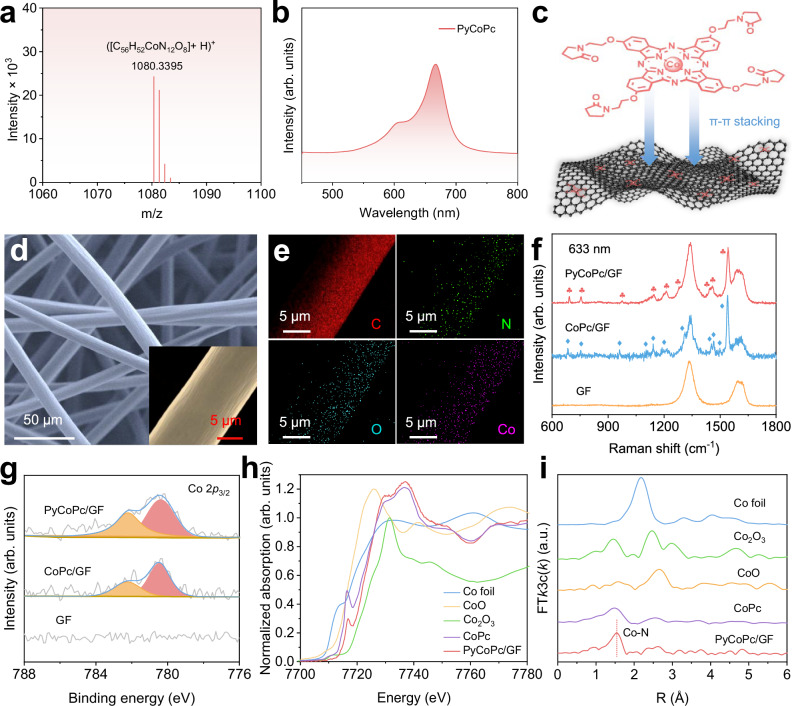
Catalytic material characterization. **a** ESI-MS and **b** UV-Vis spectra of PyCoPc; **c** schematic illustration of construction of single-molecule-integrated catalytic electrode PyCoPc/GF; **d** SEM and **e** EDX elemental mapping images of PyCoPc/GF; **f** Raman, **g** Co 2*p* XPS, **h** Co *K*-edge XANES, and **i** FT-EXAFS spectra of PyCoPc/GF.

It has been demonstrated that the appropriate molecular functionalization of metallophthalocyanines can achieve their single-molecular dispersion onto carbon materials, which fully exposes the active sites and enhances their utilization efficiency, thereby benefiting subsequent electrocatalytic reactions^24^. Based on this rationale, a distinctive cobalt phthalocyanine decorated with peripheral pyrrolidone groups (PyCoPc) was synthesized and characterized (Fig. 2a, b and Supplementary Figs. 3, 4). The electrospray ionization mass spectrometry (ESI-MS) analysis displays a dominant peak at 1080.3393, confirming the successful synthesis of PyCoPc. Furthermore, the UV-Vis spectrum (Fig. 2b) reveals a distinct Q-band characteristic peak of phthalocyanine at 672 nm, suggesting the tendency toward single-molecular dispersion of PyCoPc in this solvent system^25^. Compared to commercial cobalt phthalocyanine (CoPc), PyCoPc demonstrates much better solubility without intermolecular aggregation (Supplementary Fig. 5).

GF, a graphitic carbon material synthesized at high temperatures (2000 ~ 3000 °C), demonstrates a hierarchical 3D network architecture coupled with high electronic conductivity, establishing it as an ideal substrate for catalytic applications (Supplementary Fig. 6). As illustrated in Fig. 2c, GF was utilized as a carbon support to disperse the PyCoPc molecules via a solution-based process. Leveraging the strong interfacial π–π heterostacking interactions, the highly-soluble PyCoPc molecules can be anchored onto GF fibers in a single-molecular dispersion mode, forming the integrated catalytic electrode (PyCoPc/GF) with a 3D porous network structure. This architecture ensures high exposure of active sites while maintaining structural integrity, offering a robust platform for efficient electrocatalytic applications.

Scanning electron microscopy (SEM) was conducted to observe the surface structure of the single-molecule-integrated electrode. As shown in Fig. 2d and Supplementary Fig. 7, the surface of PyCoPc/GF reveals a smooth fiber morphology devoid of discernible particulate aggregates. The energy-dispersive X-ray (EDX) elemental mapping images reveal the homogeneous distributions of Co and N elements across the fibers (Fig. 2e), indicating uniform anchoring of PyCoPc molecules‌. To better characterize the nanostructure of PyCoPc/GF, the integrated electrode was ball-milled (Supplementary Fig. 8) prior to transmission electron microscopy (TEM) and spherical aberration-corrected high-angle annular dark-field scanning transmission electron microscopy (AC-HAADF-STEM) characterizations. TEM and AC-HAADF-STEM analyses further confirm the single-molecular dispersion of PyCoPc on the GF surface (Supplementary Figs. 9 and 10a), attributable to enhanced anchoring facilitated by the pyrrolidone group substitution. In contrast, the lower solubility of CoPc resulted in partial single-molecular dispersion with inevitable aggregation (Supplementary Fig. 10b). The Co content of PyCoPc/GF (0.0046%) is higher than that in CoPc/GF (0.0033%) (Supplementary Table 2). Furthermore, the X-ray diffraction (XRD) patterns of single-molecule-integrated electrodes display only characteristic peaks of graphitic carbon (Supplementary Fig. 11), unlike those of PyCoPc (Supplementary Fig. 12), providing additional evidence against molecule aggregation‌. The CoPc/GF counterpart prepared with commercial CoPc displays a similar surface structure (Supplementary Fig. 13). Confocal Raman spectroscopy confirms the presence of characteristic phthalocyanine peaks in both PyCoPc/GF and CoPc/GF (Fig. 2f and Supplementary Fig. 14). Besides, the contact angle measurements demonstrate that the introduction of PyCoPc optimizes the surface wettability of the electrode, probably promoting substrate enrichment for electrocatalysis at the electrode interface (Supplementary Fig. 15)‌. X-ray photoelectron spectroscopy (XPS) and X-ray absorption spectroscopy (XAS) analyses were further employed to investigate the electrode surface chemical states and coordination structure. As shown in Fig. 2g, the high-resolution Co 2*p*_3/2_ XPS spectrum exhibits a characteristic peak of Co^2+^ at 780.4 eV, corresponding to the Co^2+^ species in phthalocyanine. The X-ray adsorption near edge structure (XANES) spectra reveal a distinct pre-edge absorption at 7716.9 eV in PyCoPc/GF, corresponding to the dipole-forbidden 1 *s* to 4*p* transition characteristic of Co-N_4_ moieties with a *D*_4h_ symmetry (Fig. 2h)^26,27^. Compared to commercial CoPc, the Co-N_4_ moieties in PyCoPc/GF exhibit a 0.4 eV blue shift from 7716.5 to 7716.9 eV due to the synergistic interaction of peripheral pyrrolidone groups and the GF substrate. Furthermore, the local structure of Co coordination was further scrutinized by Fourier-transformed extended X-ray absorption fine structure (FT-EXAFS) analysis (Fig. 2i), demonstrating a primary coordination shell with Co-N scattering paths at ~1.54 Å^28,29^. The aforementioned characterizations collectively demonstrate that the single-molecule-integrated electrode modified with PyCoPc and featuring a three-dimensional network-like porous structure has been successfully fabricated.

### Electrocatalytic study for DMF electrosynthesis

**Fig. 3:**
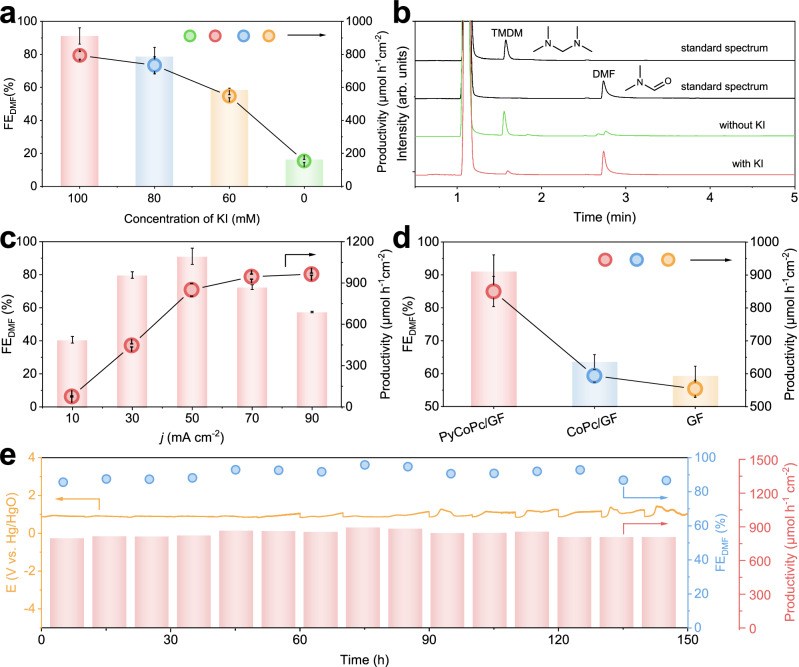
Activity evaluation for DMF electrosynthesis. Electrocatalytic performance for DMF electrosynthesis in 0.7 M K_2_CO_3_ in an H-type electrolytic cell: **a** FE_DMF_ and productivity of PyCoPc/GF at 50 mA cm^–2^ with different concentrations of KI; **b** GC spectra of products electrolyzed with or without 100 mM KI at 50 mA cm^–2^; **c** FE_DMF_ and productivity of PyCoPc/GF at different current densities with 100 mM KI; **d** FE_DMF_ and productivity of PyCoPc/GF, CoPc/GF and GF at 50 mA cm^–2^ with 100 mM KI; **e** stability test for PyCoPc/GF at 50 mA cm^–2^.

The single-molecule-integrated electrodes were directly employed as working electrodes for subsequent tests. Initially, the catalytic activity toward DMF electrosynthesis was systematically evaluated in an H-type electrolytic cell (Supplementary Fig. 16). The products were quantitatively analyzed using gas chromatography (GC) and nuclear magnetic resonance (NMR) spectroscopy (Supplementary Fig. 17). Prior to testing, the PyCoPc/GF electrode was subjected to cyclic voltammetry (CV) activation within a potential window of 0-1 V (vs. Hg/HgO) to ensure electrochemical stability. The structural integrity of the PyCoPc moiety on the GF substrate was verified through Raman spectroscopic analysis (Supplementary Fig. 18). Linear sweep voltammetry (LSV) was initially employed to evaluate the effect of KI on the catalytic reaction. As shown in Supplementary Fig. 19, the addition of KI significantly increased the current and induced a negative shift in the onset potential, from approximately 0.42 V without KI to about 0.30 V with 100 mM KI. It was observed that the introduction of KI in the electrolyte leads to significant improvements in both productivity and FE_DMF_, with the FE_DMF_ increasing dramatically from 16% in the absence of KI to 91% at an optimal concentration of 100 mM KI (Fig. 3a). Further analyses of the post-electrolysis electrolyte by GC, gas chromatography-mass spectrometry (GC-MS), and NMR confirm that in situ electrogenerated I_2_ facilitates highly selective electrosynthesis of DMF (Fig. 3b and Supplementary Figs. 20–22).

Additionally, the choice of membrane critically affects ion transport dynamics and operational voltage, consequently influencing the catalytic performance metrics^30^. As shown in Supplementary Fig. 23, all three membranes exhibited comparable catalytic activity, with the bipolar membrane (BM) achieving a marginally higher FE_DMF_ (91%). The BM was selected for subsequent testing due to its dual functionality of providing additional OH^–^ to the anode through electrochemical water dissociation at the interfacial layer, thereby promoting DMF electrosynthesis, while effectively suppressing organic crossover from the anolyte to the catholyte during prolonged operation to prevent interference with electrode reactions in both compartments^31,32^. Under 100 mM KI concentration and BM conditions, the effect of current density on DMF production was investigated. As illustrated in Fig. 3c, FE_DMF_ increases with current density, reaching a maximum of 91% with the high productivity of 850 μmol h^–1^ cm^–2^ at 50 mA cm^–2^. Beyond this threshold, FE_DMF_ gradually declines, possibly due to an increase in side reactions (Supplementary Fig. 24). A comparative study of the PyCoPc/GF, CoPc/GF, and GF electrodes under optimal conditions reveals distinct performance differences. PyCoPc/GF exhibits a significantly higher current density than both CoPc/GF and GF, and a much lower onset potential of 0.30 V compared to CoPc/GF (0.44 V) and GF (0.53 V), demonstrating its good catalytic activity (Supplementary Fig. 25). As shown in Fig. 3d, PyCoPc/GF achieves high FE_DMF_ (91%) and productivity (850 μmol h^–1^ cm^–2^), significantly surpassing CoPc/GF (64%, 594 μmol h^–1^ cm^–2^) and GF (59%, 554 μmol h^–1^ cm^–2^) at 50 mA cm^–2^. This significant performance enhancement can be attributed to the single-molecular dispersion of PyCoPc onto the GF, which maximizes active site accessibility, as evidenced by electrochemical active surface area measurements (Supplementary Fig. 26). Besides, an optimized local microenvironment facilitates the electron and mass transfer and the enrichment of substrates/reaction intermediates, thereby promoting DMF formation. The turnover frequency (TOF) measurements further corroborated the enhanced intrinsic activity. As shown in Supplementary Fig. 27, the calculated TOF of PyCoPc/GF for DMF generation is 82.54 s^–1^, higher than that of CoPc/GF (67.52 s^–1^). Furthermore, the long-term stability test for 150 h demonstrates that PyCoPc/GF exhibits consistent catalytic activity, capable of maintaining the high FE_DMF_ (~90%), DMF productivity (839 μmol h^–1^ cm^–2^) and a DMA conversion rate of ~35% (Fig. 3e and Supplementary Figs. 28–33). Critically‌, the quantification of active iodine species after reaction revealed 92 mM residual concentration (Supplementary Fig. 34), representing 92% retention compared to the initial KI concentration (100 mM), confirming mediator reversibility and system stability. To further demonstrate the versatility of the I_2_-mediated strategy, the substrate scope was expanded to additional amines, affording *N,N-*diethylformamide and *N*-formylmorpholine with productivities of 383 and 145 μmol h^–1^ cm^–2^, respectively (Supplementary Fig. 35).

### Mechanistic study

**Fig. 4:**
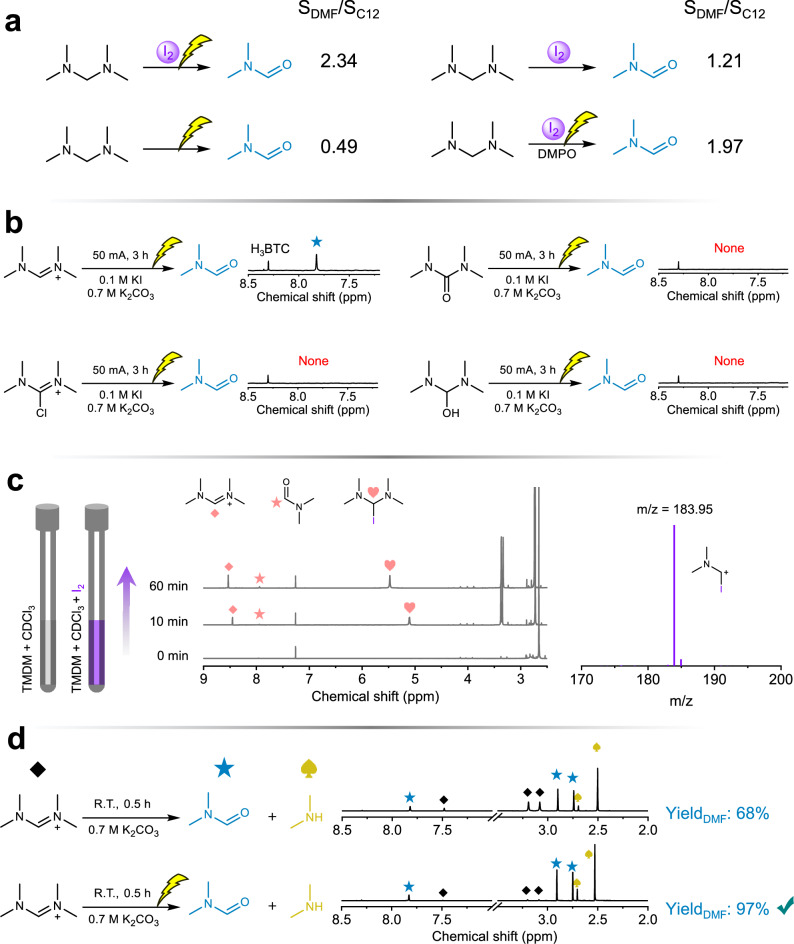
Intermediate identification. Reaction pathway analysis:‌ **a** control experiments demonstrating iodide-facilitated DMF synthesis (C12: dodecane internal standard; data presented as chromatographic peak area ratios); **b** identification of key reaction intermediates through NMR monitoring of DMF signature signals; **c** formation and verification of iododiamine and iminium cation intermediates by quasi-in situ NMR and mass spectrometry; **d** yield analysis of iminium cation intermediate conversion to DMF under varied conditions. Yellow lightning denotes electricity.

To gain a deep comprehension of the I_2_-mediated electrosynthesis of DMF, a series of in situ and quasi-in situ spectroscopy and controlled experiments were conducted. Initially, the in situ ATR-IR spectroscopy was employed to monitor the evolution of the intermediates during the reaction at 0.5 V vs. Hg/HgO. As shown in Supplementary Fig. 36, the peak at 1662 cm^–1^ corresponds to *CH_2_O derived from methanol partial oxidation, which subsequently reacts with DMA in 0.7 M K_2_CO_3_ to form TMDM rapidly. Compared to the KI-free system, KI addition significantly enhances *CH_2_O intermediate generation and facilitates rapid DMF production via I_2_-mediated electro-oxidative decomposition of TMDM, as evidenced by the characteristic DMF peak at 1623 cm^–1^ ^6,8^. Furthermore, experiments with ^13^C-labeled K_2_CO_3_ confirmed that CO_3_^2–^ in the electrolyte was not involved in the reaction (Supplementary Fig. 37). To further verify the promotional effect of I_2_ on the electrochemical synthesis of DMF, controlled experiments using TMDM as a substrate were conducted. As illustrated in Fig. 4a, when the electrolysis was performed with the addition of I_2_, the highest DMF content was achieved and the ratio of the integrated chromatographic peak area of DMF to that of the internal standard dodecane (S_DMF_/S_C12_) reached 2.34, indicating that the synergistic effect of electrochemically generated I_2_ and electricity significantly enhances the electrosynthesis performance of DMF. In contrast, direct electrolysis of TMDM without I_2_ yielded an S_DMF_/S_C12_ ratio of 0.49, which is attributed to the difficulty of the direct electro-oxidative dehydrogenation of TMDM^12^. Additionally, when the 5,5-dimethyl-1-pyrroline N-oxide (DMPO) spin trap was added, the S_DMF_/S_C12_ ratio was minimally decreased to 1.97, suggesting that the process of TMDM converting to DMF is not primarily driven by radical reactions. In addition, we electrolyzed several possible key reaction intermediates, such as tetramethylurea, bis(dimethylamino)methanol, tetramethyl-chloroformamidinium chloride, identifying (dimethylaminomethylene)dimethylammonium cation ((CH_3_)_2_NCH = N^+^(CH_3_)_2_) as the key intermediate (Fig. 4b and Supplementary Figs. 38–41). Notably, the quais-in situ NMR and GC-MS reveal the accumulation of the iodinated intermediate (1-iodo-*N,N,N’,N’*-terametheylmethanediamine) and iminium cation, confirming the involvement of the I_2_-promoted mechanism in the DMF electrosynthesis (Fig. 4c; Supplementary Figs. 42 and 43). The chemical shift values in the ^1^H NMR spectrum of the product may vary due to changes in the NMR environment‌^33^. Additionally, comparative experiment results conclusively demonstrate that the synergistic effect between alkaline electrolyte and electricity stimulation significantly accelerates the conversion of iminium cation to DMF (Fig. 4d and Supplementary Fig. 44). Recent literature suggests that amide formation may proceed via intermediates such as methylisocyanide or nitrile species^34,35^. However, our in situ ATR-IR spectroscopy analysis detected no significant signals in the 2100–2200 cm^–1^ region (Supplementary Fig. 45). This absence may arise from the significant influence of factors such as electrolyte composition and catalyst type on the reaction mechanism.

**Fig. 5:**
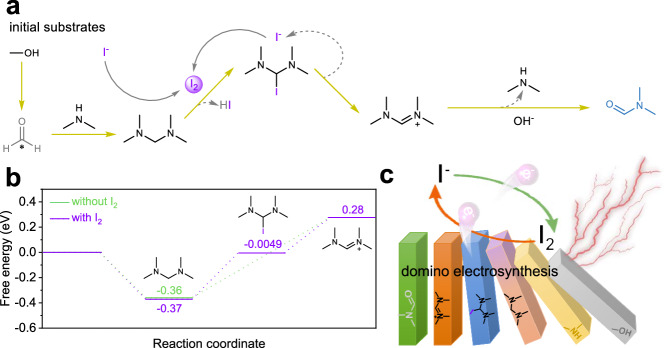
Mechanism analysis. **a** Proposed reaction mechanism scheme; **b** Gibbs free energy for the formation of iminium cation over PyCoPc/GF with or without I_2_; **c** schematic illustration of the domino electrosynthesis process for DMF. Dominoes represent different reaction substrates and lightning denotes electricity in (**c**).

Based on the aforementioned mechanism validation experiments, we propose a comprehensive reaction mechanism for the I_2_-mediated domino electrooxidation synthesis of DMF using methanol and DMA as raw materials (Fig. 5a). This domino reaction sequence commences with the electrochemical oxidation of methanol to *CH_2_O under alkaline conditions, followed by the rapid nucleophilic addition with DMA to readily form TMDM. Significantly, the generated TMDM is efficiently converted to the crucial (dimethylaminomethylene)dimethylammonium cation with the assistance of the redox mediator, i.e., iodine, under electrolysis, which is subsequently oxidized to DMF. It should be noted that I_2_ and *CH_2_O species may generate simultaneously. DFT calculations further confirm the crucial role of I_2_ in facilitating the key iminium cation formation. However, it is important to note that these calculations are based on simplified model systems under idealized conditions, which may not fully capture the complexity of the dynamic catalyst–electrolyte interface under operating electrochemical potentials. As illustrated in Fig. 5b and Supplementary Fig. 46, the presence of I_2_ serves a key role by dividing the original large free energy barrier (0.64 eV) between TMDM and iminium cation into two thermodynamically favorable steps, thus greatly promoting the DMF production. Specifically, the free energy difference from TMDM to the iododiamine intermediate is reduced to 0.37 eV, while the subsequent conversion to the iminium cation requires only 0.28 eV. This in situ-generated iodine-mediated pathway significantly accelerates the formation of the cationic intermediate, thereby enhancing the overall efficiency of the domino electrosynthesis toward DMF production. Figure 5c vividly illustrates the domino electrosynthesis process of DMF in the presence of the redox mediator.

### Electrocatalytic study for electrolysis-paired tandem synthesis

In conventional electrosynthesis, most studies focus solely on one half-reaction while neglecting the other, leading to reduced spatiotemporal and energy efficiencies. The paired electrosynthesis strategy enables the simultaneous generation of high-value-added products at both the cathode and anode, aligning with green principles^36–38^. Furthermore, the products of electrosynthesis can be further upgraded by performing electro-thermal cascade reactions^39–41^. Literature reports indicate that the Ni phthalocyanine-based single-molecular heterojunctions exhibit high electrocatalytic CO_2_ reduction (ECR) performance for CO production^24,42^. Inspired by this, we hypothesize that the PyCoPc/GF catalyst would demonstrate good ECR performance and could be integrated with anodic DMF electrosynthesis to establish a parallel paired electrosynthesis system.

Since the cathodic CO_2_-to-amide conversion comprises the electrocatalytic CO generation via ECR and thermocatalytic aminocarbonylation to amides, we initially evaluated the ECR performance of PyCoPc/GF in an H-type cell (Supplementary Fig. 47). Compared to CoPc/GF and GF, PyCoPc/GF displays a high catalytic activity for ECR, maintaining CO Faradaic efficiency (FE_CO_) above 90% within the potential range of –0.78 to –0.98 V vs. RHE. Notably, it achieves a CO partial current density (*j*_CO_) of 45 mA cm^–2^ at –1.1 V vs. RHE, significantly outperforming CoPc/GF (1.0 mA cm^–2^) and GF (0.17 mA cm^–2^). Furthermore, stability test conducted at –0.88 V vs. RHE over 20 h demonstrates sustained performance with an average FE_CO_ of 90% and consistent CO production rate of 45 μmol h^–1^ cm^–2^, establishing a robust foundation for subsequent paired cascade systems.

**Fig. 6:**
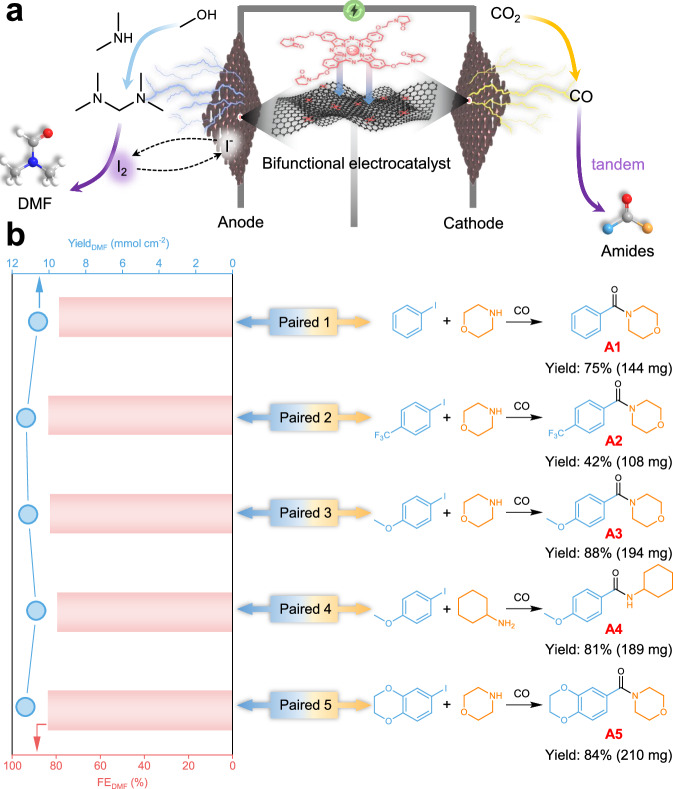
Electrolysis-paired tandem synthesis in the H-type cell. Paired electrosynthesis system integrating anodic DMF electrosynthesis and cathodic CO_2_-to-amide conversion: **a** schematic illustration of electrolysis-paired tandem synthesis; **b** catalytic performance of the electrolysis-paired tandem synthesis for simultaneously producing DMF at the anode and amides (**A1**–**A5**) at the cathode. Lightning in (**a**) denotes electricity.

Leveraging the dual functionality of PyCoPc/GF, a parallel paired electrosynthesis system in a bipolar membrane separated H-type electrolyzer was constructed. This system synergistically combines anodic DMF production with cathodic CO generation. Furthermore, the thermocatalytic cascade reactions were implemented to upgrade the cathodic CO into high-value amides, ultimately achieving the co-production of amides at both electrodes (Fig. 6a). As demonstrated in Fig. 6b and Supplementary Figs. 48–58, five independent electrolysis-paired tandem reactions were performed by varying iodide and amine substrates, producing five high-value amides (**A1**–**A5**). Experimental results demonstrate that all cascade products except **A2** achieved yields above 75%, while the lower yield of **A2** (~65%) is likely due to the electron-withdrawing effect of the fluorine substituent compromising reaction activity. Additionally, each reaction system maintained an average FE_DMF_ of 81%, with DMF yield (Yield_DMF_) consistently exceeding 10.5 mmol cm^–2^. The ^13^CO_2_ isotopic labeling experiment demonstrates that the in situ generated ^13^CO intermediate from ECR was successfully converted into ^13^C-labeled amide products via aminocarbonylation (Supplementary Figs. 59 and 60).

**Fig. 7:**
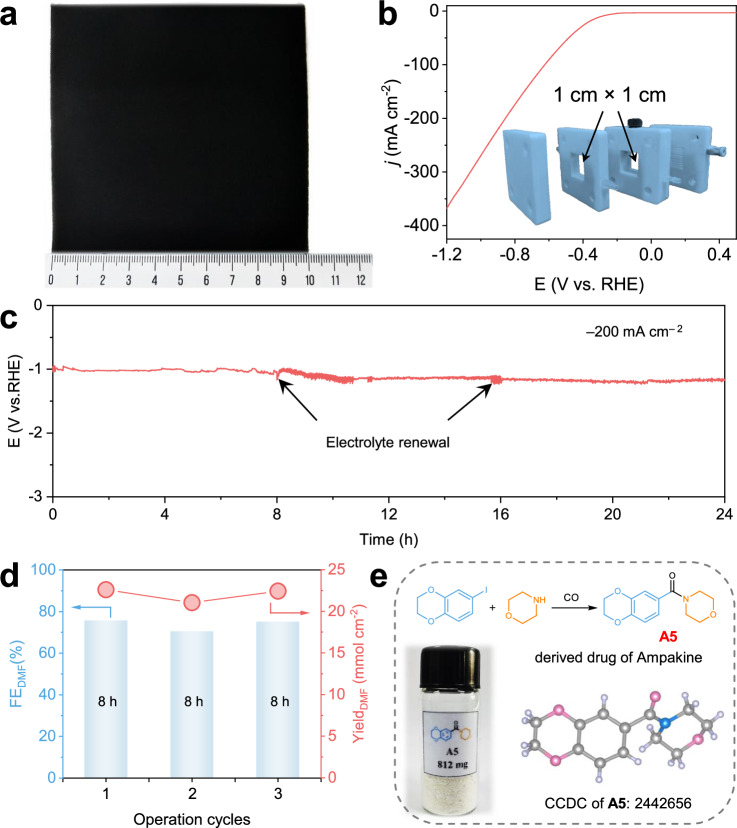
Scale-up electrode fabrication and electrolysis-paired tandem synthesis in the flow cell. Electrocatalytic performance of electrolysis-paired tandem synthesis in a flow cell: **a** large-size preparation of PyCoPc/GF-GDE with dimensions of 2 mm × 100 mm × 100 mm; **b** LSV curve without iR compensation; **c** stability test at –200 mA cm^–2^; **d** FE_DMF_ and Yield_DMF_ at the anode; **e** photographic image and single-crystal structure of the generic drug **A5** obtained at the cathode. The inset in (**b**) is a photo of the flow cell setup.

Compared to the conventional H-type cell, the rationally designed flow cell configuration‌ enables continuous product generation through electrolyte circulation. Furthermore, by constructing the gas diffusion electrode (GDE) with distinctive gas-liquid-solid triple-phase interfaces (Supplementary Fig. 61), this configuration is capable of addressing the critical limitations of traditional H-type cells in ECR, including mass transfer constraints and low CO_2_ solubility, thereby achieving high-efficiency operation under high current densities. Notably, in the paired electrosynthesis, substituting the catholyte from neutral 0.5 M KHCO_3_ to 1 M KOH effectively reduces overall cell voltage while simultaneously improving the performance of both cathodic and anodic reactions^43–45^. In view of this, a GF-based single-molecule-integrated GDE (PyCoPc/GF-GDE) with dimensions of 2 mm × 100 mm × 100 mm was prepared, and its successful construction was confirmed through cross-sectional SEM analysis (Fig. 7a and Supplementary Fig. 62). Additionally, a scalable immersion method was employed to massively produce the PyCoPc/GF electrode with dimensions of 2 mm × 100 mm × 1280 mm (Supplementary Fig. 63). By randomly selecting and trimming electrode segments, we constructed a flow cell for the electrolysis-paired tandem synthesis, employing PyCoPc/GF as the anode and PyCoPc/GF-GDE as the cathode. This configuration demonstrates competitive performance for the paired electrosynthesis, achieving a current density of –370 mA cm^–2^ at –1.2 V vs. RHE, thus validating the effectiveness of our electrode design and system optimization (Fig. 7b).

To further evaluate the industrial applicability of this strategy, the electrolysis-paired tandem synthesis was conducted under industrial-level current conditions (−200 mA cm^–2^) for 24 h, with the electrolyte being replaced every 8 h over three consecutive cycles (Fig. 7c). As shown in Fig. 7d, the anode electrolyte was analyzed after each cycle, revealing that the FE_DMF_ remained above 70% in all three cycles, with an average Yield_DMF_ of 22 mmol cm^–2^. This high DMF productivity reaching up to 2.75 mmol h^–1^ cm^–2^ achieved in such a paired electrosynthesis system demonstrates its promising prospect for industrial scale-up. Technoeconomic analysis (TEA, Supplementary Note 1 and Supplementary Figs. 67–69) preliminarily shows that the daily profit of DMF from this electrosynthesis can reach 688.6 USD t^–1^ day^–1^, highlighting its potential for practical implementation. Simultaneously, the ECR performance at the cathode was monitored, and the average FE_CO_ reached 93% (Supplementary Fig. S64). To valorize the cathodically generated CO, an electro-thermal cascade upgrading process was implemented for the synthesis of **A5** as a representative example. This compound is an analog of AMPA receptor positive allosteric modulators, structurally inspired by reference compounds such as CX546 and CX614, shows promise for research into psychiatric disorders like Alzheimer’s disease and depression^46,47^. By scaling up the reactants, 812 mg of **A5** was isolated with a yield of 86% (Fig. 7e). Subsequent characterization by NMR spectroscopy and single-crystal X-ray diffraction confirmed both the high purity and the molecular configuration of the synthesized **A5** (Supplementary Figs. 65 and 66; Supplementary Tables 3 and 4).

## Discussion

In summary, we have demonstrated a redox-mediated domino electrosynthesis strategy for efficient DMF production using a single-molecule-integrated PyCoPc/GF electrode, achieving a FE_DMF_ of 90% and a productivity of 839 μmol h^–1^ cm^–2^ at 50 mA cm^–2^ for over 150 h. Combined in situ and quasi-in situ spectroscopic investigations and DFT calculations decipher a complete domino reaction mechanism. This domino reaction sequence is triggered by the methanol oxidation, generating the *CH_2_O intermediate, which subsequently condenses with DMA to yield TMDM. This is followed by the electro-oxidative step that produces DMF with the involvement of iododiamine and iminium cation intermediates. Furthermore, through integrating anodic I_2_-mediated domino DMF synthesis and cathodic CO-relayed aminocarbonylation, the electrolysis-paired tandem synthesis of DMF and amides was verified, indicating the good expansibility and compatibility of the system and the electrode materials. Notably, operating under industrially relevant conditions (–200 mA cm^–2^) in a flow cell, the system achieved a high anodic DMF yield of 2.75 mmol h^–1^ cm^–2^, while concurrently producing gram-level of amide-containing generic drug at the cathode, demonstrating its industrial potential. This work not only establishes a robust platform for sustainable DMF production but also provides fundamental insights into the design principles of molecularly integrated electrodes for efficient electrosynthesis applications.

## Methods

### Chemicals and materials

Methanol (CH_3_OH, A.R., ≥99.5%, Sinopharm Chemical Reagent Co. (SCRC), China), aluminum oxide (200-300 mesh, neutral, A.R., Greagent, China), dimethylamine (C_2_H_7_N, A.R., Shanghai Adamas Reagent Co., 40 wt% solution in water, China), dichloromethane (CH_2_Cl_2_, A.R., ≥99.5%, SCRC, China), *N,N,N,N*-tetramethyldiaminomethane (C_5_H_14_N_2_, R.G., 97%, Shanghai Adamas Reagent Co., China), 1,2-dichlorobenzene (C_6_H_4_Cl_2_, A.R., ≥99%, SCRC, China), graphite felt (Tianjin Yufeng Carbon Co., Ltd, China), 1,8-diazabicyclo-(5,4,0)-undec-7-ene (DBU) (C_9_H_16_N_2_, R.G., 18%, Shanghai Adamas Reagent Co., China), cobalt (II) acetate tetrahydrate (C_4_H_14_CoO_8_, A.R., ≥99.5%, SCRC, China), *N,N*-dimethylformamide (C_3_H_7_NO, A.R., ≥99.5%, SCRC, China), dimethyl sulfoxide-D_6_ (C_2_D_6_OS, R.G., (D, 99.9%), TMS (0.03%), Shanghai Adamas Reagent Co., China), chloroform-D (CDCl_3_, R.G., (D, 99.8%), TMS (0.03%), Shanghai Adamas Reagent Co., China), 4-nitrophthalonitrile (C_8_H_3_N_3_O_2_, R.G., 98%, Aladdin Co., China), *N*-(2-hydroxyethyl)−2-pyrrolidone (C_6_H_11_NO_2_, R.G., 98%, Shanghai Adamas Reagent Co., China), potassium carbonate (K_2_CO_3_, A.R., 99%, SCRC, China), dimethyl sulfoxide (C_2_H_6_OS, A.R., ≥99.5%, SCRC, China), cobalt phthalocyanine (C_32_H_16_CoN_8_, R.G., ≥95%, Shanghai Adamas Reagent Co., China), iodine (I_2_, R.G., Shanghai Adamas Reagent Co., China), potassium iodide (KI, RG., Shanghai Adamas Reagent Co., China), iodobenzene (C_6_H_5_I, R.G., 99%, Shanghai Adamas Reagent Co., China), morpholine (C_4_H_9_NO, R.G., 99%, Shanghai Adamas Reagent Co., China), 4-iodobenzotrifluoride (C_7_H_4_F_3_I, R.G., 98%, Shanghai Adamas Reagent Co., China), 4-iodoanisole (C_7_H_7_IO, R.G., 99%, Shanghai Adamas Reagent Co., China), cyclohexylamine (C_6_H_13_N, R.G., 99%, Shanghai Adamas Reagent Co., China), 6-iodo-2,3-dihydrobenzo[B][1,4]dioxine (C_8_H_7_IO_2_, R.G., 99%, Shanghai Adamas Reagent Co., China), palladium (II) acetate (C_4_H_6_O_4_Pd, 98%, Shanghai Haohong Biomedical Technology Co., China), toluene (C_7_H_8_, toluene, A.R., ≥99.5%, SCRC, China), bis[2-(diphenylphosphino)phenyl] ether (DPEphos) (C_36_H_28_OP_2_, R.G., 97%, Shanghai Adamas Reagent Co., China), tetramethylurea (C_5_H_12_N_2_O, R.G., ≥99%, Shanghai Adamas Reagent Co., China), tetramethyl-chloroformamidinium chloride (C_5_H_12_Cl_2_N_2_, R.G., 98%, Shanghai Adamas Reagent Co., China), were used without further purification. The cationic membrane (CM, Nafion-117, DuPont Co.), anionic membrane (AM, Fumasep FAB-PK-130, FuMATech), and bipolar membrane (BM, Fumasep FBM-PK, FuMATech) were treated according to the manufacturer’s standard instructions.

### Characterization

Ultraviolet visible (UV-Vis) spectra were recorded on a Lambda 365 spectrophotometer. Electrospray ionization mass spectra (ESI-MS) were obtained on an Agilent 6550 iFunnel Q-TOF spectrometer. The metal contents of the catalysts were analyzed using ICP-AES on Ultima 2. X-ray photoelectron spectroscopy (XPS) data were obtained from a Thermo Fisher ESCALAB 250Xi spectrometer. X-ray diffraction (XRD) patterns were performed on a MiniFlex diffractometer (MiniFlex600, Rigaku) at 40 kV voltage and 15 mA current with Cu Kα radiation. Scanning electron microscopy (SEM) images were obtained on a Zeiss SIGMA300. Nuclear magnetic resonance (NMR) spectra were recorded on the ECZ600R. The liquid products were detected by Shimadzu GCMS-QP2020 Gas Chromatograph Mass Spectrometer (GC-MS) and Shimadzu GC-2018 gas chromatography (GC) featuring an FID detector and HP-5 column. The gas products were detected by Agilent 7820A GC, which was equipped with a thermal conductivity detector and a flame ionization detector that was used to quantify the gas products (CO and H_2_). In situ attenuated total reflection-infrared (ATR-IR) spectra were recorded on the NICOLET 6700 instrument. Raman spectra and in situ Raman spectra were recorded on LabRAM HR Evolu by using a 532 or 633 nm laser. X-ray absorption spectra were carried out at ID26 beamline of European Synchrotron Radiation Facility (ESRF in France), beam size at 1 mm × 1 mm. To better characterize the catalyst’s nanostructure, integrated electrodes were ball-milled for 24 h using a planetary ball mill (QM-3SP04, ‌Nanjing Nanda Instrument Co., Ltd.). The resultant powder collected from the mill jar was subsequently characterized. Transmission electron microscopy (TEM) images and high-resolution transmission electron microscopy (HRTEM) images were collected from FEI Titan Themis 200 at an acceleration voltage of 200 kV. Spherical aberration-corrected high-resolution annular dark-field scanning transmission electron microscopy (AC-HAADF-STEM) images were collected from JEM-ARM300F.

### Synthesis of 4-(2-pyrrolidinone-*N*-ethyloxy) phthalonitrile (Py-CN_2_)

*N*-(2-hydroxyethyl)−2-pyrrolidone (2.65 g, 20.1 mmol) and anhydrous K_2_CO_3_ (13.82 g, 100.0 mmol) were stirred in dry DMSO (25 mL) at room temperature for 5 h. Subsequently, 4-nitrophthalonitrile (3.46 g, 20.0 mmol) was added and the mixture was continuously stirred for 72 h. The reaction mixture was then filtered to remove the salt, after which the filtrate was poured into 100 mL of cold water. The precipitate was collected and washed with water several times. The precipitate was purified by aluminum oxide (200–300 mesh) column chromatography using CH_2_Cl_2_/MeOH as eluents to give the solid product. ^1^H NMR (600 MHz, DMSO-d_6_), δ/ppm (Supplementary Fig. 3): 8.04–8.05 (d, *J* = 8.4 Hz, 1H), 7.78–7.89 (d, *J* = 3.0 Hz, 1H), 7.45–7.46 (dd, *J* = 3.0 Hz, 1H), 4.25–4.26 (t, *J* = 5.4 Hz, 2H), 3.55–3.57 (t, *J* = 5.4 Hz, 2H), 3.42–3.45 (t, *J* = 6.6 Hz, 2H), 2.19–2.22 (t, *J* = 7.8 Hz, 2H, CH_2_), 1.88–1.93 (m, 2H).

### Synthesis of tetra-(4-(2-pyrrolidinone-*N*-ethyloxy)) cobalt phthalocyanine (PyCoPc)

Py-CN_2_ (511 mg, 2.0 mmol), cobalt (II) acetate tetrahydrate (138 mg, 0.6 mmol) and dried 1-pentanol (5 mL) were added to a 25 mL of two-neck round-bottom flask and stirred at 110 °C for 30 min under N_2_ atmosphere. Then 0.9 mL of DBU was added and stirred at 130 °C for 24 h. After cooling to room temperature, the black-green product was precipitated and washed with n-hexane several times. The crude product was then dissolved in CH_2_Cl_2_ and purified by neutral aluminum oxide column chromatography using CH_2_Cl_2_/MeOH (*v*/*v* = 30:1–20:1) as eluent to give a dark violet solid. HRMS (*m/z*): [M + H]^+^ calcd. for C_56_H_53_CoN_12_O_8_, 1080.3393; found, 1080.3395 (Fig. 2a and Supplementary Fig. 4). UV/Vis: *λ*_max_ 672 nm.

### Pre-treatment of graphite felt (GF)

The GF was initially sonicated in 40 wt% NaOH solution for 30 min and then rinsed with water to neutrality. After this, the pre-washed GF was sonicated in a mixture of HNO_3_ and H_2_SO_4_ (*v*/*v* = 3:1) for a further 30 min, after which it was rinsed with water until neutral. Subsequently, sonication with acetone was continued for 10 min, after which the GF was dried in a vacuum oven at a temperature of 60 °C.

### Preparation of PyCoPc/GF

Four milligrams of PyCoPc was dissolved in a 20 mL mixture of CH_2_Cl_2_ and MeOH (*v*/*v* = 1:1). The treated GF was added and immersed for 5 min. Following this, the GF was retrieved, rinsed with methanol until the filtrate was colorless, and dried in a vacuum oven at 60 °C to obtain PyCoPc/GF.

### Preparation of CoPc/GF

CoPc/GF was prepared in the same way as PyCoPc/GF except that PyCoPc was replaced by commercial cobalt phthalocyanine (CoPc).

### Anodic electrosynthesis of DMF

#### Electrochemical measurement

All the electrochemical measurements were conducted on the CHI760E electrochemical workstation (Shanghai Chen-Hua Instrument Corporation, China). Anodic DMF electrosynthesis was carried out in a three-electrode system. Pt mesh and Ag/AgCl were used as the counter electrode and the reference electrode, respectively. The working electrode dimensions for testing were 1 cm × 2 cm and the loading mass was determined by ICP. For general reaction conditions, a bipolar membrane was employed to separate the compartments of the H-type electrolytic cell (C007-1, Wuhan GaossUnion Technology Co., Ltd.), except during membrane species characterization experiments, where alternative configurations were used. The cathodic compartment was filled with 30 mL of 0.7 M K_2_CO_3_, and the anodic compartment was filled with 15 mL of 0.7 M K_2_CO_3_ and 15 mL of a mixed solution of MeOH/DMA (*v*/*v* = 4:1) with 100 mM KI. Based on literature and prior research, the 0.7 M K_2_CO_3_ electrolyte promotes selective electro-oxidation of methanol to generate the *CH_2_O intermediate^6,11,12^, while a 4:1 MeOH-to-DMA ratio enhances DMF generation. The linear sweep voltammetry (LSV) polarization curves were measured at a scan rate of 10.0 mV s^–1^. ‌Stability testing in an H-type cell was conducted at a constant current density of 50 mA cm^–2^, with periodic replacement of both the anolyte and catholyte every 10 h accompanied by quantitative analysis of the reaction products. During each electrolyte exchange, the electrode was rinsed alternately with methanol and fresh electrolyte, while the membrane surface was rinsed with electrolyte to remove surface-adsorbed products. Upon completion of the test, to eliminate residual electrolytes and reaction products that could interfere with post-test electrode characterization, the electrodes were sequentially subjected to copious rinsing with deionized water, followed by ultrasonication in a beaker for 10 min, and then rinsed thoroughly with methanol three times. Finally, the electrodes were dried overnight in a vacuum oven at 60 °C under continuous vacuum. All electrochemical tests were carried out in triplicate. The error bars in the figures represent the standard deviation of three independent measurements. All electrochemical measurements were carried out at room temperature (25 °C) and atmospheric pressure. All electrolytes were freshly prepared with deionized water and used immediately without further treatment. All electrochemical measurements of the catalysts were conducted without iR compensation.

### Product quantification and identification

After electrolysis, 10 mL of electrolyte was extracted with 5 mL of 1,2-dichlorobenzene for the detection of *N,N,N’,N’-*tetramethylmethanediamine (TMDM). Another 10 mL of electrolyte was extracted with 5 mL of dichloromethane for the detection of DMF. After extraction, the samples were further identified by Shimadzu GCMS-QP2020 GC-MS and quantified by Shimadzu GC-2018 GC featuring an FID detector and HP-5 column. Formate was quantified by ^1^H NMR using H_3_BTC as the internal standard. In detail, the mixture of 500 μL of electrolyte and 100 μL of D_2_O containing 10 mM H_3_BTC solution was used as the test sample.

The FE can be calculated as follows:

1 $${{\mathrm{FE}}}(\%)=100{znF}/Q$$

where *z* is the number of electrons required to form a molecule of product, *n* is the moles of product formed, *F* is the Faraday constant (96485 C mol^–1^) and *Q* is the electric quantity.

The TOF for DMF was calculated as follows:

2 $${{\rm{TOF}}}=\frac{{n}_{{{\rm{DMF}}}}\times {M}_{{{\rm{Co}}}}}{{m}_{{{\rm{Co}}}}\times t}$$

where *n*_DMF_ is the moles of DMF formed (mol), *m*_Co_ is the mass of Co on the GF electrodes determined by ICP (g), *t* is the electrolysis time (s), and *M*_Co_ is the atomic mass of Co (58.93 g mol^–1^).

### Cathodic electrocatalytic CO_2_ reduction (ECR)

The ECR test was performed in a proton exchange membrane (Nafion^®^117) separated H-type electrochemical cell at 25 °C. Pt mesh and Ag/AgCl were used as the counter electrode and the reference electrode, respectively. The working electrode dimensions for testing were 1 cm × 2 cm. Both cathodic and anodic compartments were filled with 30 mL of 0.5 M KHCO_3_. The flow of CO_2_ (99.999%) was 20 mL min^−1^ (using mass flow controller D07-7B) at the cathode during the electrolysis. The gas products were detected by Agilent 7820A GC, while liquid products were quantified by ^1^H NMR.

### Electrolysis-paired tandem synthesis in a H-type cell

#### Electrochemical measurement

Electrolysis-paired tandem synthesis was performed in a bipolar membrane separated H-type electrolytic cell. The electrode dimensions for testing were 1 cm × 2 cm. The cathodic compartment was filled with 30 mL of CO_2_-saturated 0.5 M KHCO_3_, and the anodic compartment was filled with 15 mL of 0.7 M K_2_CO_3_ and 15 mL of a mixed solution of MeOH:DMA (*v*:*v* = 4:1) with 100 mM KI. The flow of CO_2_ (99.999%) was 20 mL min^−1^ (using mass flow controller D07-7B) at the cathode during the electrolysis. A constant current test was used, electrolyzing at –30 mA cm^–2^ for 24 h.

#### CO-relayed tandem reactions for aminocarbonylation

The general conditions for the CO-relayed tandem reactions are as follows: a mixture of 1.0 mmol of aryliodide, 1.2 mmol of amines, 8 mL of toluene, 45 μmol of Pd(OAc)_2_, and 0.1 mmol of DPEphos was stirred at 80 °C for 24 h, under a constant flow of ECR off-gas.

##### *N*-benzoylmorpholine (A1)

^1^H NMR (600 MHz, CDCl_3_): δ (ppm) 7.26–7.41 (m, 5H, Ar-H), 3.43–3.76 (m, 8H, CH_2_) (Supplementary Fig. 49). ^13^C NMR (150 MHz, CDCl_3_): δ (ppm) 170.65, 135.50, 130.09, 128.77, 127.28, 67.10, 48.39, and 42.76 (Supplementary Fig. 50).

##### 4-morpholinyl[4-(trifluoromethyl)phenyl]methanone (A2)

^1^H NMR (600 MHz, CDCl_3_): δ (ppm) 7.69 (d, 2H, Ar-H), 7.52 (d, 2H, Ar-H), 3.79–3.39 (m, 8H, CH_2_) (Supplementary Fig. 51). ^13^C NMR (150 MHz, CDCl_3_): δ (ppm) 168.82, 138.92, 132.06, 127.55, 125.7, 124.63, 122.85, 66.80, 48.22, and 42.77 (Supplementary Fig. 52).

##### 4-(4-methoxybenzoyl)morpholine (A3)

^1^H NMR (600 MHz, CDCl_3_): δ (ppm) 7.37 (d, 2H, Ar-H), 6.90 (d, 2H, Ar-H), 3.81 (s, 3H, OCH_3_), 3.68 (m, 8H, CH_2_) (Supplementary Fig. 53). ^13^C NMR (150 MHz, CDCl_3_): δ (ppm) 170.41, 160.87, 129.19, 127.29, 113.81, 66.77, 55.40, 48.42, and 42.71 (Supplementary Fig. 54).

##### N-cyclohexyl-4-methoxybenzamide (A4)

^1^H NMR (400 MHz, CDCl_3_): δ (ppm) 7.71 (d, 2H, Ar-H), 6.87 (d, 2H, Ar-H), 6.12 (s, 1H, N-H), 3.82 (m, 1H, CH), 3.81 (m, 3H, OCH_3_), 1.98 (m, 2H, CH_2_), 1.61–1.73 (m, 3H, CH_2_), 1.17–1.39 (m, 5H, CH_2_) (Supplementary Fig. 55). ^13^C NMR (100 MHz, CDCl_3_): δ (ppm) 166.25, 162.00, 128.73, 127.36, 113.67, 55.44, 48.70, 33.32, 25.64, and 25.04 (Supplementary Fig. 56).

##### (2,3-dihydro-1,4-benzodioxin-6-yl)−4-morpholinylmethanone (A5)

^1^H NMR (600 MHz, CDCl_3_): δ (ppm) 6.94 (d, 1H), 6.90 (d, 1H), 6.87 (d, 1H), 4.26 (m, 4H, CH_2_), 3.67 (m, 8H, CH_2_) (Supplementary Fig. 57). ^13^C NMR (150 MHz, CDCl_3_): δ (ppm) 170.02, 145.16, 143.5, 128.41, 117.39, 67.00, 64.55, and 64.38 (Supplementary Fig. 58).

### Electrolysis-paired tandem synthesis in a flow cell

#### Preparation of PyCoPc/GF-GDE

GF was sprayed with 15 wt% PTFE and dried at 60 °C. Then, the PTFE-coated GF was further sprayed with a mixture of 30 wt% PTFE and 250 mg of carbon black dispersed in 50 mL of ethanol. After drying, the composite was subjected to thermal annealing in a tube furnace under Ar atmosphere using a programmed heating protocol: 200 °C for 10 min followed by 320 °C for 60 min, resulting in GF-GDE formation. Finally, GF-GDE was immersed in a methanol solution of PyCoPc (0.2 mg mL^–1^) for 5 min, then retrieved and rinsed with methanol, and dried at 60 °C to obtain PyCo/Pc-GF-GDE.

### Electrochemical test

Flow cell test was performed in a bipolar membrane separated flow cell. PyCoPc/GF and PyCoPc/GF-GDE were used as the anode and cathode electrocatalytic electrodes, respectively. The electrode dimensions for testing were 2 cm × 2 cm. The cathodic compartment was filled with 60 mL of 1 M KOH. The anodic compartment was filled with 30 mL of 0.7 M K_2_CO_3_ and 30 mL of a mixed solution of MeOH:DMA (*v*:*v* = 4:1) with 100 mM KI. During the test, the electrolytes were circulated by peristaltic pumps. The flow of CO_2_ (99.999%) was 20 mL min^−1^ (using mass flow controller D07-7B) at the cathode. A constant current test was used, electrolyzing at –200 mA cm^–2^ for 24 h. The cathodic ECR off-gas was fed into the subsequent thermally catalyzed reaction, represented by the synthesis of **A5**, scaling up 6-iodo-1,4-benzodioxane to 1.0 g and the rest of the reaction reagents were scaled up proportionally.

### In situ attenuated total reflection-infrared measurement

The in situ attenuated total reflection-infrared (ATR-IR) spectra were collected in a single electrochemical cell by using a typical three-electrode system at 0.5 V (vs. Hg/HgO) with varying electrolysis times. The Pt wire and Hg/HgO electrode were used as the counter and reference electrodes, respectively. PyCoPc/GF was crushed and formulated into a slurry, which was then dropped on the glass carbon electrode. The glass carbon electrode was pressed close to the Ge single crystal to ensure obtaining better IR signals. The electrolyte was a mixture of 0.7 M K_2_CO_3_ and MeOH:DMA (*v*:*v* = 4:1) with or without 100 mM KI.

### Reaction pathway analysis experiment

#### Control experiments for iodide-facilitated DMF synthesis

The standard reaction system contained 29 mL of 0.7 M K_2_CO_3_ and 1 mL TMDM, magnetically stirred at ambient temperature for 3 h. Reaction variants included: (1) supplementation with 50 mM I_2_, (2) constant-current electrolysis at 50 mA cm^–2^, or (3) addition of 100 mM DMPO spin trap, as required by specific experimental protocols.

### Control experiments for identifying key reaction intermediates

The standard reaction system contained 29 mL of 0.7 M K_2_CO_3_ and 1 mL of TMDM with 0.1 M KI and was electrolyzed at 50 mA cm^–2^ for 3 h. For comparative studies, TMDM was replaced with equimolar amounts of tetramethylurea, bis(dimethylamino)methanol, or tetramethyl-chloroformamidinium chloride while maintaining all other conditions.

### Quasi-in situ ^1^H NMR test

A mixture of CDCl_3_ (550 μL) and TMDM (100 μL) was prepared in an NMR tube. After recording the initial ^1^H NMR spectrum, I_2_ (10 mg) was added, and the reaction was monitored by ^1^H NMR.

### Control experiments for iminium ion intermediate conversion to DMF

Control experiments were systematically performed under three different conditions while maintaining a constant amount of (dimethylaminomethylene) dimethylammonium chloride (7.11 mmol): (1) treatment with 30 mL of pure H_2_O under continuous stirring for 3 h, (2) treatment with 30 mL of 0.7 M K_2_CO_3_ aqueous solution under continuous stirring for 0.5 h and (3) electrochemical conversion in 30 mL of 0.7 M K_2_CO_3_ under identical electrolytic conditions (50 mA cm^–2^, 0.5 h) with PyCoPc/GF electrode.

### Computational details

The density functional theory (DFT) calculations were performed using the Vienna Ab Initio Simulation Package (VASP) employing the Perdew–Burke–Ernzerhof (PBE) exchange-correlation functional within the generalized gradient approximation (GGA) and the projector augmented wave (PAW) method^48–52^. For all simulations, we adopted convergence criteria of 1 × 10^–5^ eV/atom for the self-consistent field calculations and 0.02 eV/Å for ionic relaxation. Our catalyst model was constructed by PyCoPc on a graphene substrate layer. Following structural optimization of this system, we performed adsorption energy calculations. The plane-wave basis set cutoff energy was fixed at 500 eV, and we used a 1 × 1 × 1 **k**-point mesh for the in-plane hybrid model simulations. The Gibbs free energy change (Δ*G*) was calculated using the expression:

3 $$\Delta G={\Delta E}_{{\mathrm{DFT}}}+\Delta {\mathrm{ZPE}}\,-\,T\Delta S$$

where *E*_DFT_, ZPE and *S* are total energy from DFT calculations, zero-point energy, and the entropy, respectively^53–55^.

## Supplementary information

Supplementary Information

Description of Additional Supplementary Files

Supplementary Data 1

Transparent Peer Review File

## Data Availability

The data supporting the findings of this study are available within the article and the Supplementary Information, and can also be obtained from the corresponding author upon reasonable request. The single-crystal structure of A5 is archived at the Cambridge Crystallographic Data Centre under the reference number CCDC-2442656. This data can be obtained free of charge from The Cambridge Crystallographic Data Centre via www.ccdc.cam.ac.uk/data_request/cif. Source data are provided with this paper. The material characterizations and electrocatalytic performance data generated in this study have been deposited in the Figshare database 10.6084/m9.figshare.31569046^56^.
