# Supplementary material for: Redox-mediated domino electrosynthesis of N,N-dimethylformamide with industrial-relevant productivity and modularized cathodic integration
Source: Nat Commun. 2026 Apr 17;17:5349. doi: 10.1038/s41467-026-71637-z (PMC13272958; doi:10.1038/s41467-026-71637-z)
Supplement: Supplementary file 1 — Supplementary Information [file 41467_2026_71637_MOESM1_ESM.pdf]

Supporting Information for

Redox-Mediated Domino Electrosynthesis of *N,N*-Dimethylformamide with Industrial-Relevant Productivity and Modularized Cathodic Integration

Yingchun He[1,2,3], Qing Li[1], Shao Zhang[1,3], Bo Zhang[1,3], Dong-Dong Ma[4]*, Xin-Tao Wu[1,3], Qi-Long Zhu[1,2]*

[1] State Key Laboratory of Structural Chemistry, Fujian Institute of Research on the Structure of Matter, Chinese Academy of Sciences, Fuzhou 350108, China

[2] School of Materials Science and Engineering, Zhejiang Sci-Tech University, Hangzhou 310018, China

[3] University of Chinese Academy of Science, Beijing 100049, China

[4] Hubei Key Laboratory of Low Dimensional Optoelectronic Materials and Devices, Hubei University of Arts and Science, Xiangyang, Hubei 441053, China

*Corresponding Author: Dong-Dong Ma and Qi-Long Zhu

*E-mail: maddjdld@163.com, qlzhu@fjirsm.ac.cn

Supplementary Figures

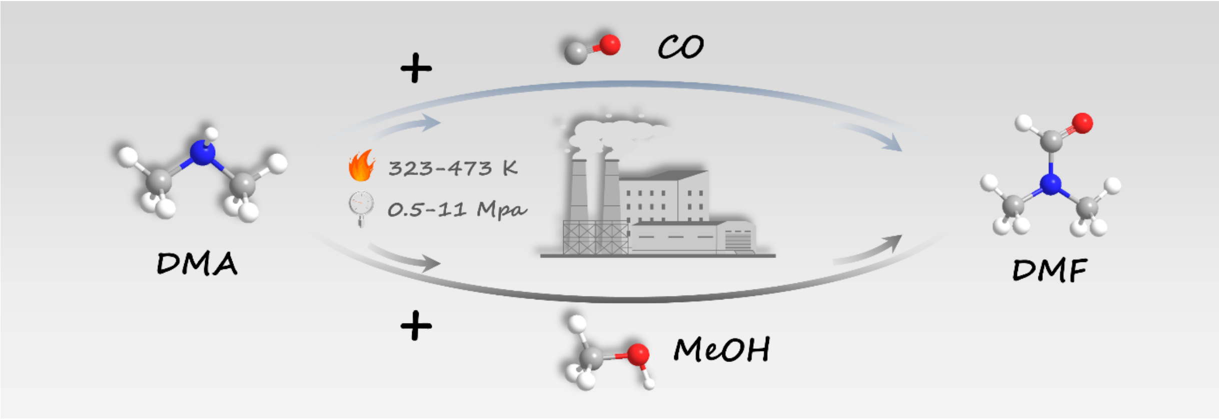

Supplementary Fig. 1 Schematic diagram for the conventional thermochemical strategy to produce DMF.

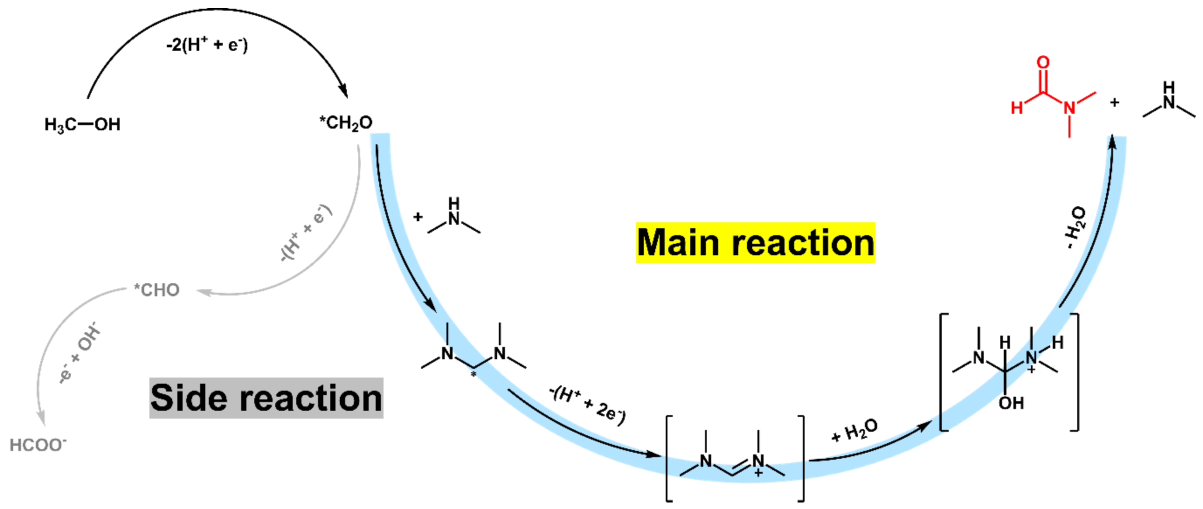

Supplementary Fig. 2 Proposed reaction mechanism of electrosynthesis of DMF from methanol and DMA.

It should be noted that formaldehyde and DMA can rapidly react to form TMDM,[1, 2] making direct quantification of formaldehyde difficult, especially in the presence of high DMA concentrations.

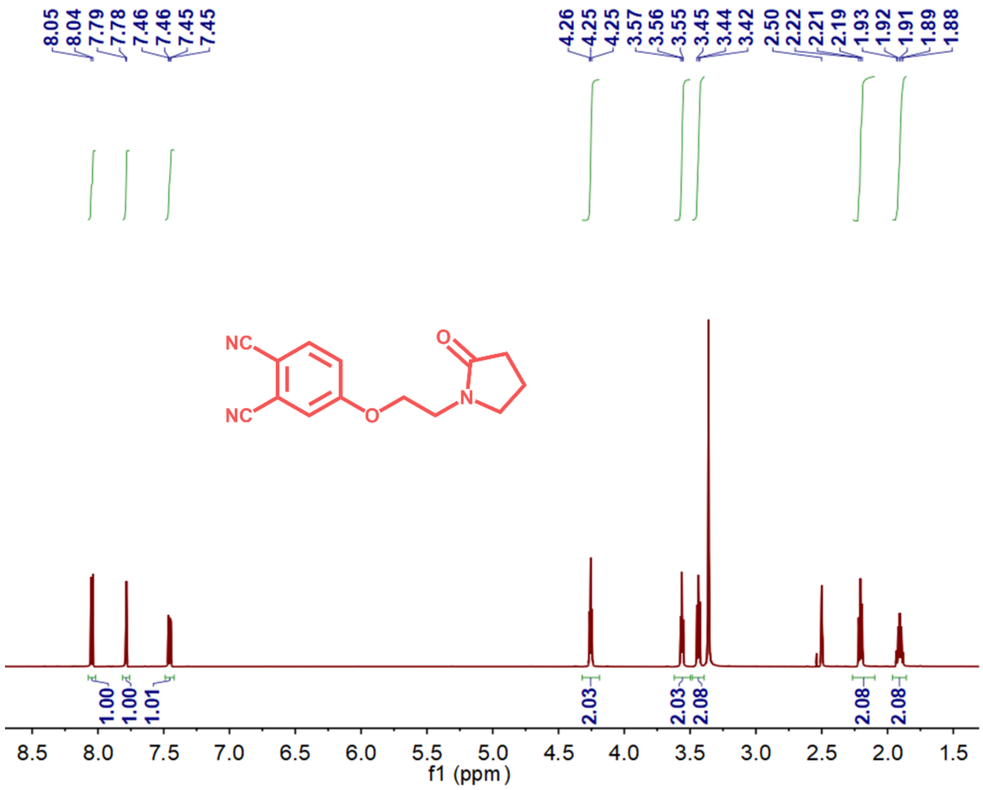

Supplementary Fig. 3 ^1H NMR spectrum of Py-CN_2 (600 MHz, DMSO-d_6).

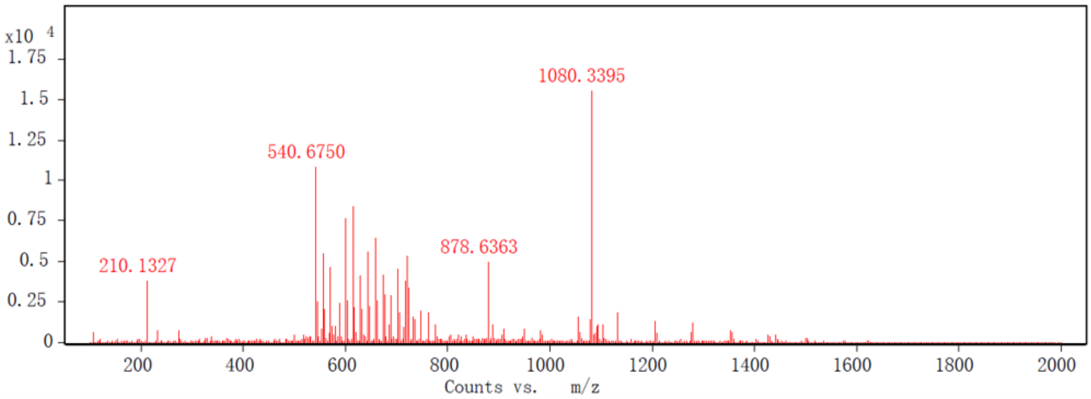

Supplementary Fig. 4 ESI-MS of PyCoPc.

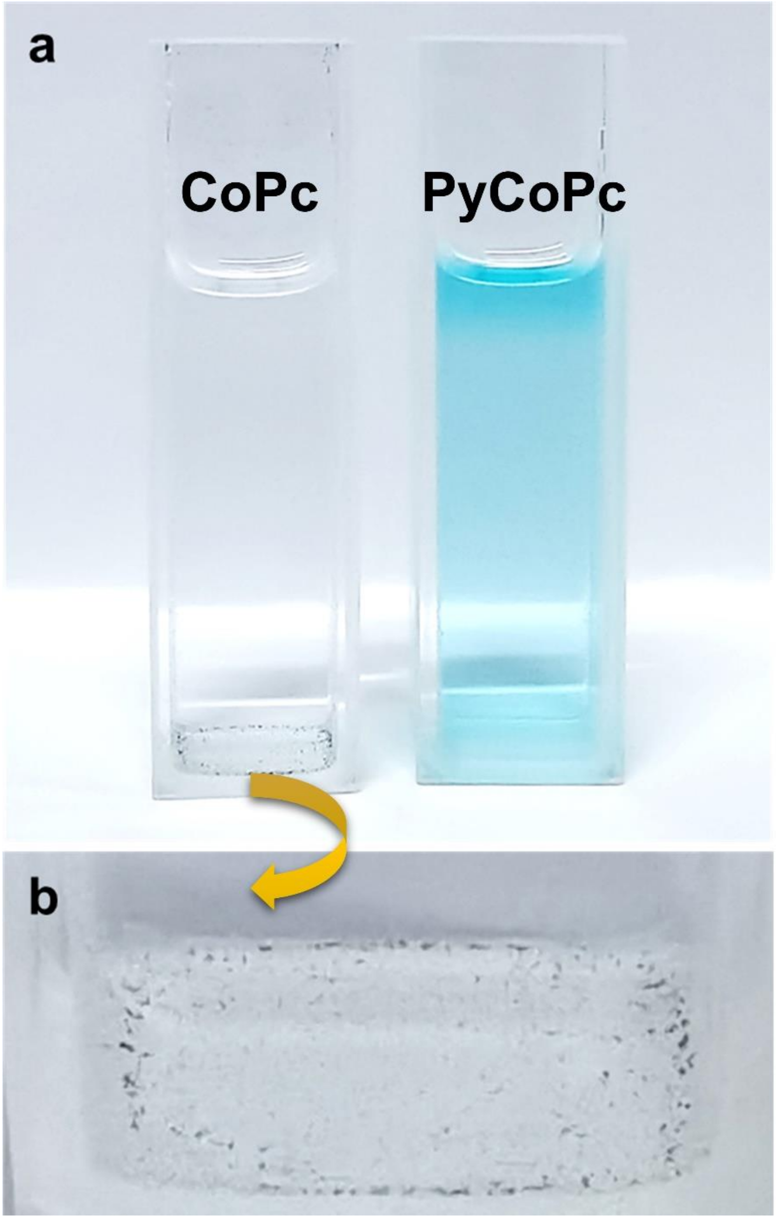

Supplementary Fig. 5 (a, b) Optical photographs of CoPc and PyCoPc in the mixed solvent of methanol and DCM.

CoPc has a poor solubility with the solid precipitate. (C: 4×10^{-3} mg mL^{-1}, v_{MeOH}:v_{DCM} = 1:1).

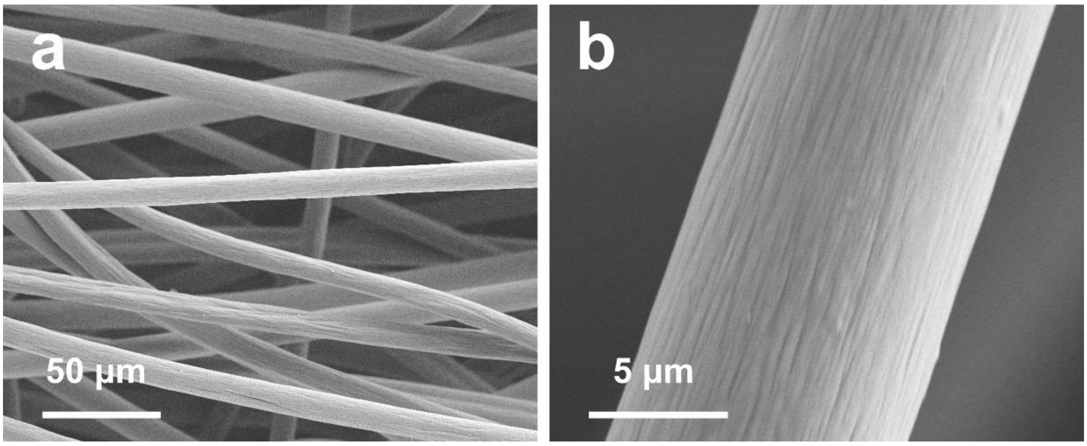

Supplementary Fig. 6 (a, b) SEM images of GF.

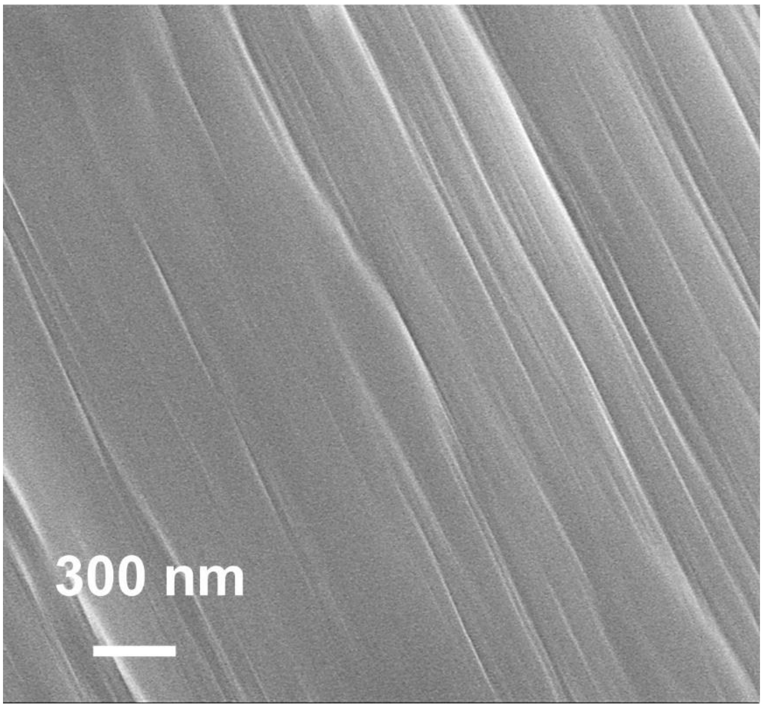

Supplementary Fig. 7 SEM image of PyCoPc/GF with high magnification.

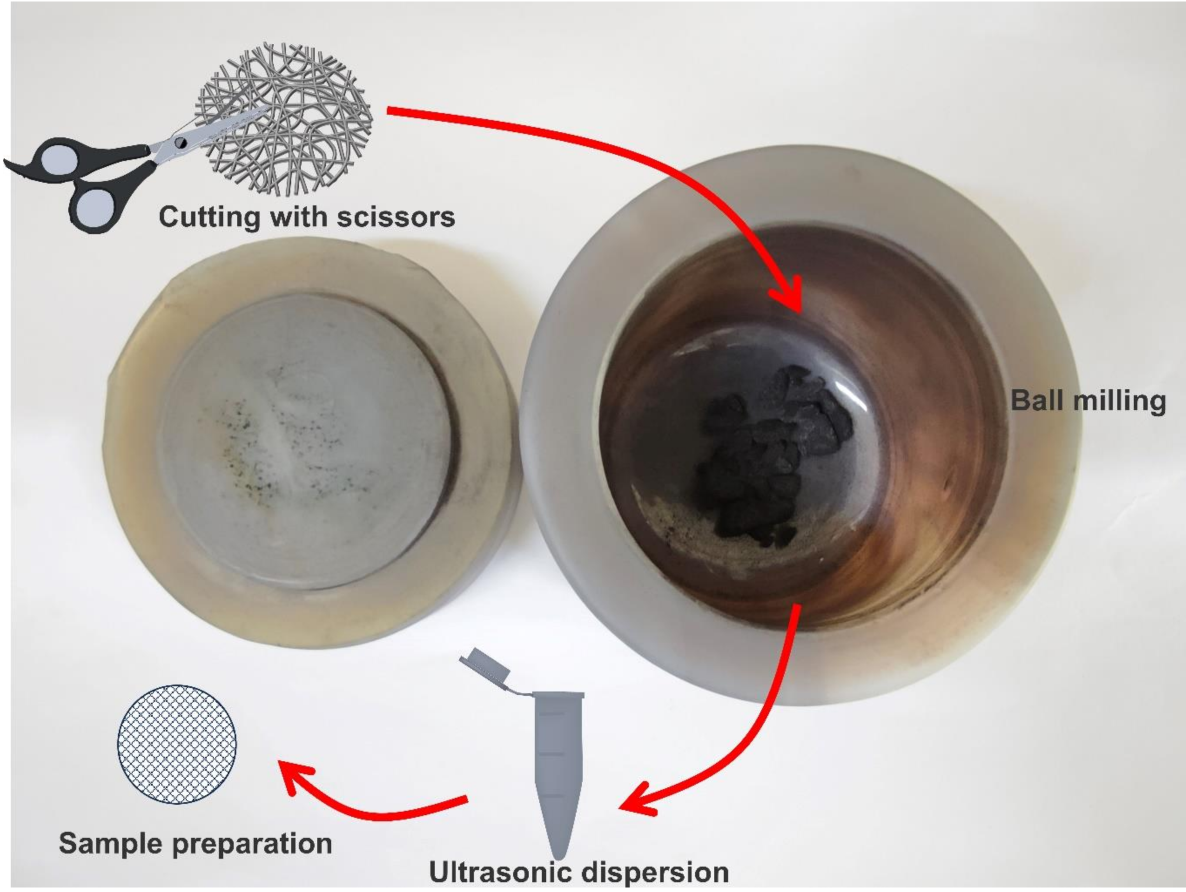

Supplementary Fig. 8 Schematic of ball milling-based sample preparation.

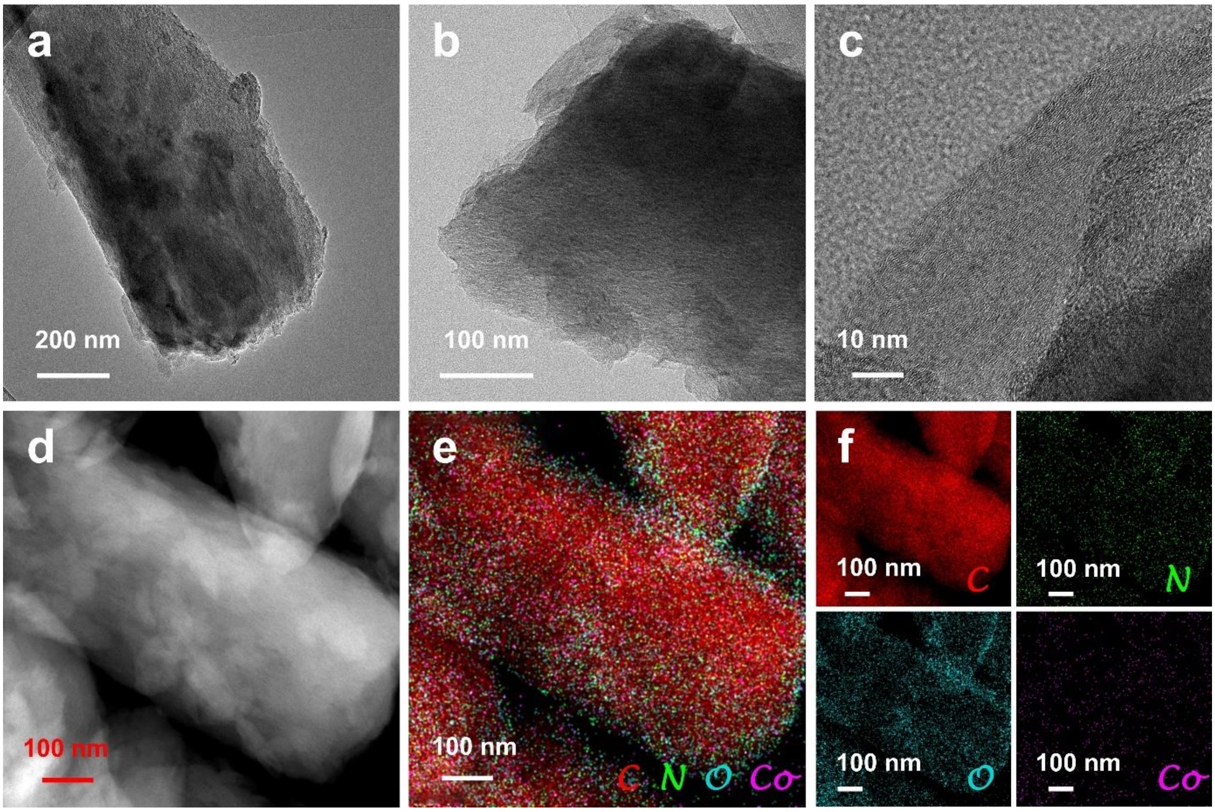

Supplementary Fig. 9 (a, b) TEM images; (c) HRTEM image; (d) HADDF-STEM image and (e, f) corresponding EDX elemental mapping images of PyCoPc/GF after ball milling.

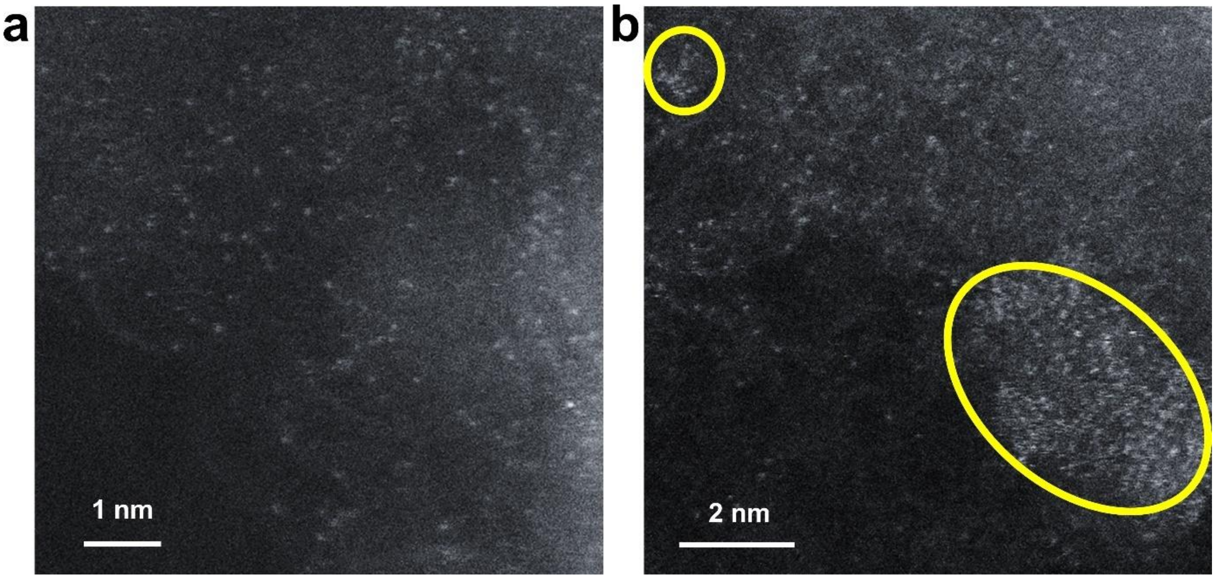

Supplementary Fig. 10 AC-HAADF-STEM images of (a) PyCoPc/GF, (b) CoPc/GF.

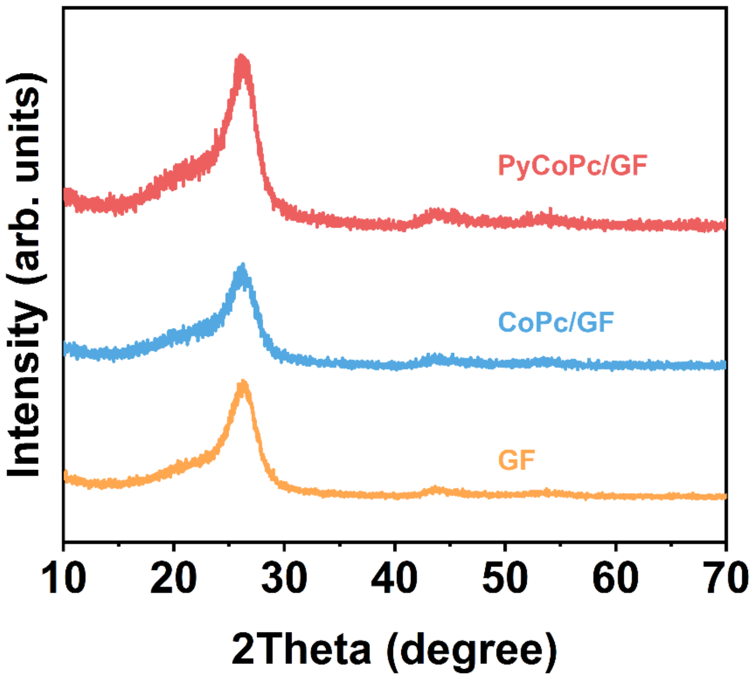

Supplementary Fig. 11 XRD patterns of PyCoPc/GF, CoPc/GF and GF.

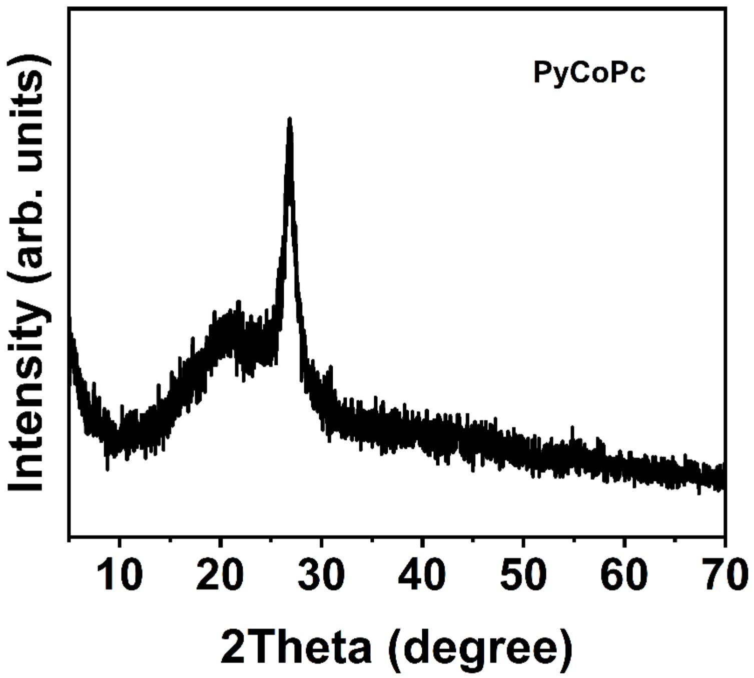

Supplementary Fig. 12 XRD pattern of PyCoPc.

It should be noted that the peak at 27° can be attributed to the π-π stacking of PyCoPc molecules.[3, 4]

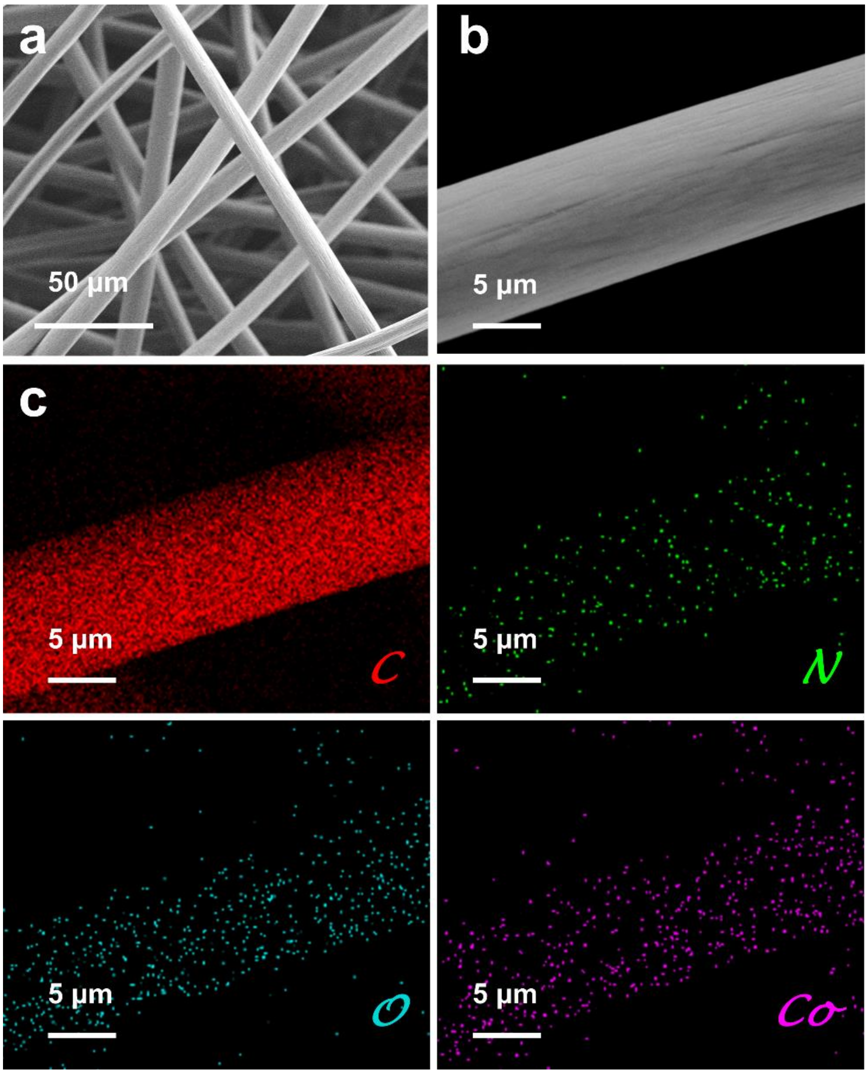

Supplementary Fig. 13 (a,b) SEM images of CoPc/GF and (c) corresponding EDX elemental mapping images.

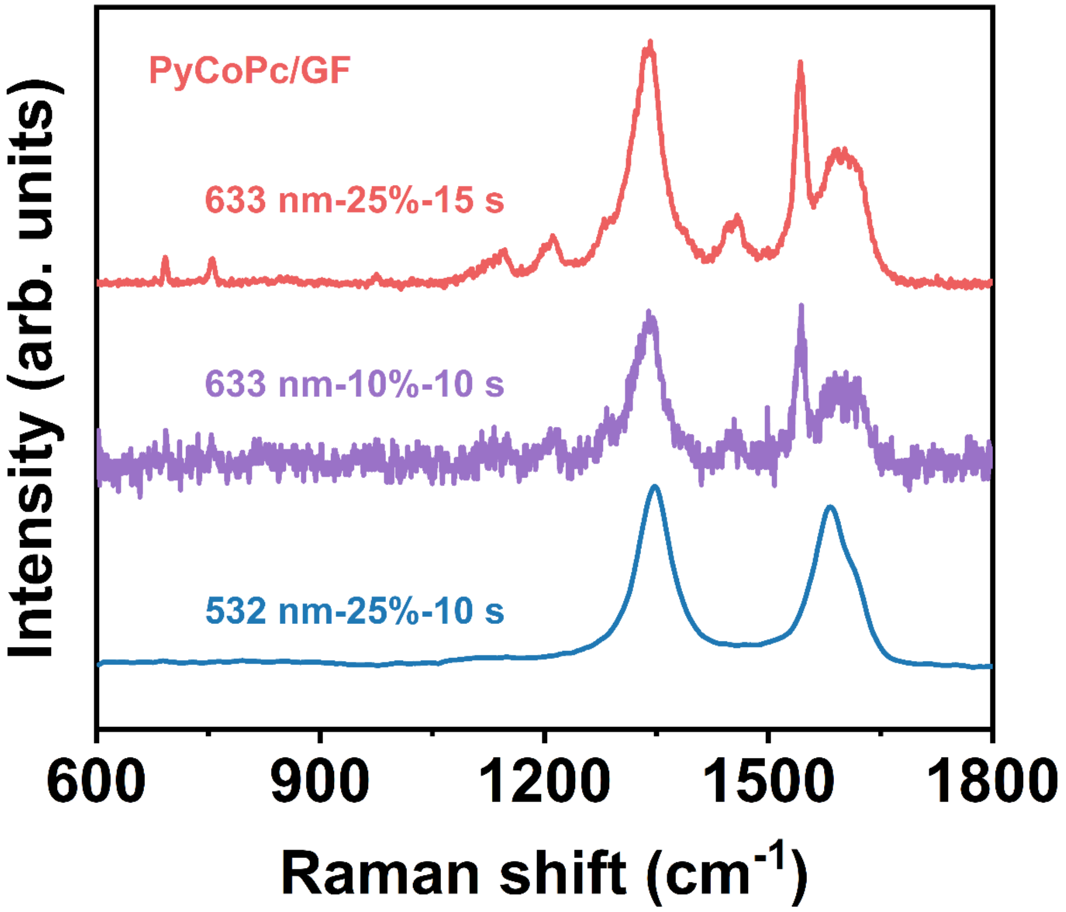

Supplementary Fig. 14 Raman spectra of PyCoPc/GF at different testing conditions.

Notably, the characteristic Raman signals of the phthalocyanine moiety in PyCoPc/GF are exclusively detectable using a 633 nm laser source. Note: 633 nm: laser wavelength, 25%: laser power, 15 s: exposure time.

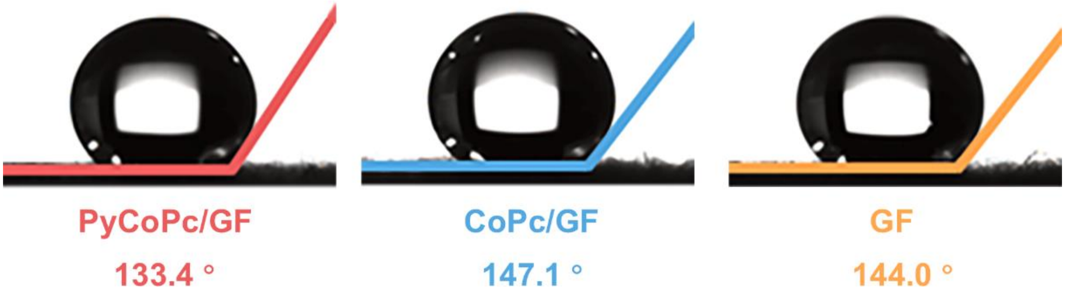

Supplementary Fig. 15 Water contact angle patterns of PyCoPc/GF, CoPc/GF and GF.

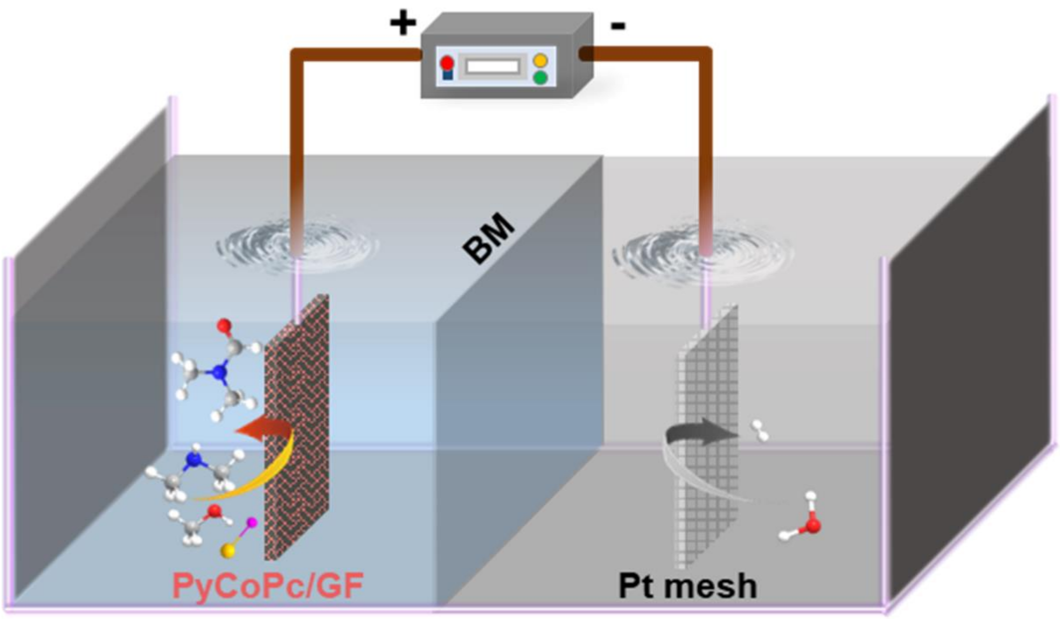

Supplementary Fig. 16 Schematic illustration of anodic electrosynthesis of DMF in the H-type cell.

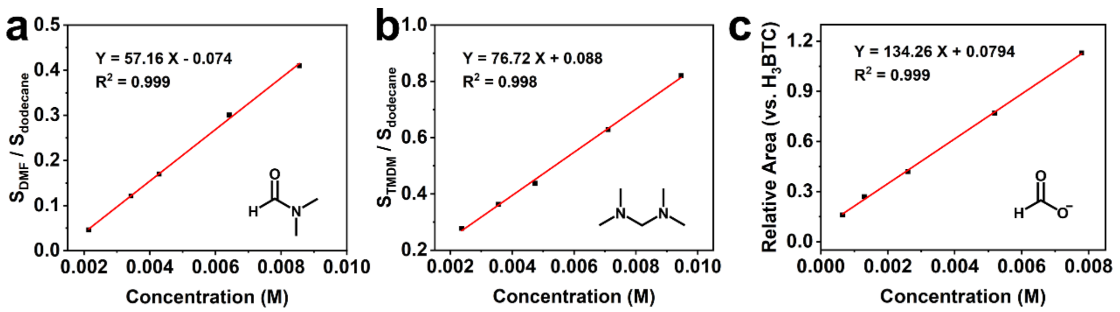

Supplementary Fig. 17 Standard curves of (a) DMF, (b) TMDM by GC and (c) formate by ^{1}H NMR spectroscopy.

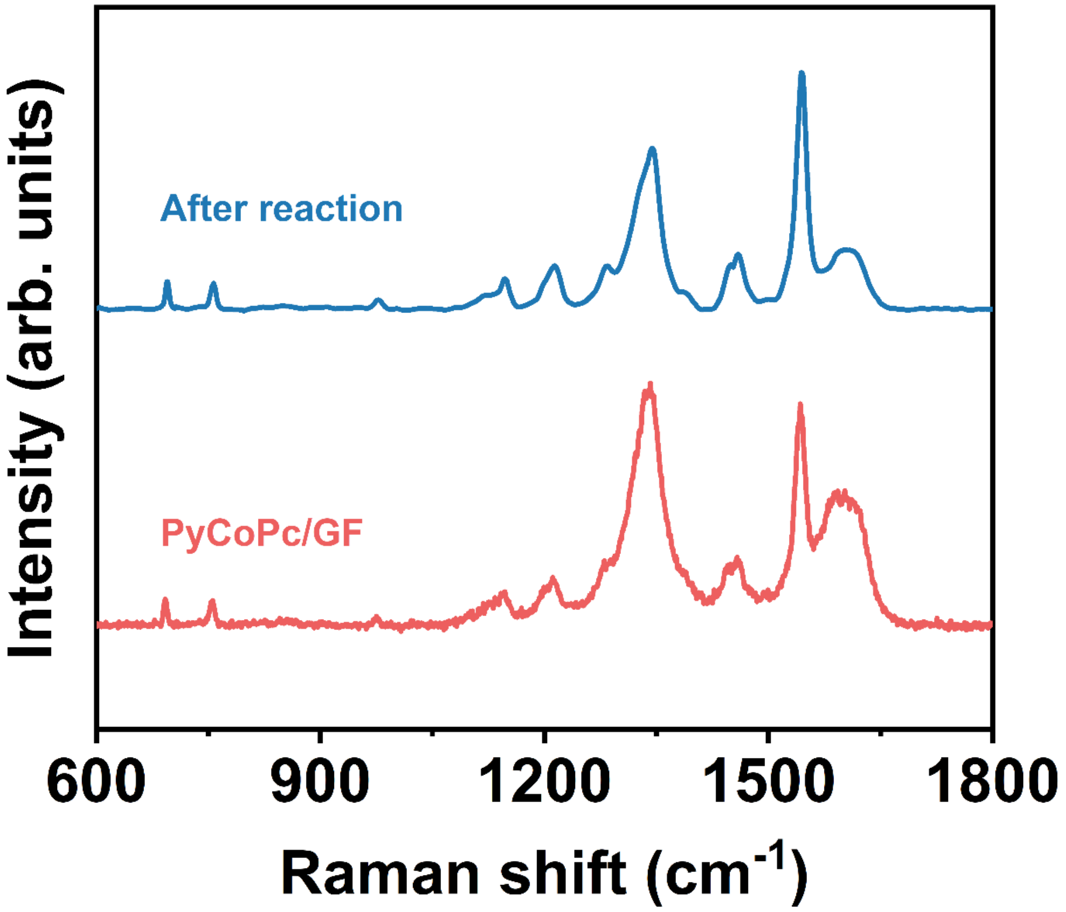

Supplementary Fig. 18 Raman spectra of PyCoPc/GF before and after CV activation in 0.7 M K_2CO_3 at 0–1 V (vs. Hg/HgO) under the 633 nm laser excitation.

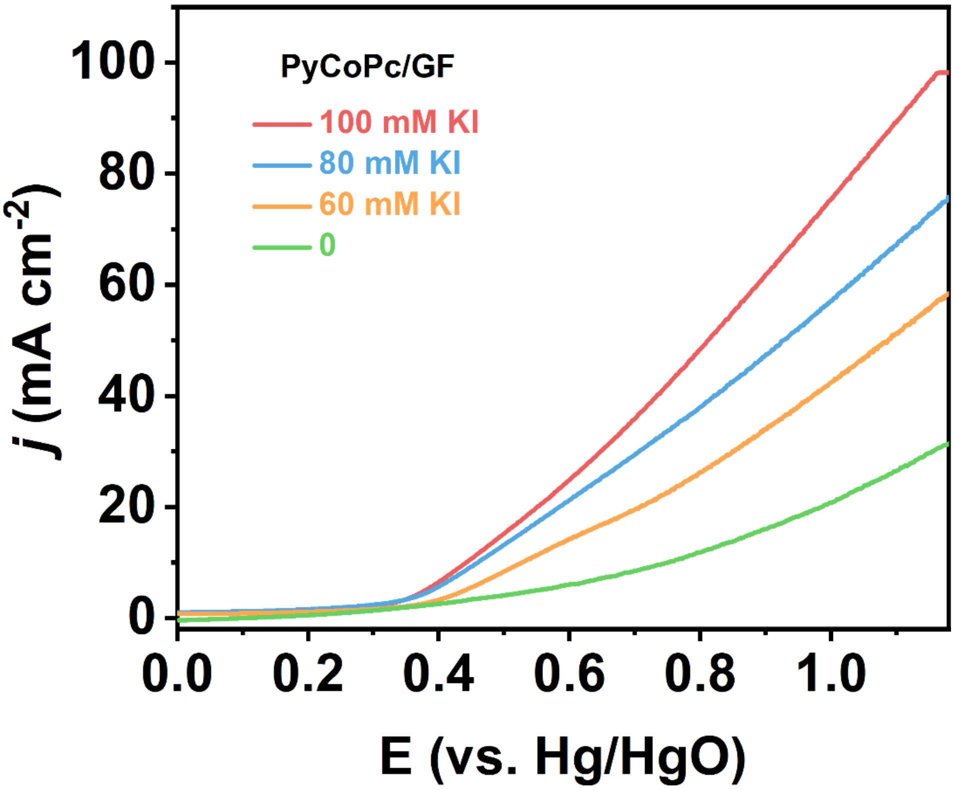

Supplementary Fig. 19 LSV curves of PyCoPc/GF with different concentrations of KI.

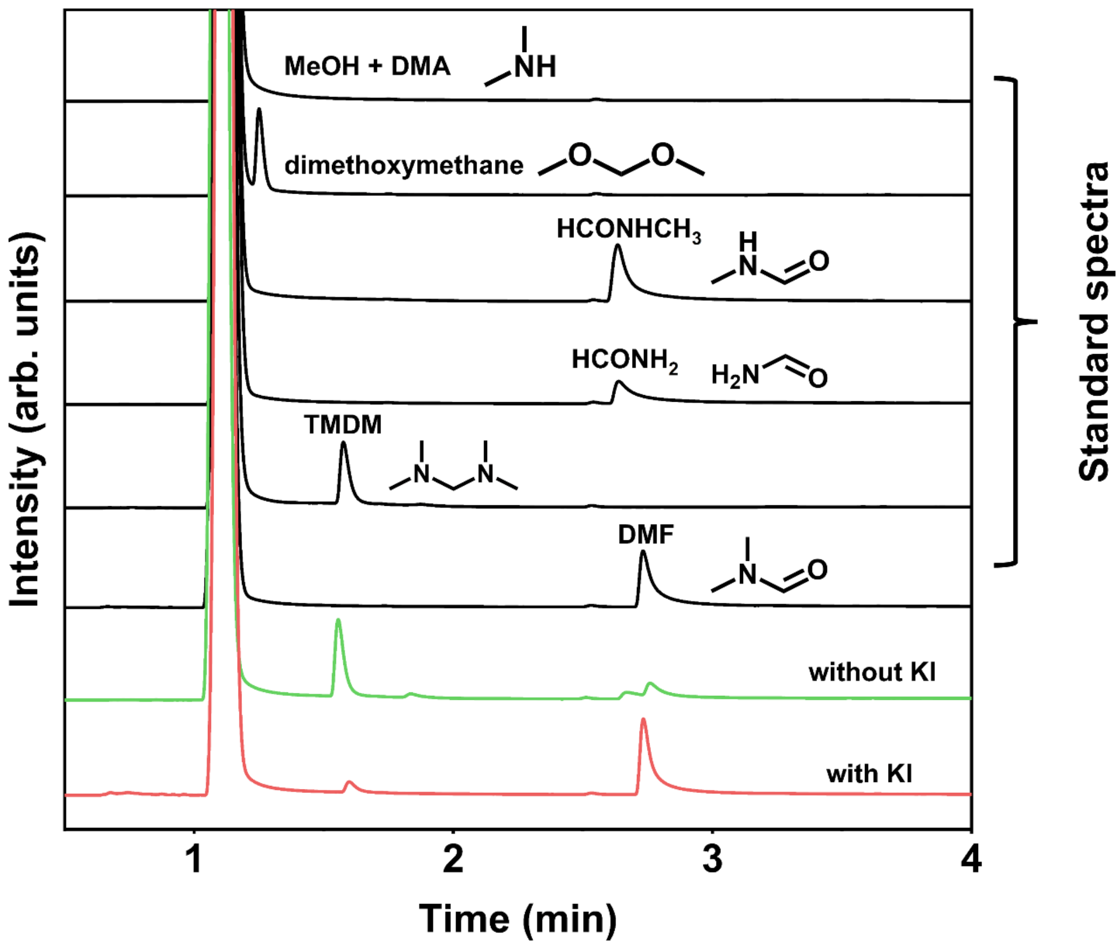

Supplementary Fig. 20 GC spectra of proposal products and electrolysis products in 0.7 M K_2CO_3 with or without 100 mM KI at 50 mA cm^{-2}.

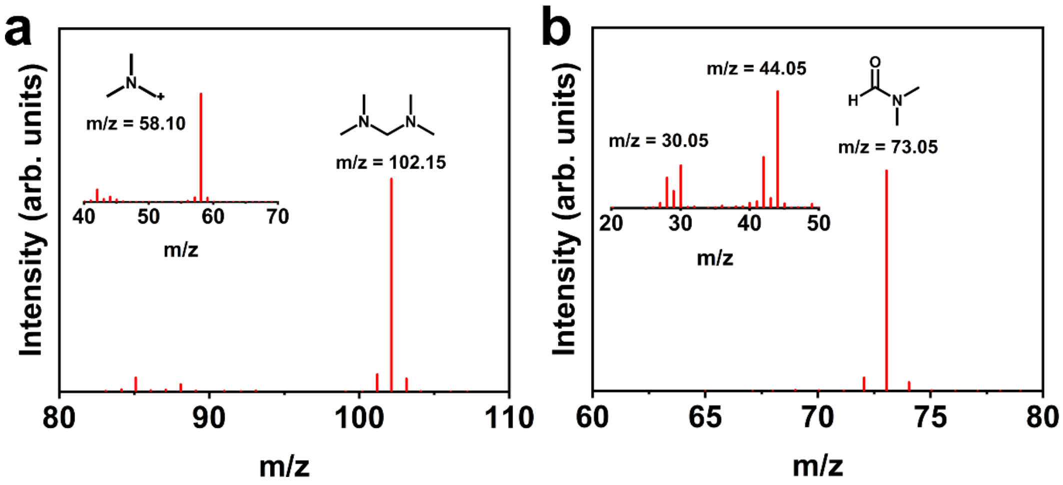

Supplementary Fig. 21 GC-MS of (a) TMDM and (b) DMF in electrolyte after the electrochemical reaction at 50 mA cm^{-2} in 0.7 M K_2CO_3 with 100 mM KI.

It is noteworthy that TMDM undergoes fragmentation during GC-MS analysis, yielding a characteristic fragment ion peak at m/z = 58.10.

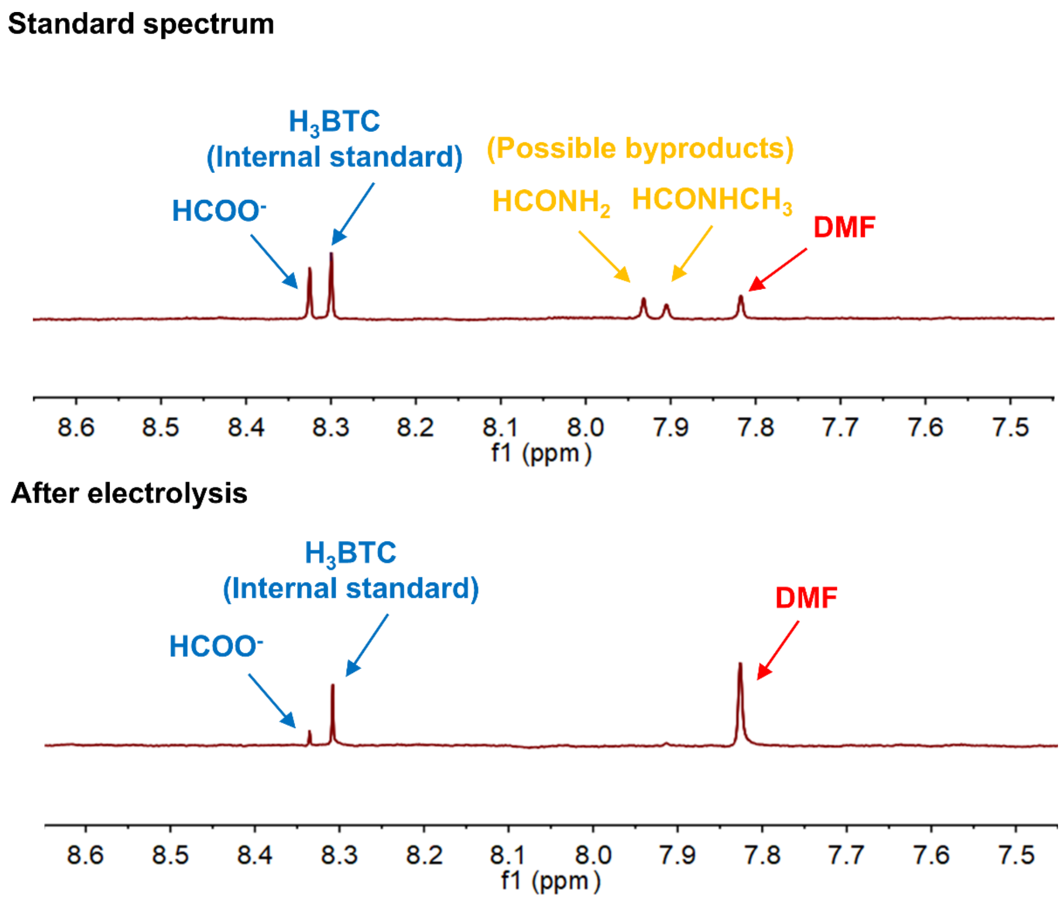

Supplementary Fig. 22 ^{1}H NMR spectra of standard samples and recycled electrolyte after electrolysis at 50 mA cm^{-2} in 0.7 M K_2CO_3 with 100 mM KI.

It should be noted that possible reaction by-products such as *N*-methylformamide and formamide are virtually absent in the reaction products.

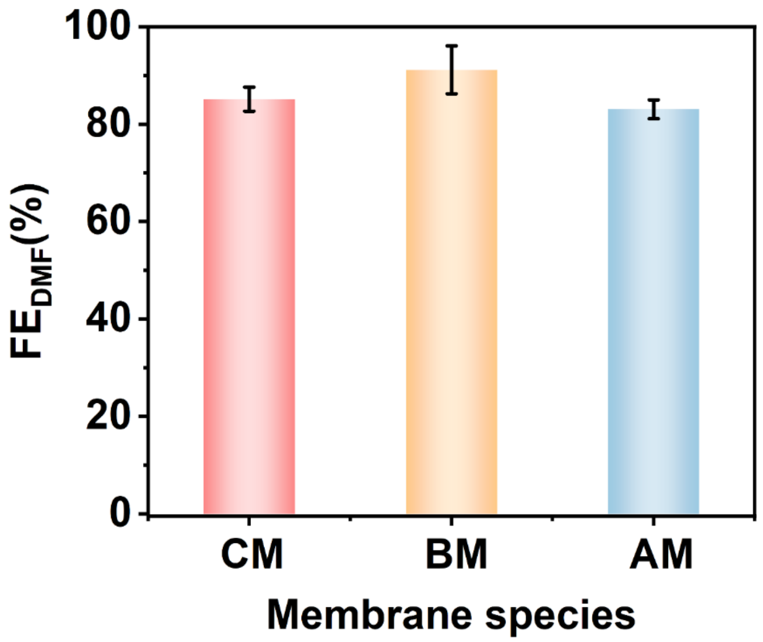

Supplementary Fig. 23 FE_{DMF} of PyCoPc/GF at 50 mA cm^{-2} in 0.7 M K_2CO_3 with 100 mM KI using different membrane.

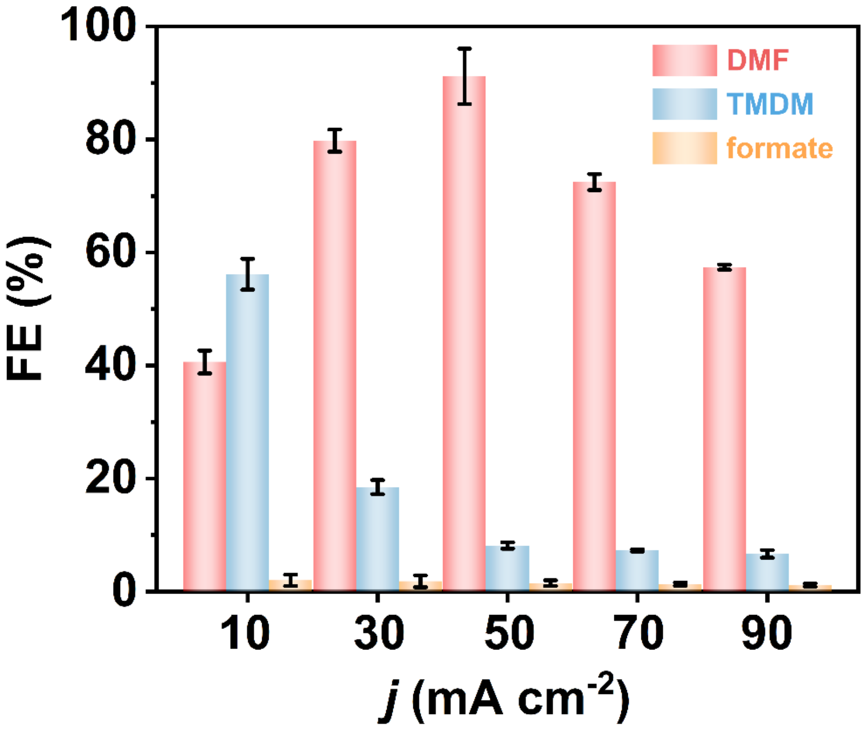

Supplementary Fig. 24 FE of products over PyCoPc/GF at different current densities in 0.7 M K_2CO_3 with 100 mM KI.

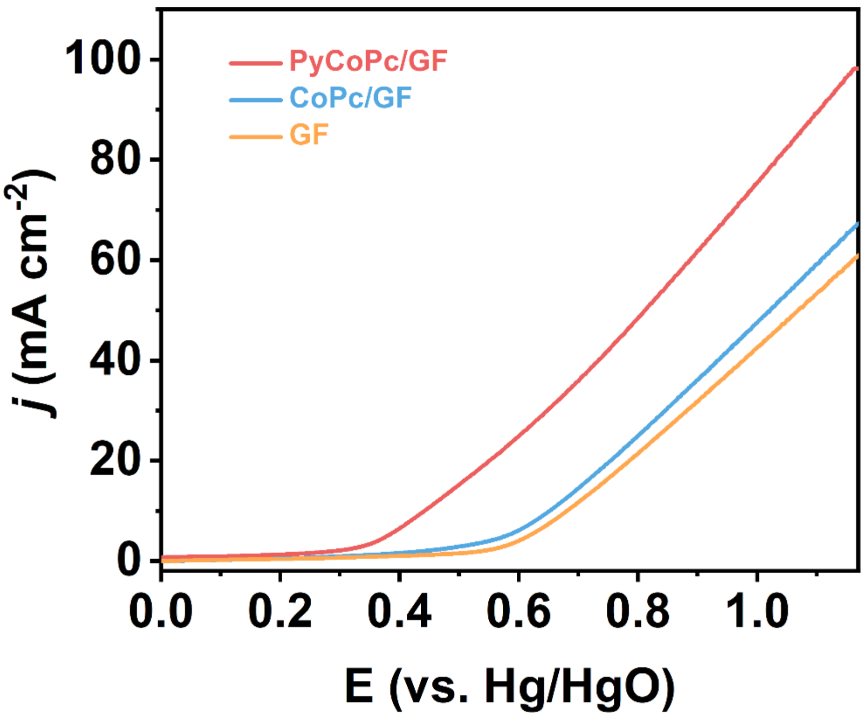

Supplementary Fig. 25 LSV curves of PyCoPc/GF, CoPc/GF and GF in 0.7 M K_2CO_3 with 100 mM KI.

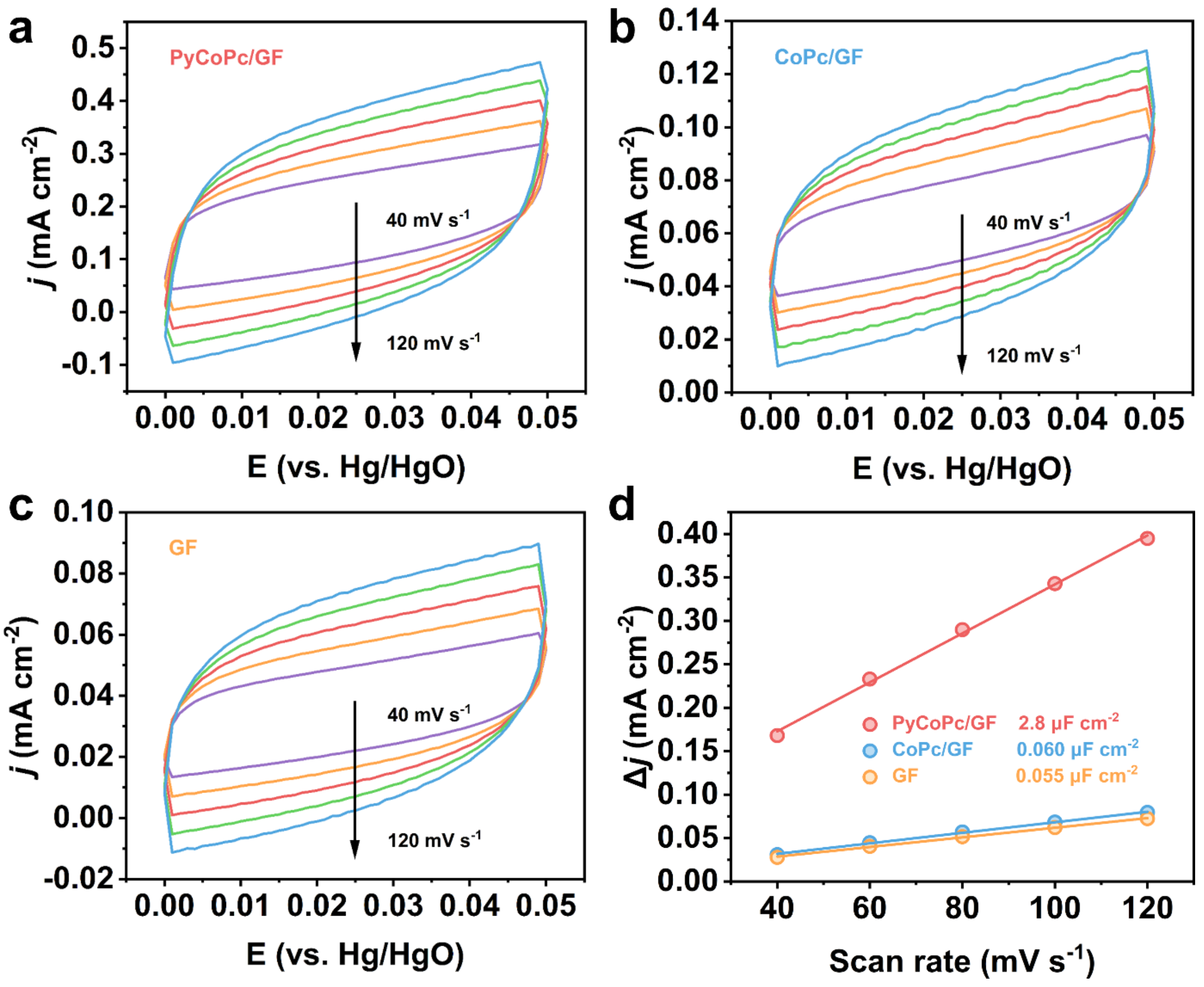

Supplementary Fig. 26 CV curves of (a) PyCoPc/GF, (b) CoPc/GF and (c) GF at different scan rates; (d) charge current density difference against scan rates.

The electrochemically active surface areas (ECSAs) can be used to evaluate the degree of exposure of active sites in catalysts and are typically proportional to the double-layer capacitance (C_{dl}). C_{dl} measurements were performed under the same electrolyte and cell configuration as those employed for the anodic electrosynthesis of DMF. As shown in Supplementary Fig. 26d, PyCoPc/GF exhibits a significantly higher C_{dl} value than CoPc/GF and GF, indicating enhanced accessibility of active sites. This improvement is attributed to the pyrrolidone groups facilitating the anchoring of PyCoPc molecules onto the GF substrate, consistent with ICP-OES results (Supplementary Table 2).

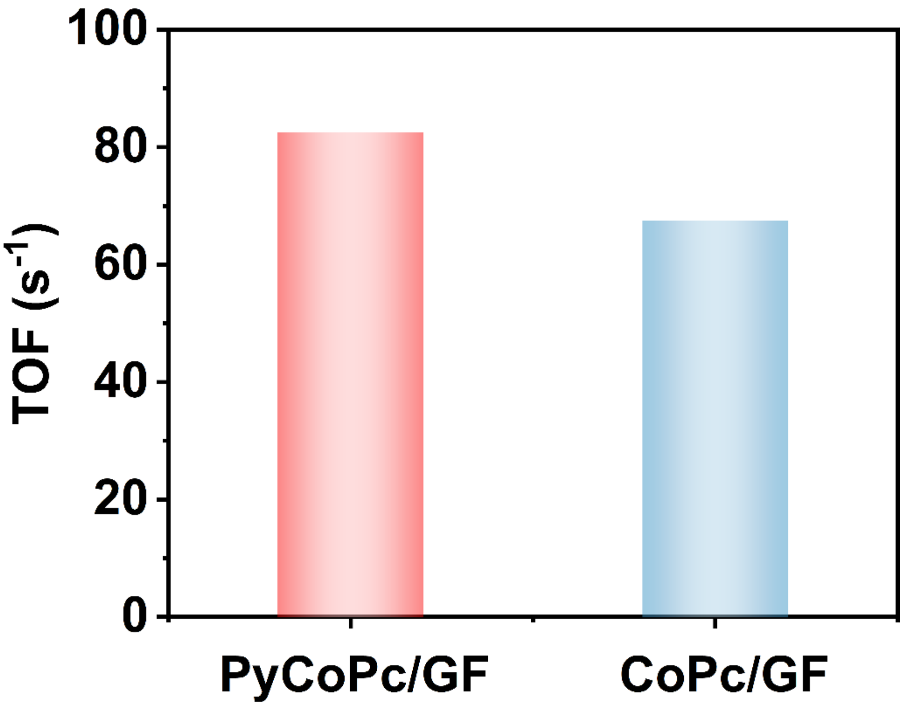

Supplementary Fig. 27 TOF values of PyCoPc/GF and CoPc/GF for DMF production.

It should be noted that the Co content determined by ICP can be approximated as the active site count in the TOF calculation.[5, 6]

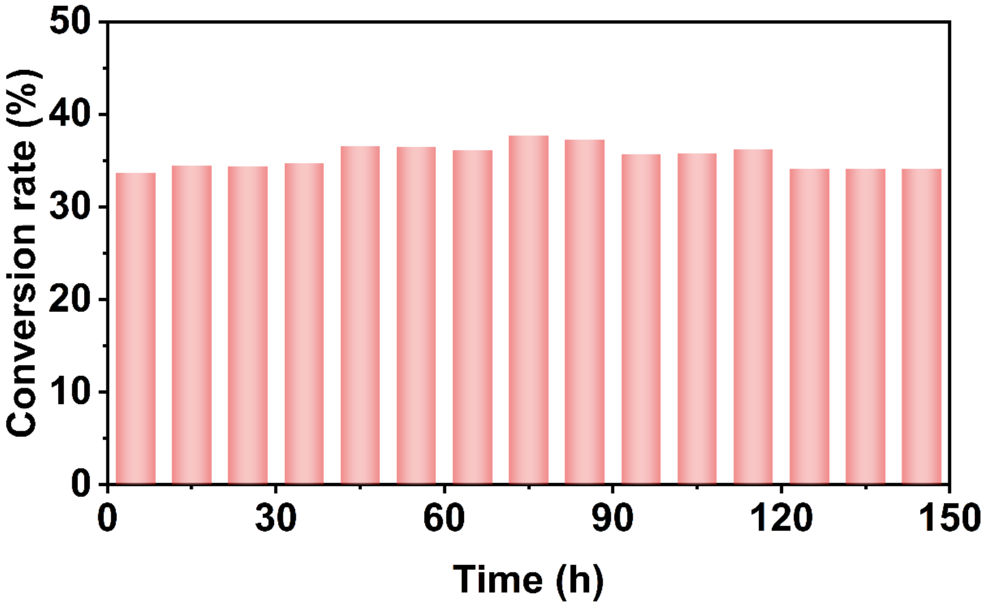

Supplementary Fig. 28 DMA conversion rate of PyCoPc/GF during the stability test at 50 mA cm^{-2}.

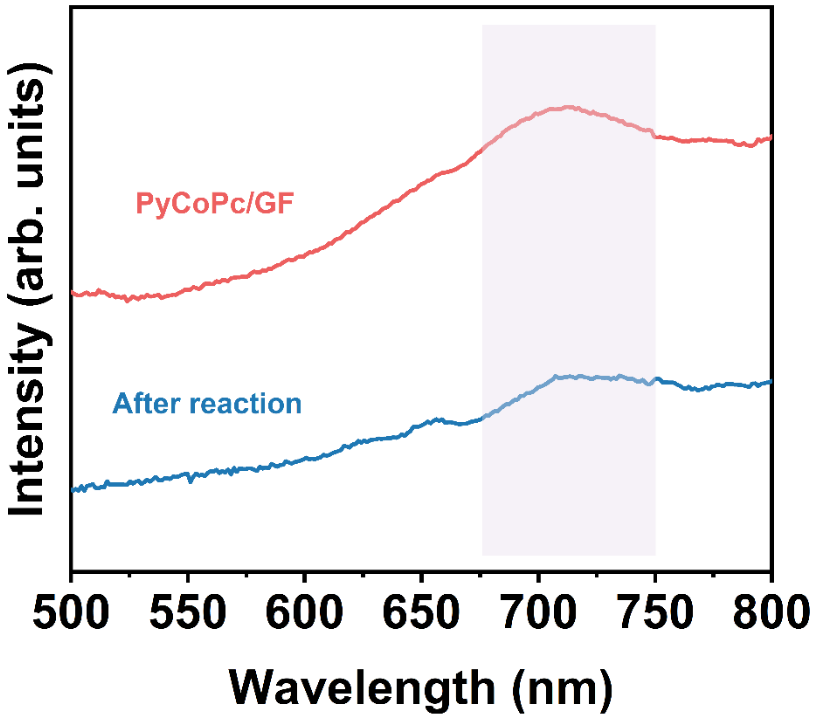

Supplementary Fig. 29 UV-Vis spectra of PyCoPc/GF before and after reaction.

Prior to characterization of the post-reaction catalyst, samples were sequentially rinsed with deionized water and methanol, followed by vacuum drying to remove surface contaminants. Detailed procedures are documented in the Experimental Section.

The Py-CoPc/GF electrode was subjected to pulverization and dispersion in methanol solution for characterization. After reaction, the characteristic phthalocyanine peak remained detectable at approximately 710 nm.

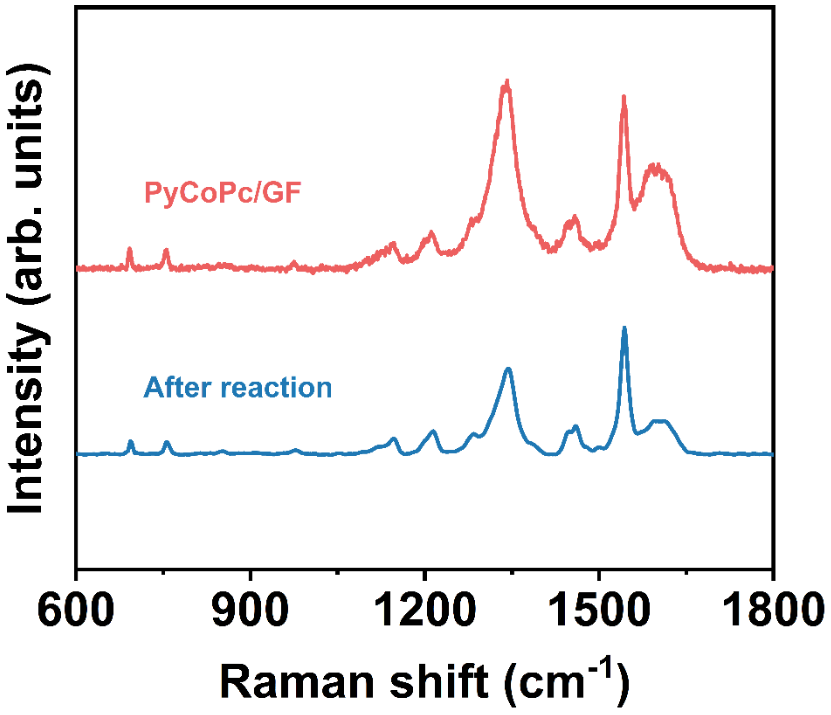

Supplementary Fig. 30 Raman spectra of PyCoPc/GF before and after reaction.

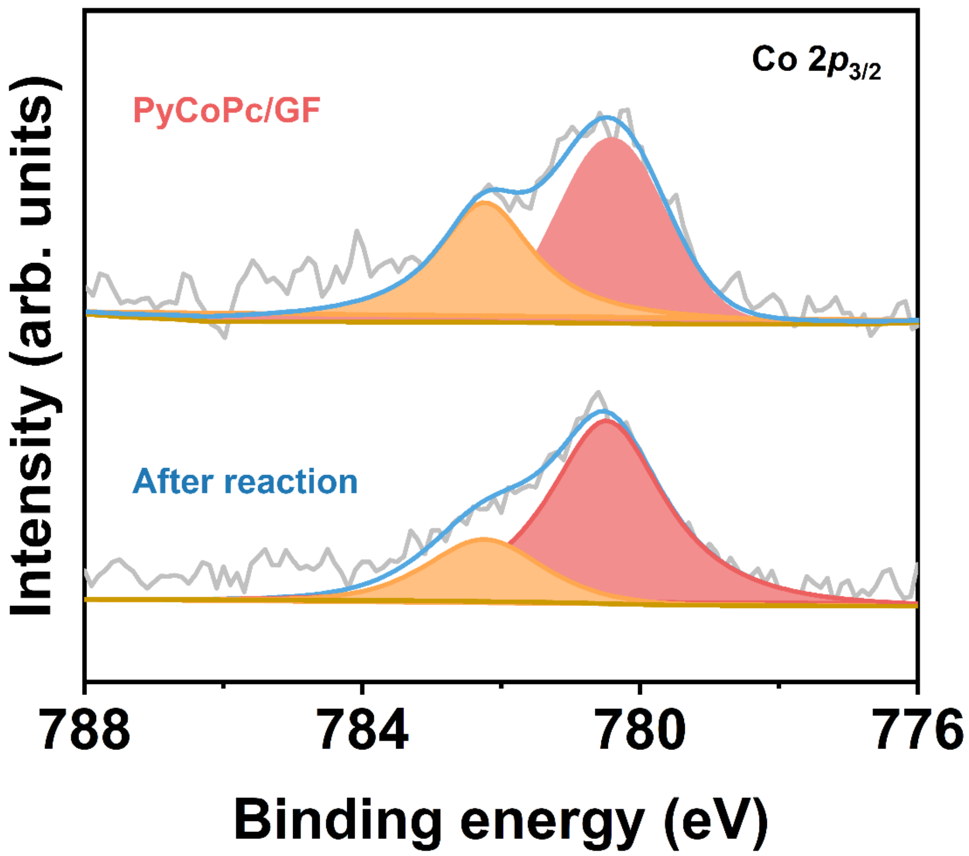

Supplementary Fig. 31 Co 2*p* XPS spectra of PyCoPc/GF before and after reaction.

After the reaction, the Co $2p_{3/2}$ XPS spectrum of PyCoPc/GF showed that the Co^{2+} peak was observed at 780.4 eV with no significant shift.

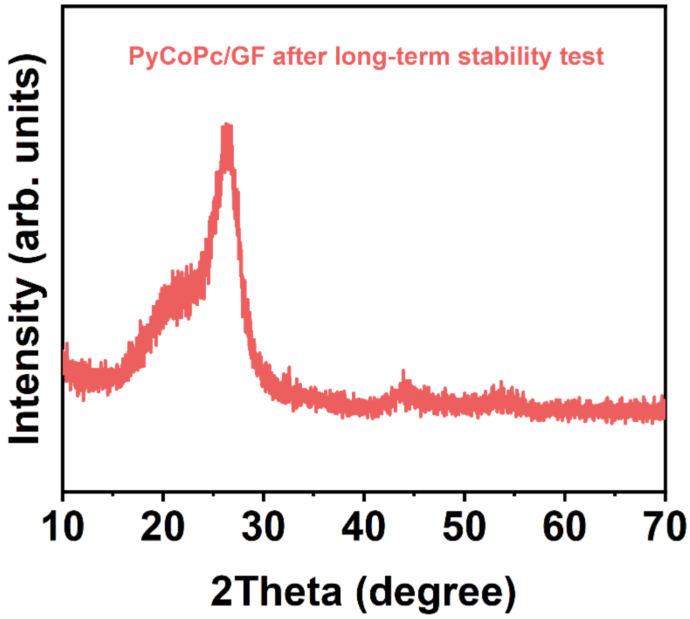

Supplementary Fig. 32 XRD pattern of PyCoPc/GF after long-term stability test.

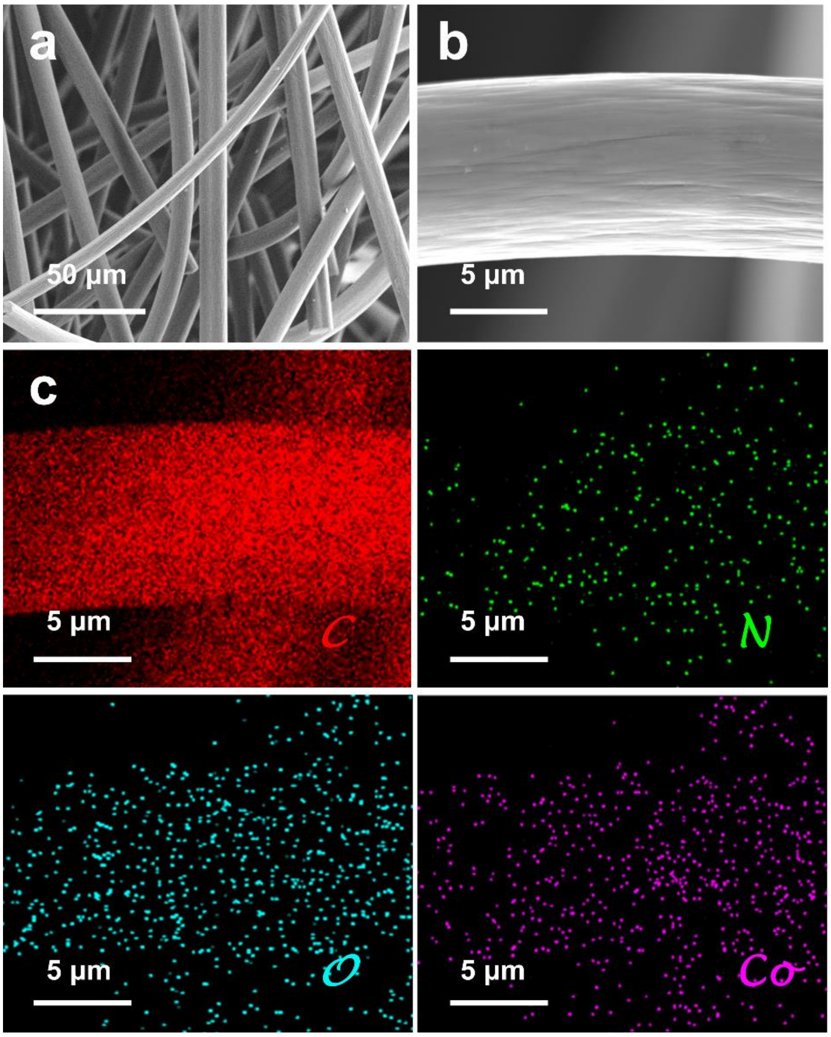

Supplementary Fig. 33 (a,b) SEM images and (c) corresponding EDX elemental mapping images of PyCoPc/GF after long-term stability test.

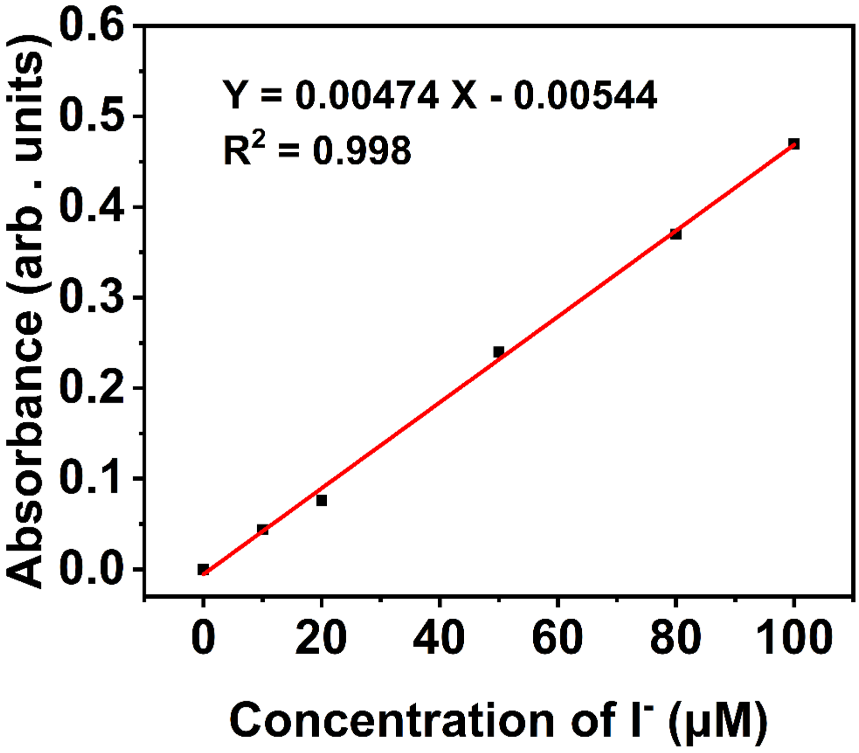

Supplementary Fig. 34 UV-Vis spectrophotometric calibration curve for I^- quantification.

During extended stability testing, the electrolyte was replenished every 10 h, maintaining the mediator concentration well above the reaction stoichiometry. Throughout the reaction, a portion of the generated I_2 participated in the reaction, while the excess I_2 exists in equilibrium with I^- in the electrolyte via the reaction $I_3^- \rightleftharpoons I_2 + I^-$. Both forms are considered part of the effective iodine content. After reaction, I_2 and I_3^- were quantitatively reduced to I^- using Na_2SO_3, and the I^- concentration was determined by UV-Vis spectrophotometry.

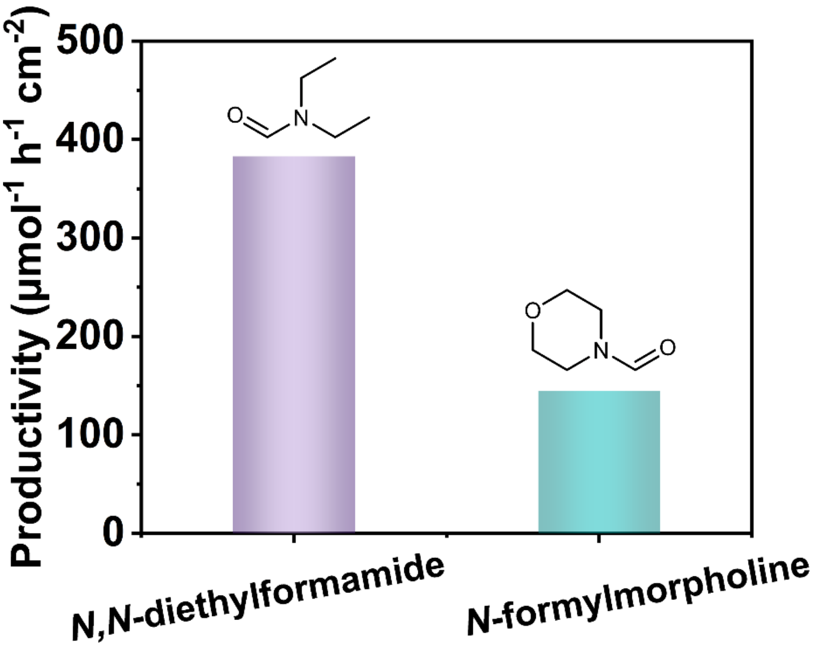

Supplementary Fig. 35 Substrate scope for the I_2-mediated strategy.

Under the optimal conditions for electrosynthesis in DMF, dimethylamine was replaced with equivalent volumes of 40 wt% diethylamine and morpholine solutions.

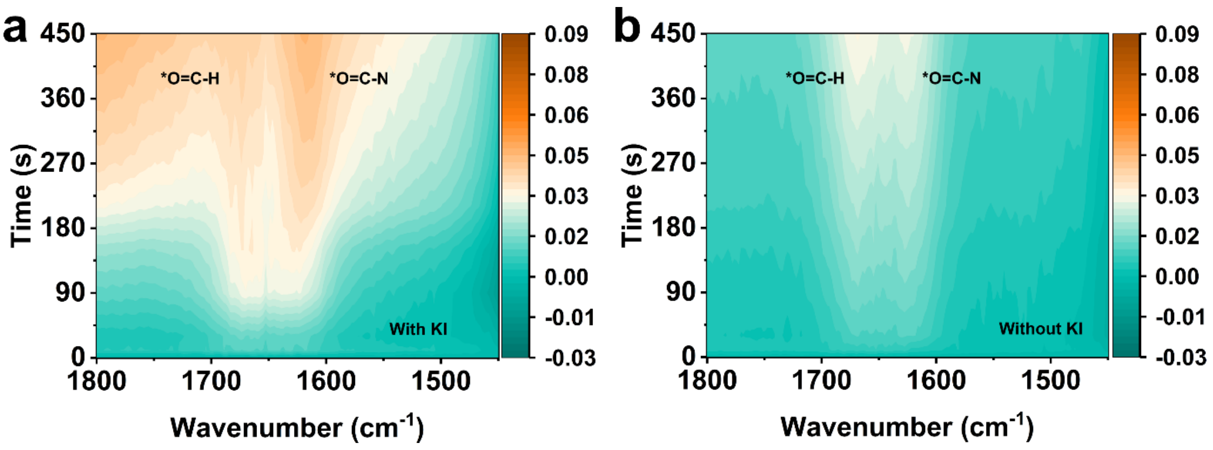

Supplementary Fig. 36 In situ ATR-IR spectra for the DMF electrosynthesis over PyCoPc/GF in 0.7 M K_2CO_3 (a) with 100 mM KI and (b) without KI.

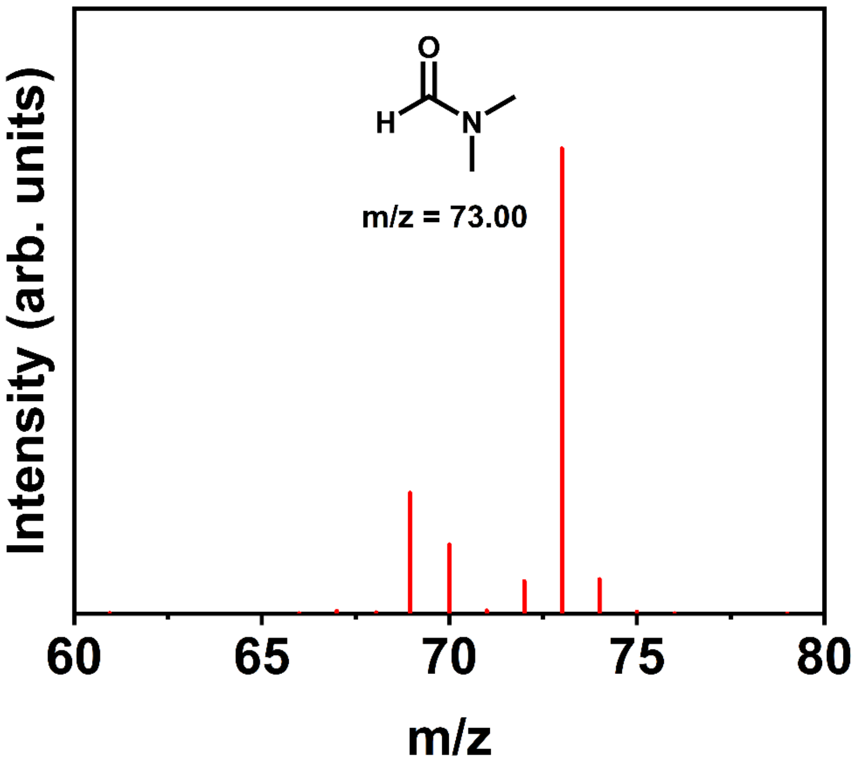

Supplementary Fig. 37 GC-MS spectrum of DMF using ^{13}C-labeled K_2CO_3.

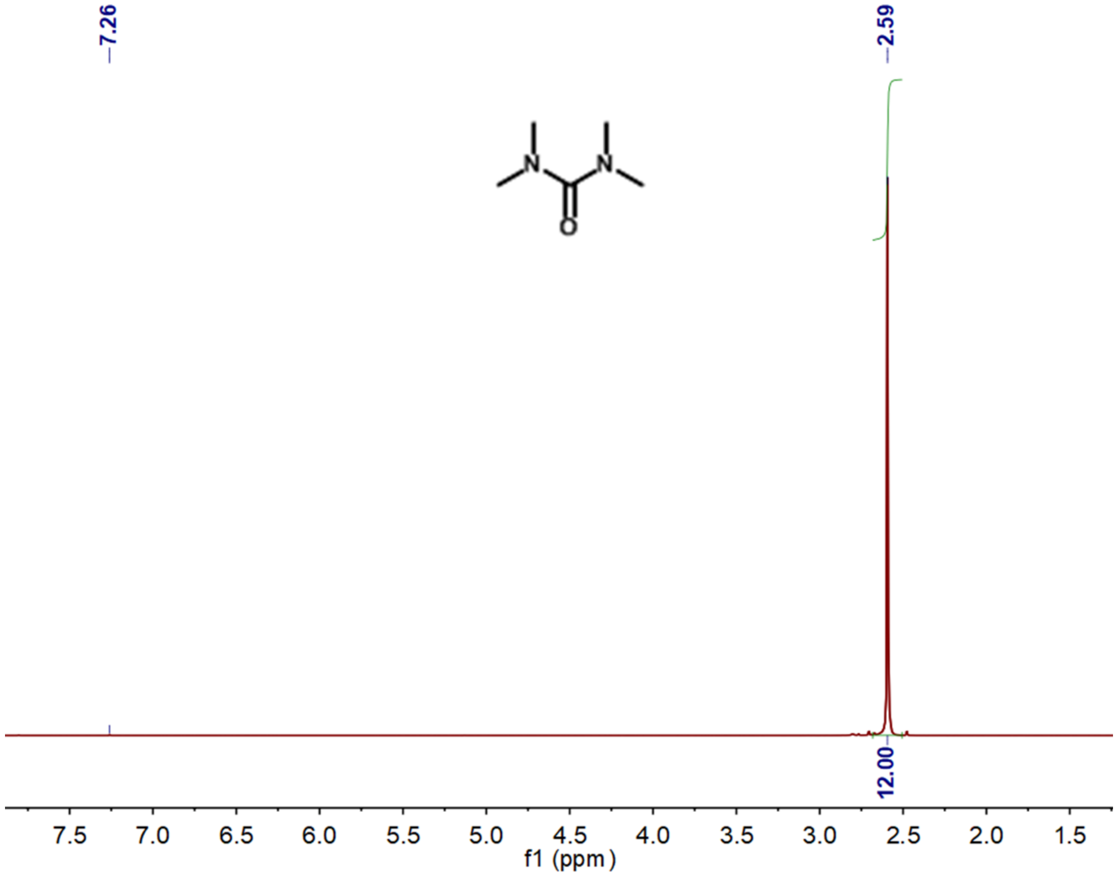

Supplementary Fig. 38 ^{1}H NMR spectrum of tetramethylurea.

^{1}H NMR (600 MHz, $CDCl_3$): δ (ppm) 2.59 (s, 12H).

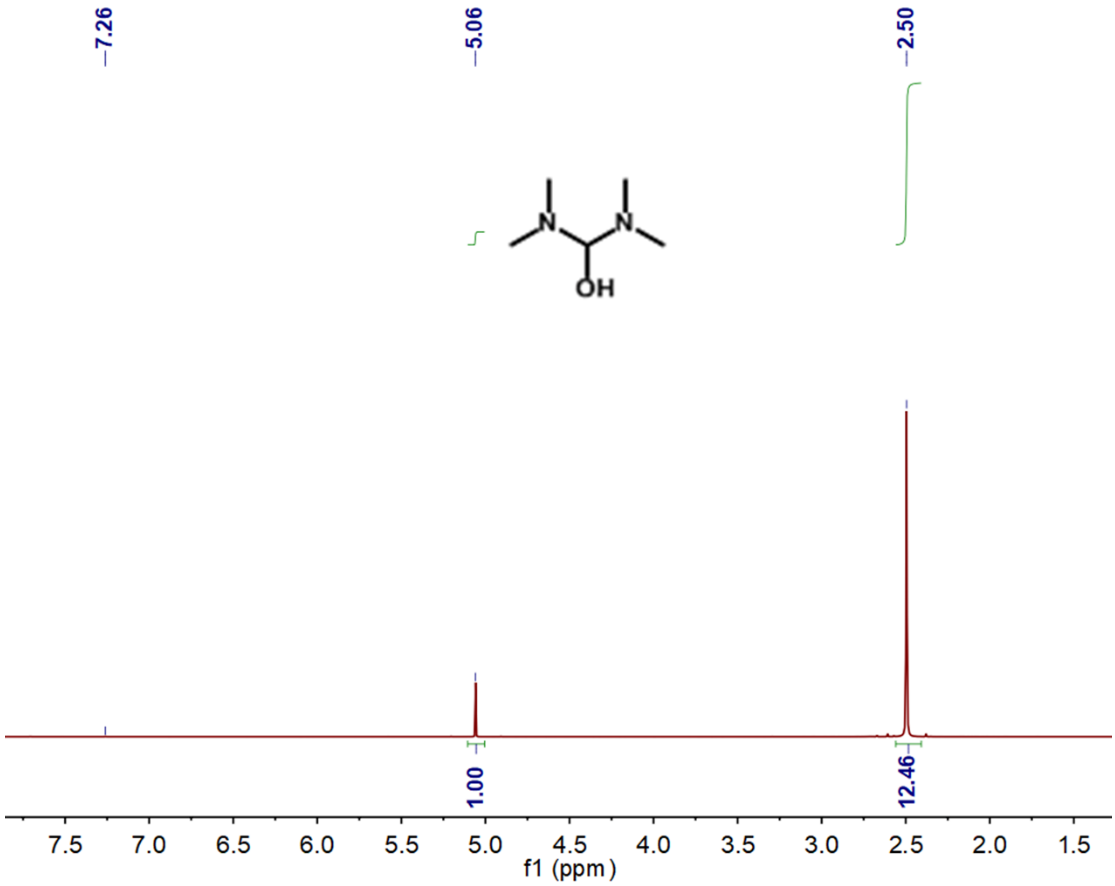

Supplementary Fig. 39 ^{1}H NMR spectrum of bis(dimethylamino)methanol.

^{1}H NMR (600 MHz, $CDCl_3$): δ (ppm) 5.06 (s, 1H), 2.50 (s, 12H).

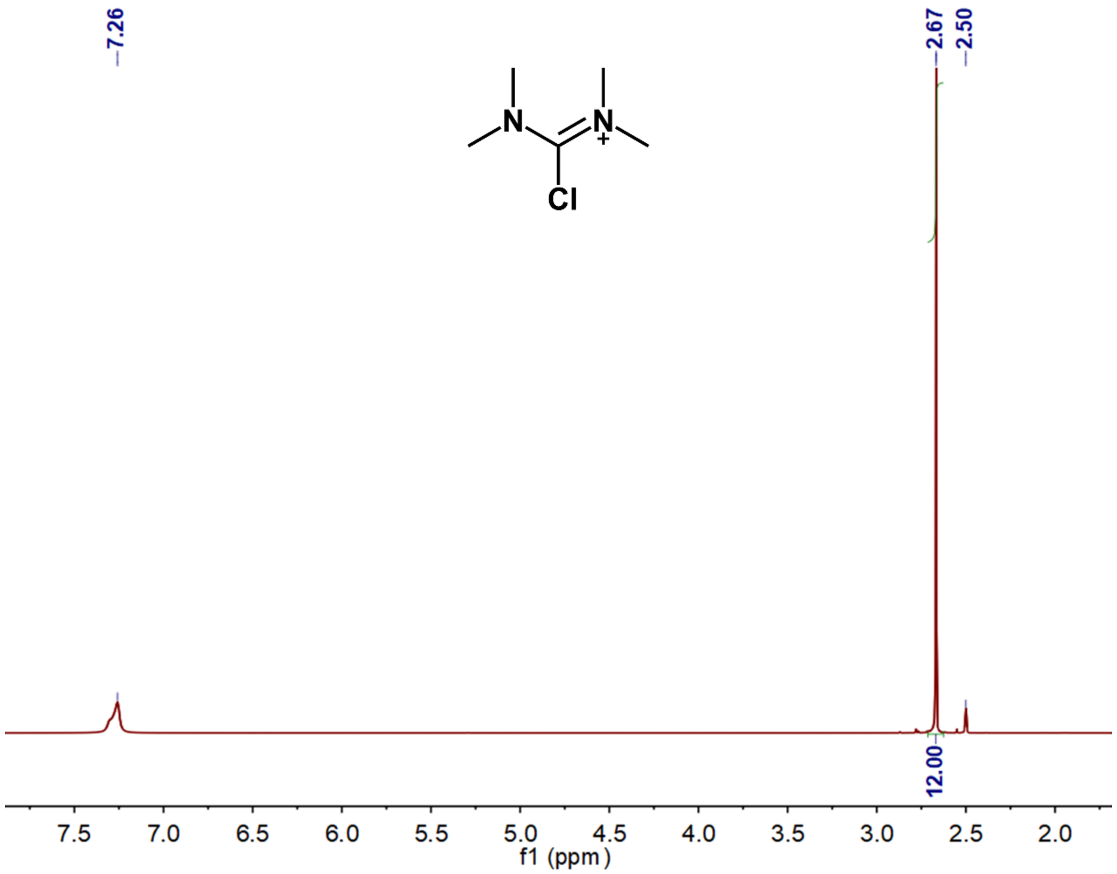

Supplementary Fig. 40 ^{1}H NMR spectrum of tetramethyl-chloroformamidinium chloride.

^{1}H NMR (600 MHz, $CDCl_3$): δ (ppm) 2.67 (s, 12H).

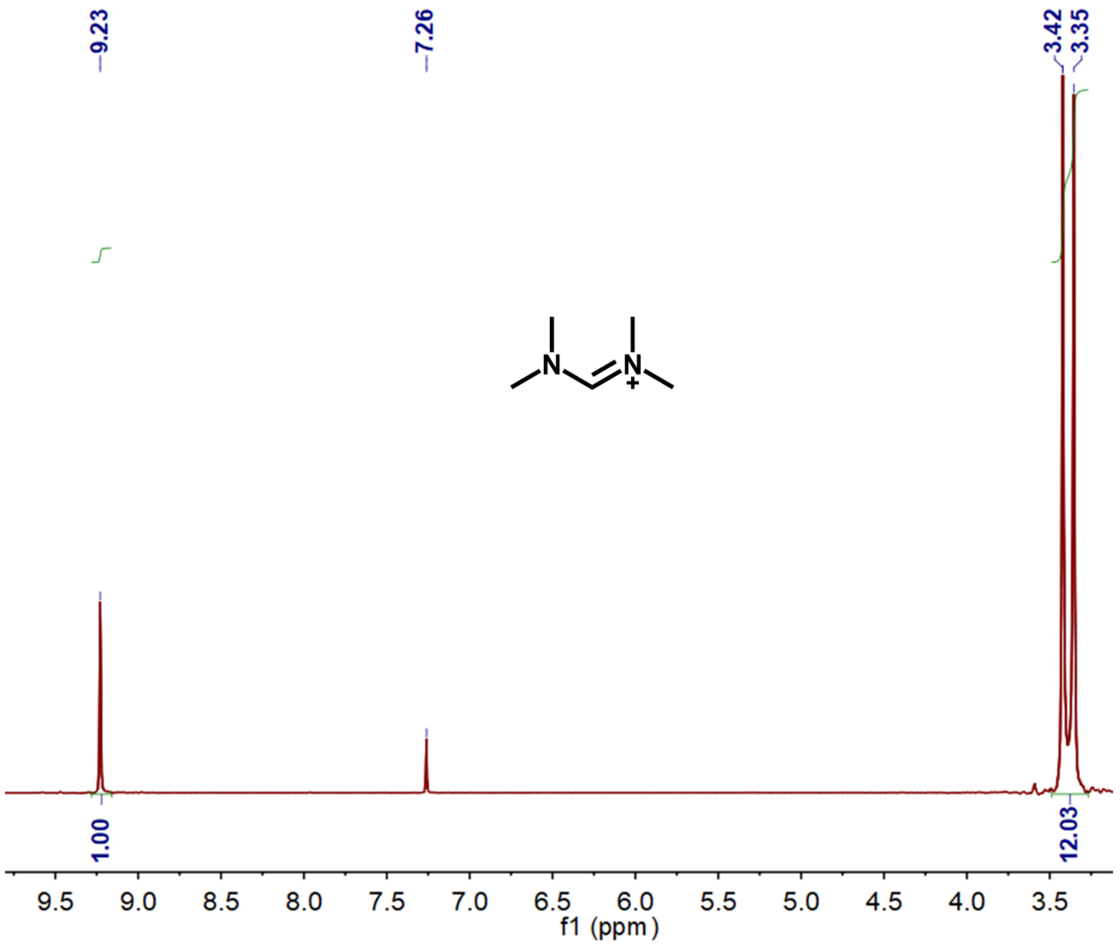

Supplementary Fig. 41 ^{1}H NMR spectrum of (dimethylaminomethylene)dimethylammonium chloride.

^{1}H NMR (600 MHz, $CDCl_3$): δ (ppm) 9.23 (s, 1H), 3.35 (d, 12H).

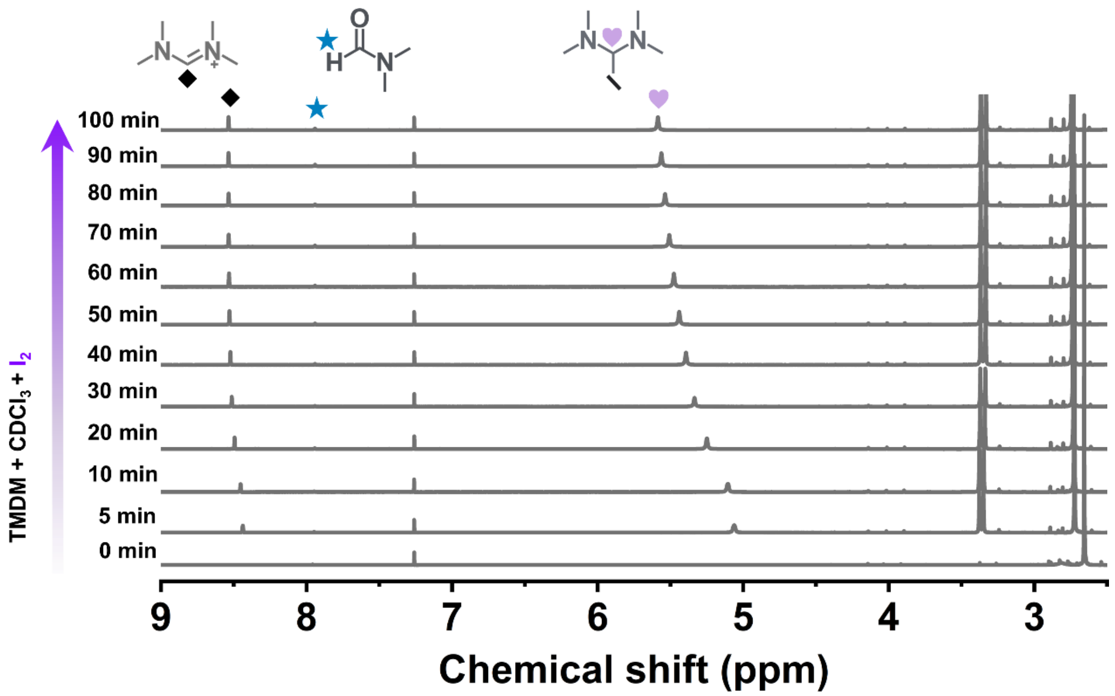

Supplementary Fig. 42 Quasi-in situ ^{1}H NMR spectra of TMDM in $CDCl_3$ with 10 mg I_2.

As shown in Supplementary Fig. 42 and Fig. 4c, I_2 rapidly reacts with TMDM to generate the highly reactive iodinated intermediate, 1-iodo-*N,N,N′,N′*-terametheylmethanediamine. As the reaction progresses, the integrated intensity of the C–H proton signal for this intermediate gradually increases and its chemical shift moves downfield, eventually reaching a steady state. This downfield shift is attributed to reduced electron density around the C–H bond due to strong electronic interactions with I_2 and the solvent, resulting in deshielding. Notably, the iminium cation exhibits a distinct upfield shift to 7.49 ppm in 0.7 M K_2CO_3 (Fig. 4d), compared to 8.53 ppm in $CDCl_3$ (Fig. 4c) This contrast further illustrates the complex influence of the solvent environment on electronic structure and shielding effects.

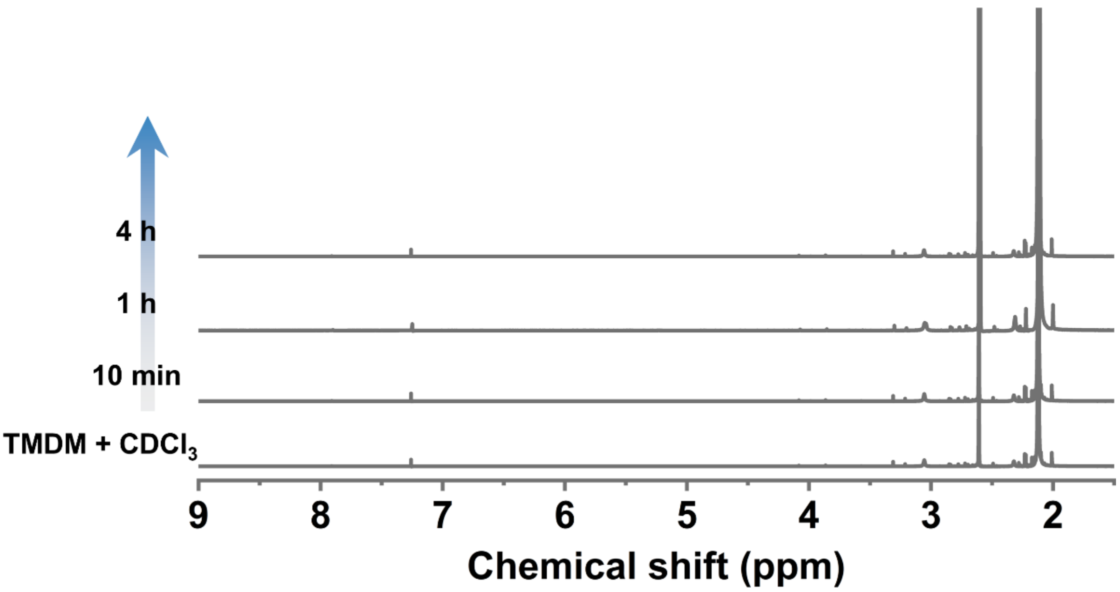

Supplementary Fig. 43 Quasi-in situ ^{1}H NMR spectra of TMDM in $CDCl_3$.

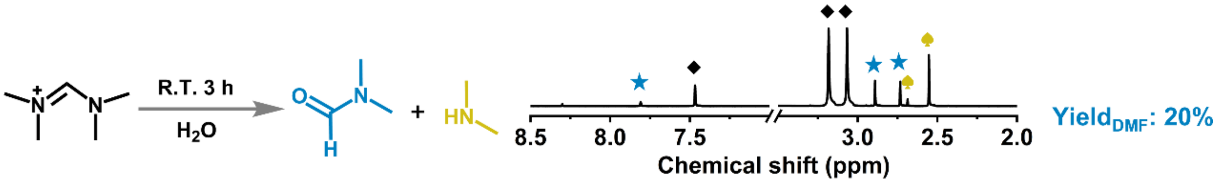

Supplementary Fig. 44 Yield analysis of iminium ion intermediate conversion to DMF in pure H_2O.

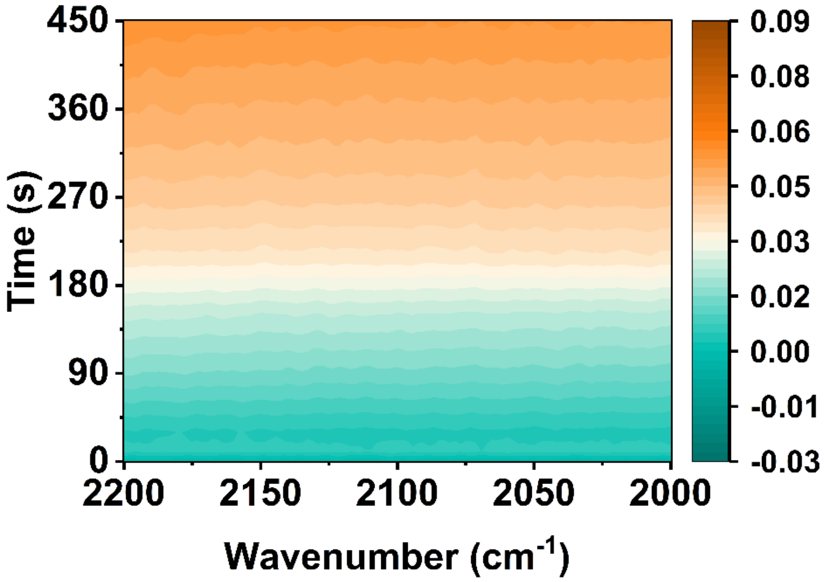

Supplementary Fig. 45 Magnified view of in situ ATR-IR spectrum for the DMF electrosynthesis over PyCoPc/GF in 0.7 M K_2CO_3 with 100 mM KI.

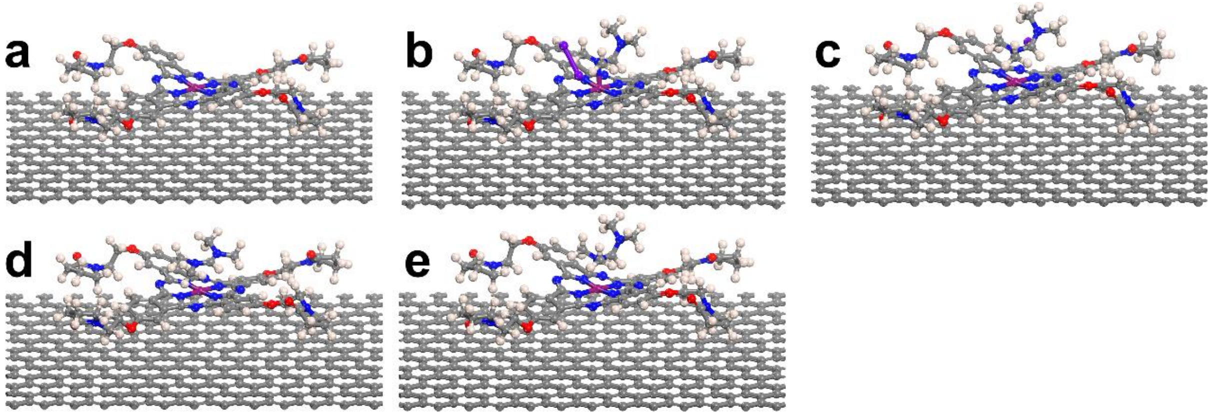

Supplementary Fig. 46 DFT models of (a) PyCoPc/GF; (b) ad-($(CH_3)_2NCH_2N(CH_3)_2$) in the presence of I_2; (c) ad-($(CH_3)_2NCHIN(CH_3)_2$); (d) ad-($(CH_3)_2NCH{=}N^+(CH_3)_2$); (e) ad-($(CH_3)_2NCH_2N(CH_3)_2$) without I_2.

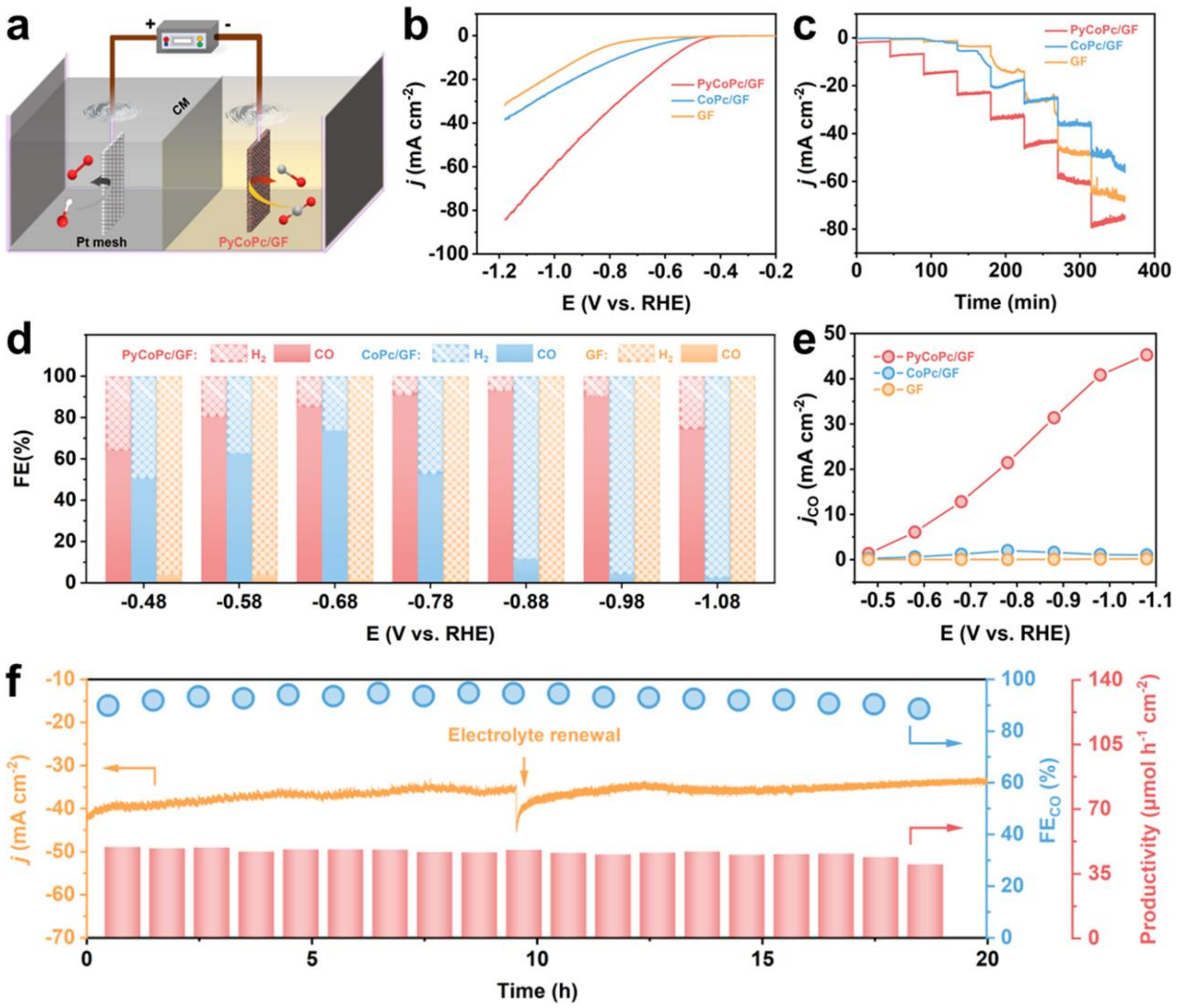

Supplementary Fig. 47 ECR performance of PyCoPc/GF, CoPc/GF and GF: (a) schematic diagram of electrolytic cell; (b) LSV curves; (c) i-t curves; (d) FEs; (e) j_{CO}; (f) stability test.

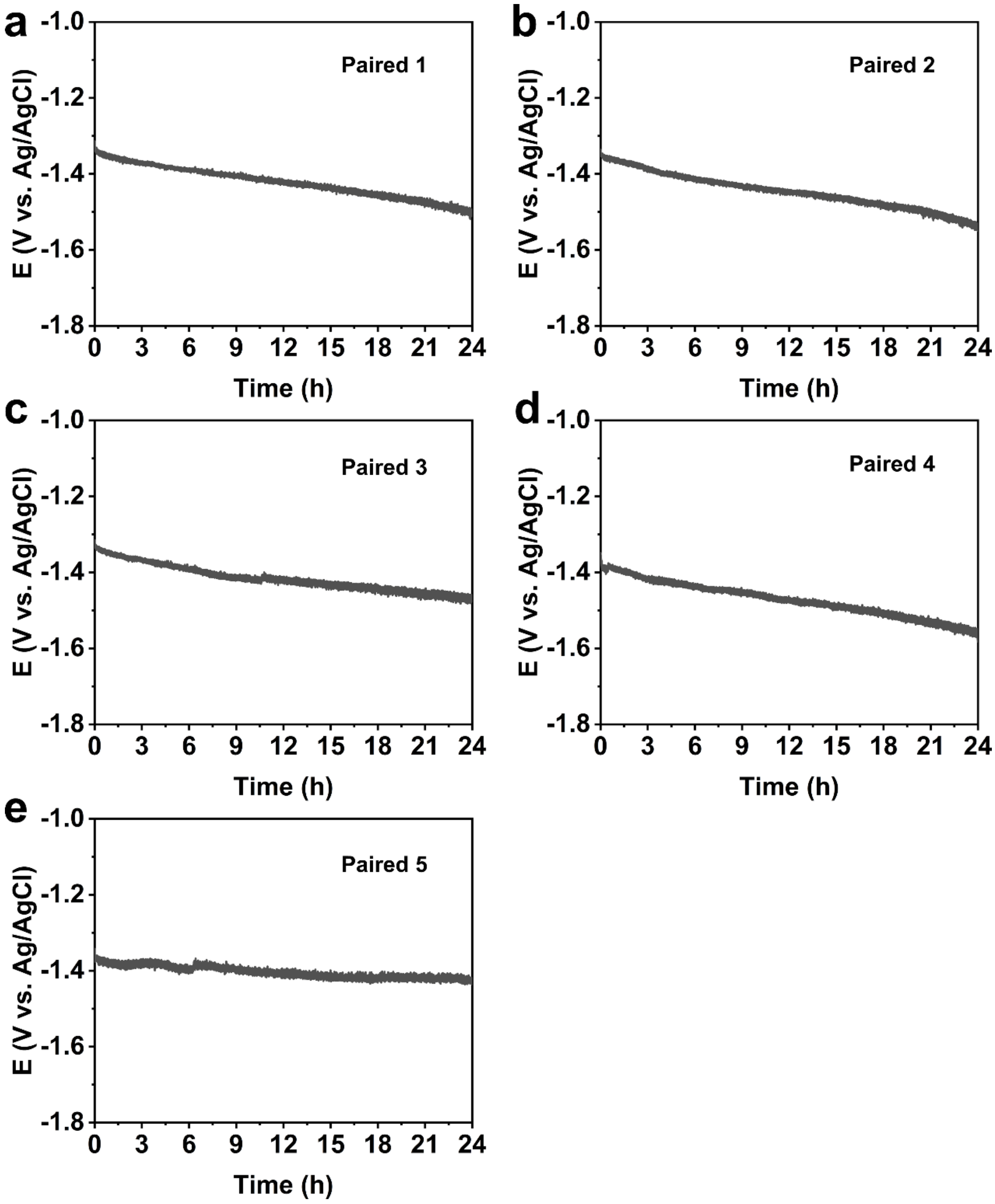

Supplementary Fig. 48 I-t curves of (a) paired 1; (b) paired 2; (c) paired 3; (d) paired 4; (e) paired 5.

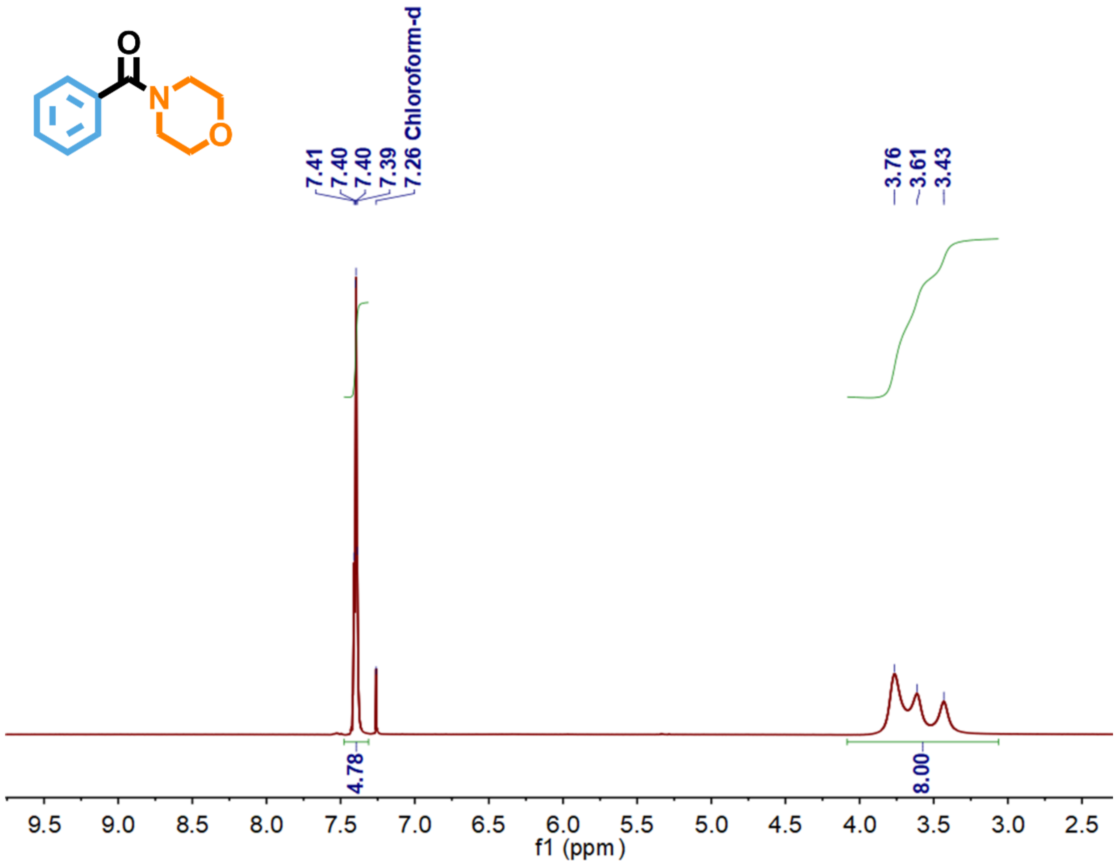

Supplementary Fig. 49 ^{1}H NMR spectrum of **A1**.

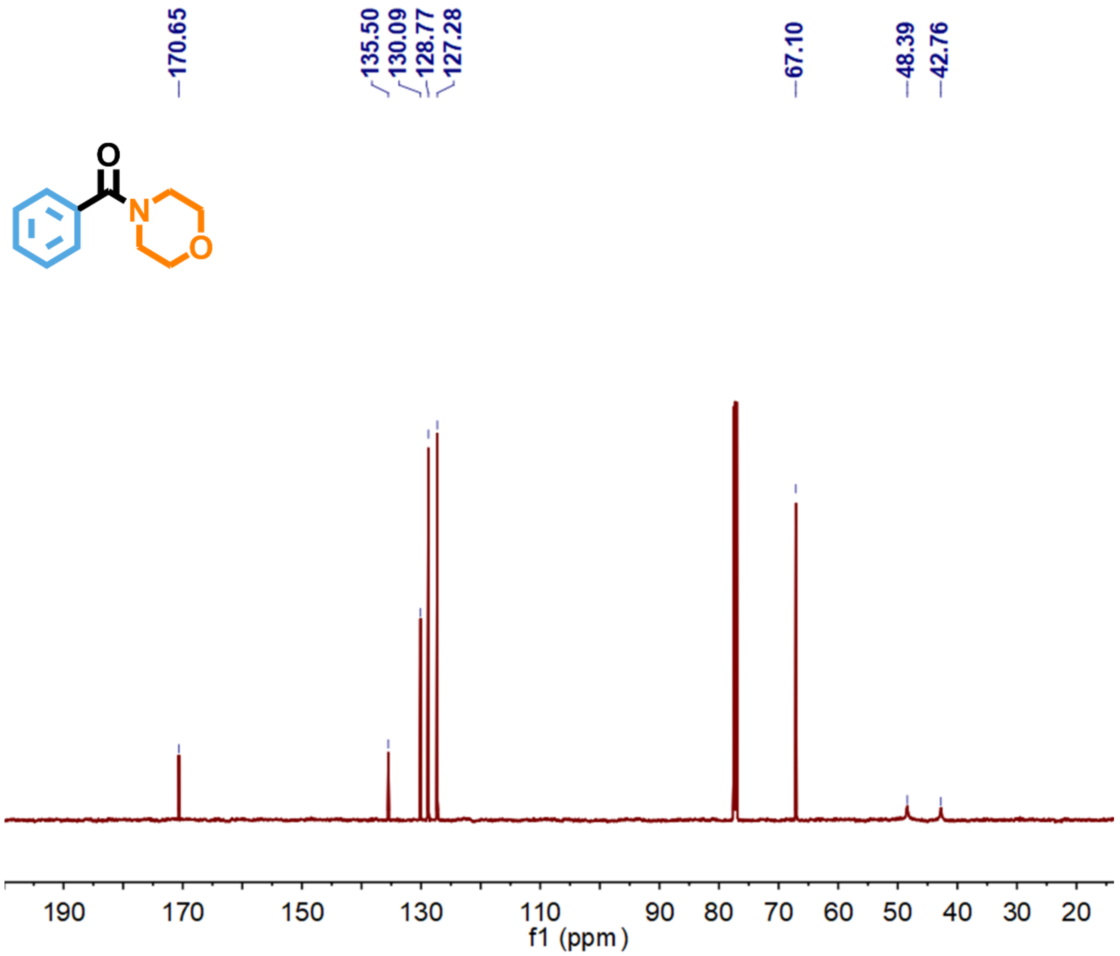

Supplementary Fig. 50 ^{13}C NMR spectrum of **A1**.

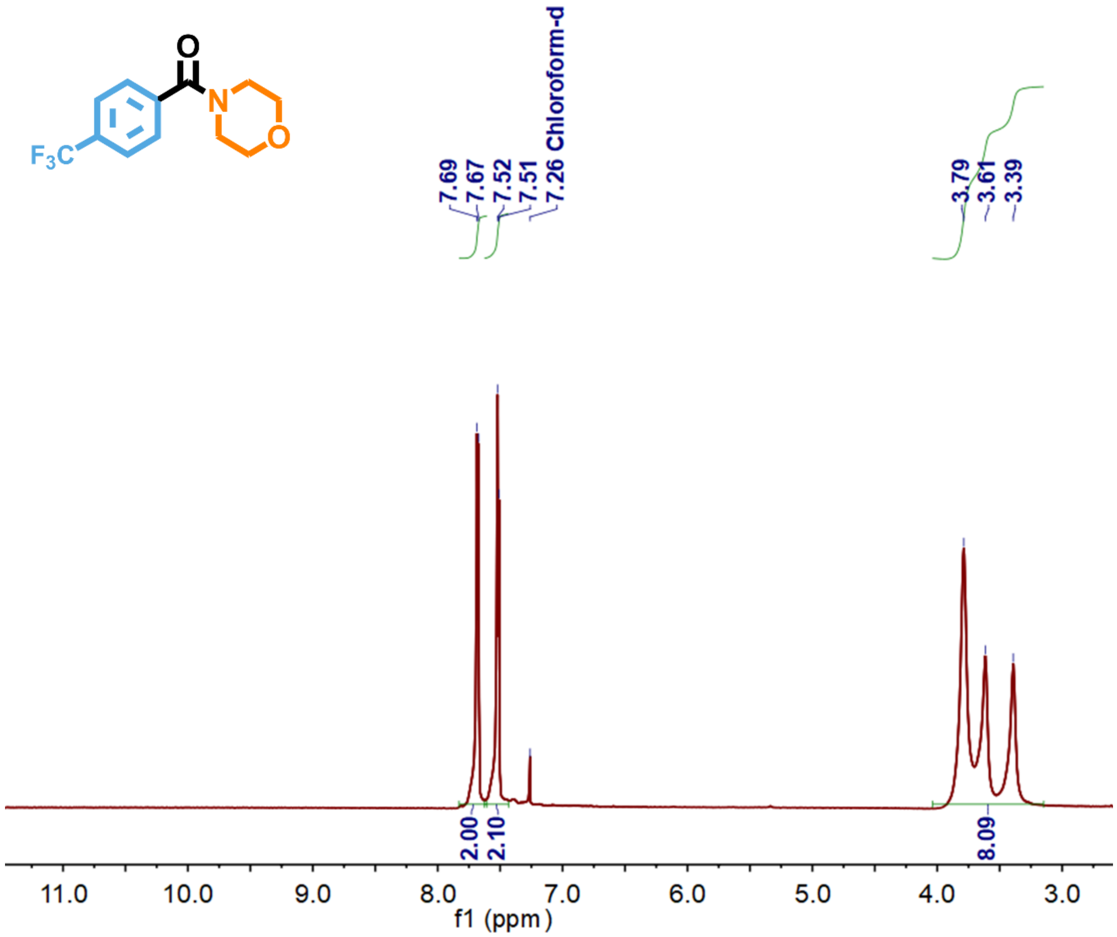

Supplementary Fig. 51 ^{1}H NMR spectrum of **A2**.

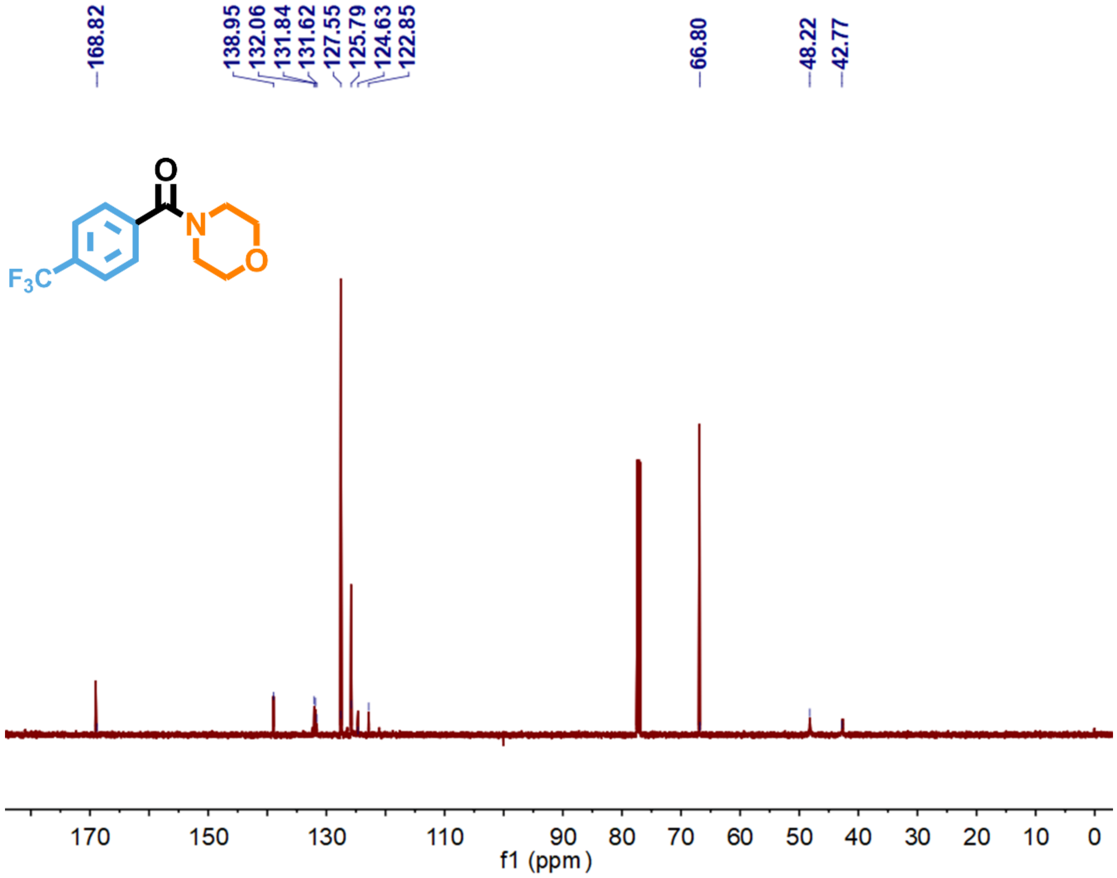

Supplementary Fig. 52 ^{13}C NMR spectrum of **A2**.

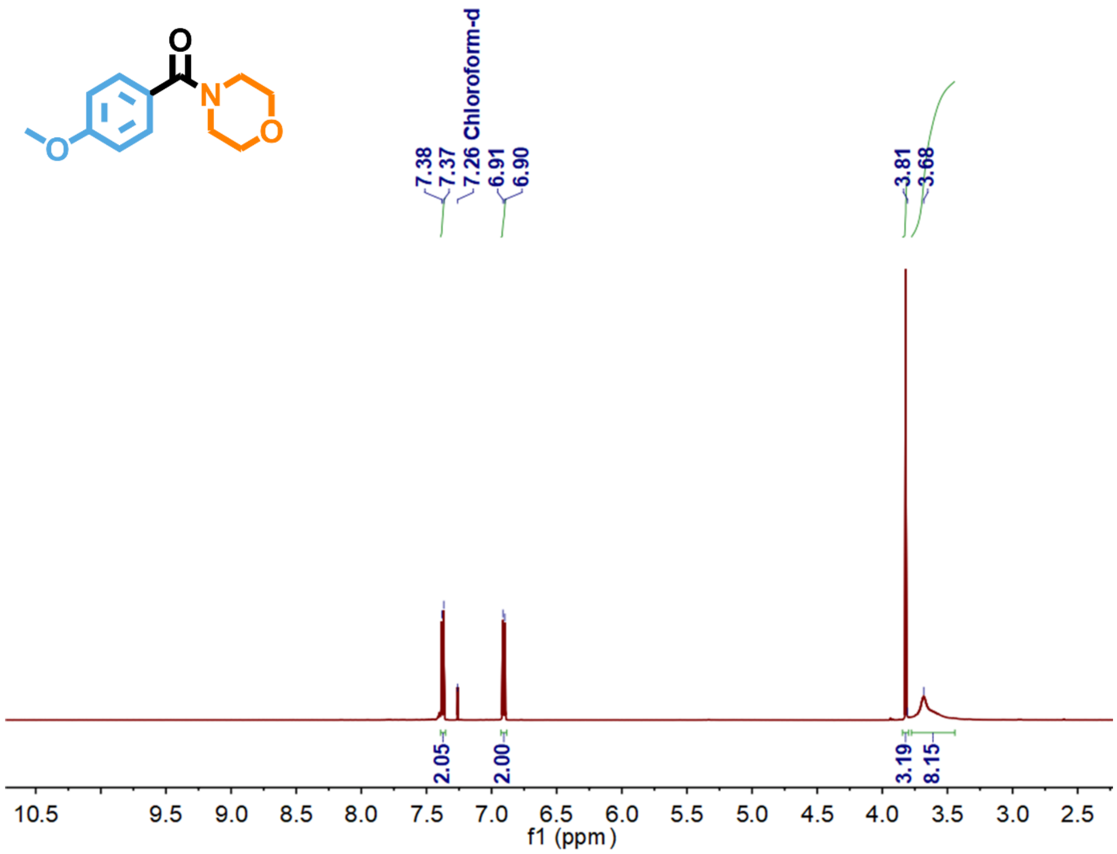

Supplementary Fig. 53 ^{1}H NMR spectrum of **A3**.

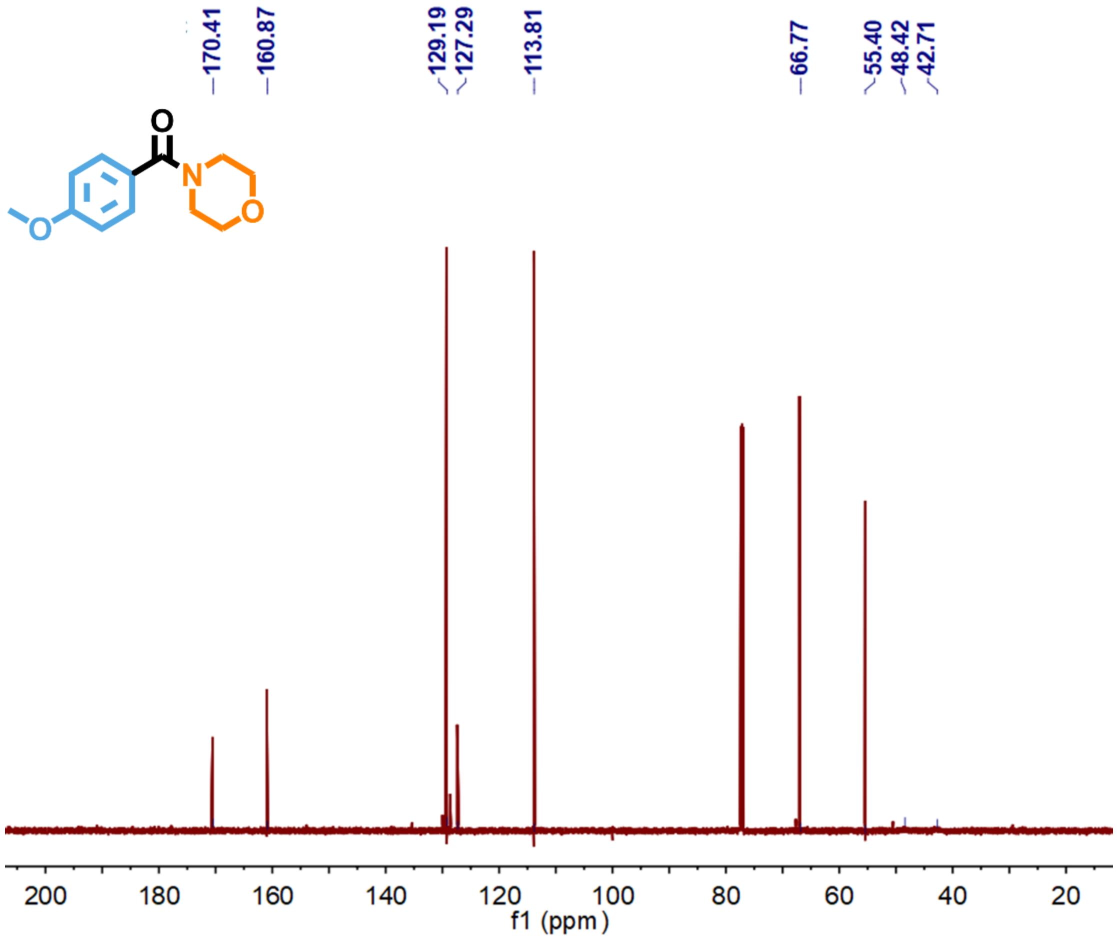

Supplementary Fig. 54 ^{13}C NMR spectrum of **A3**.

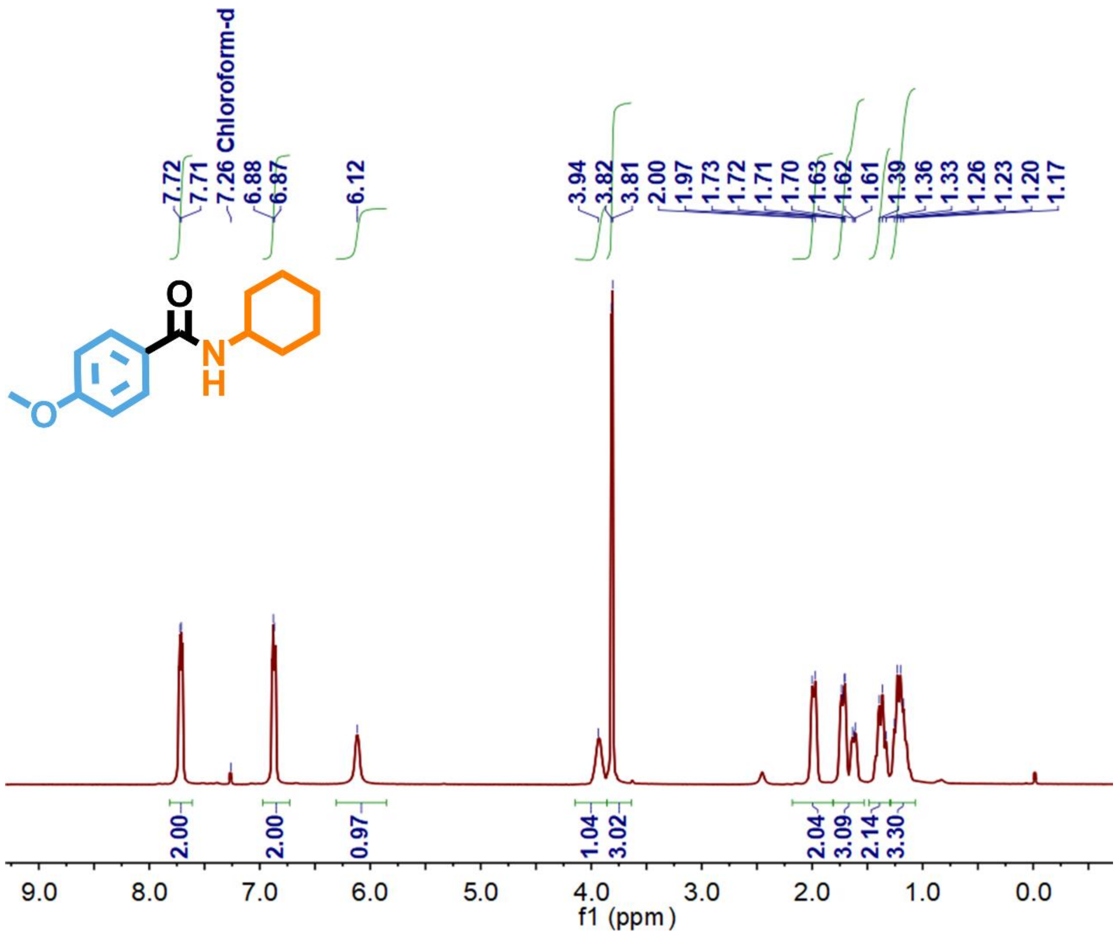

Supplementary Fig. 55 ^{1}H NMR spectrum of **A4**.

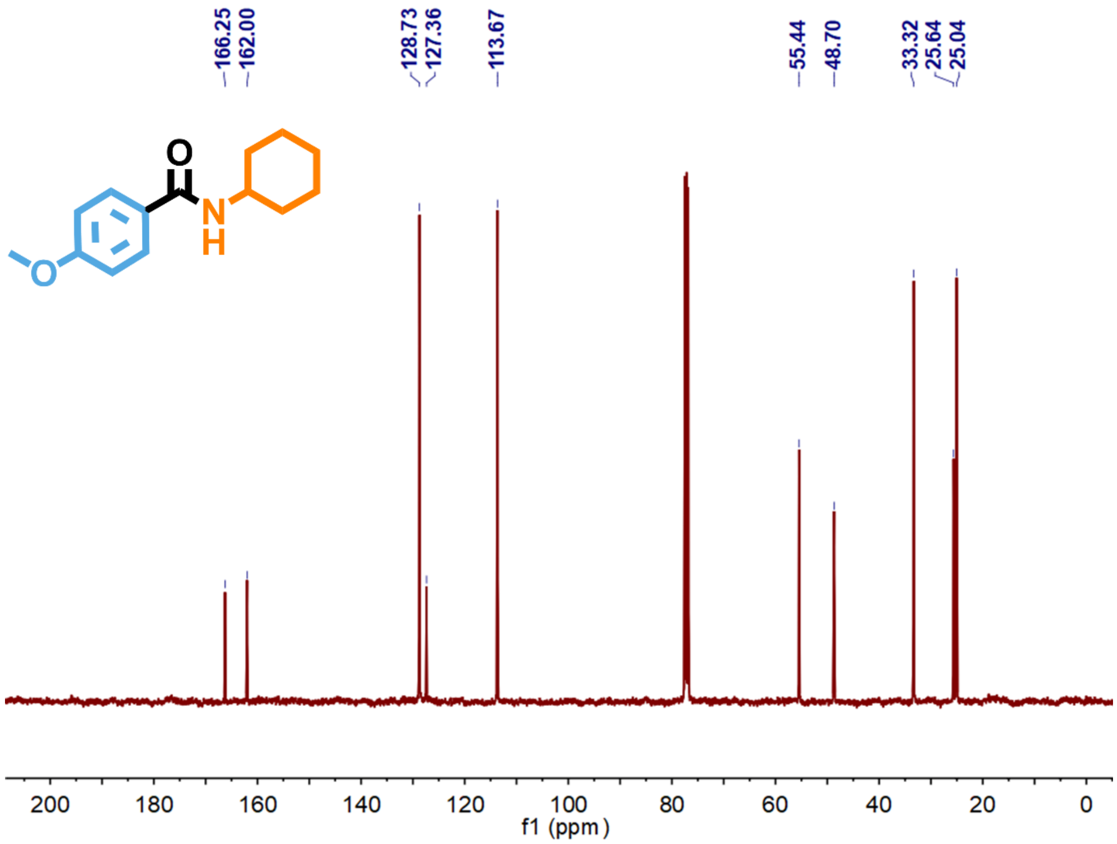

Supplementary Fig. 56 ^{13}C NMR spectrum of **A4**.

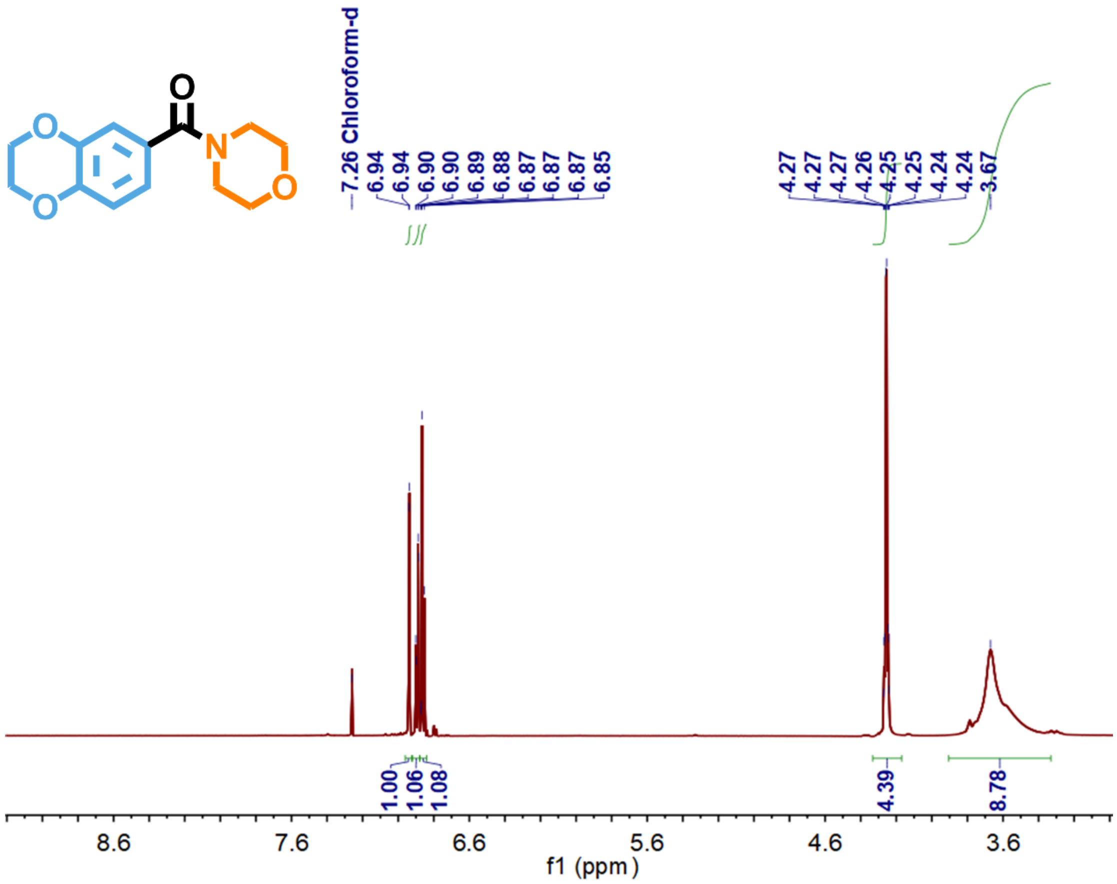

Supplementary Fig. 57 ^{1}H NMR spectrum of **A5**.

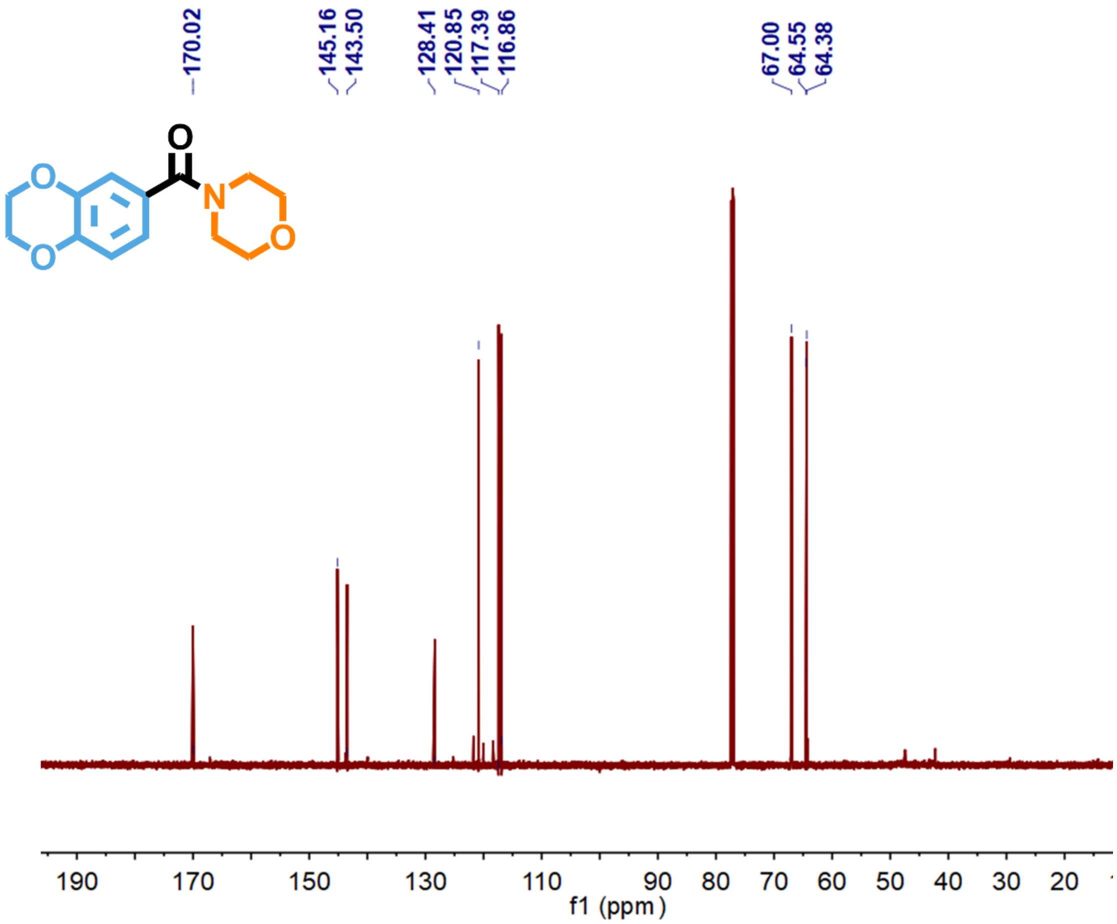

Supplementary Fig. 58 ^{13}C NMR spectrum of **A5**.

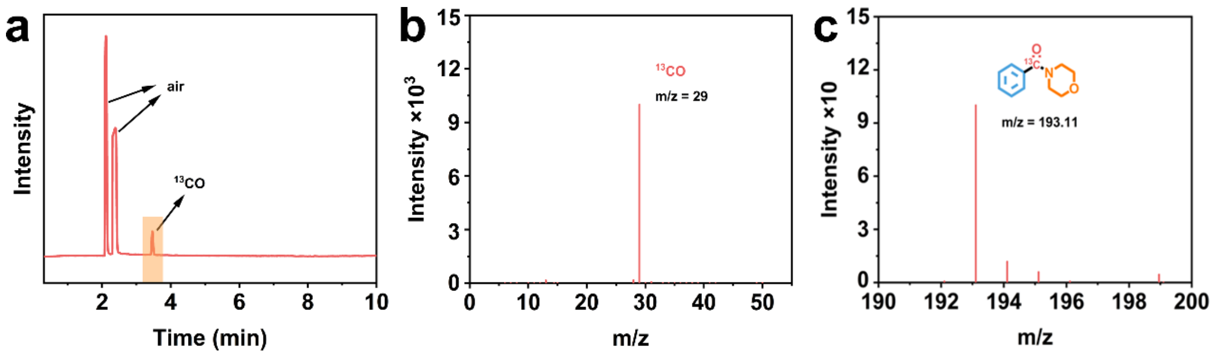

Supplementary Fig. 59 (a) ^{13}C labeled GC spectrometry based on $^{13}CO_2$-to-^{13}CO electroconversion; GC-MS of synthesized (b) ^{13}CO and (c) ^{13}C labeled **A1**.

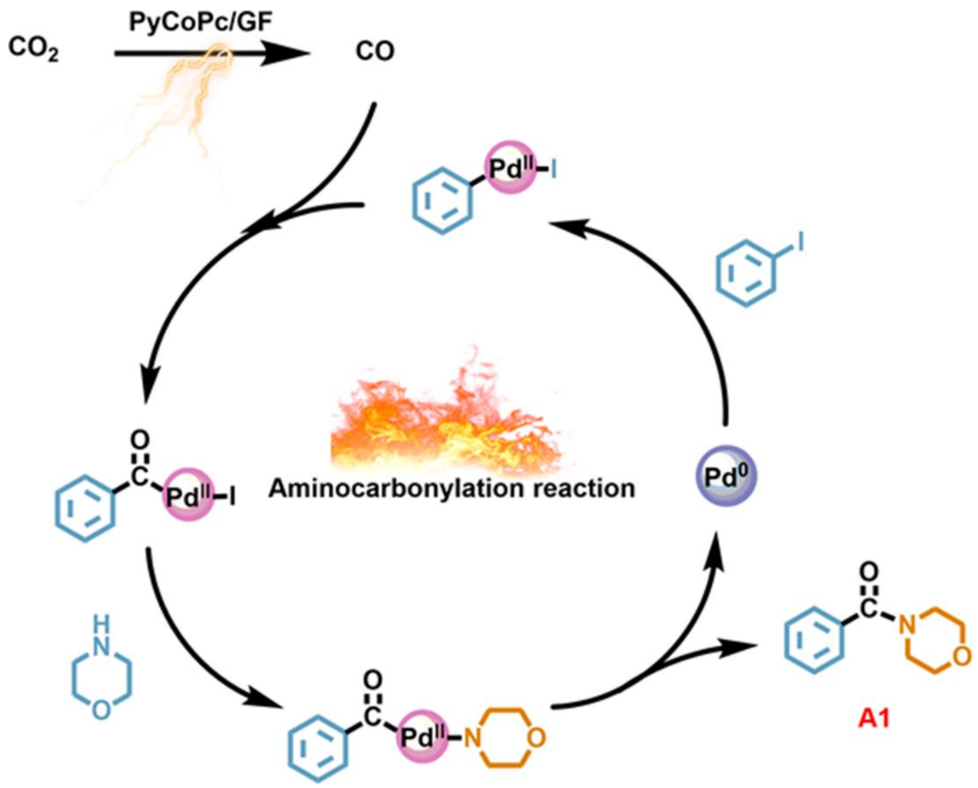

Supplementary Fig. 60 Proposed mechanism for the cathodic tandem.

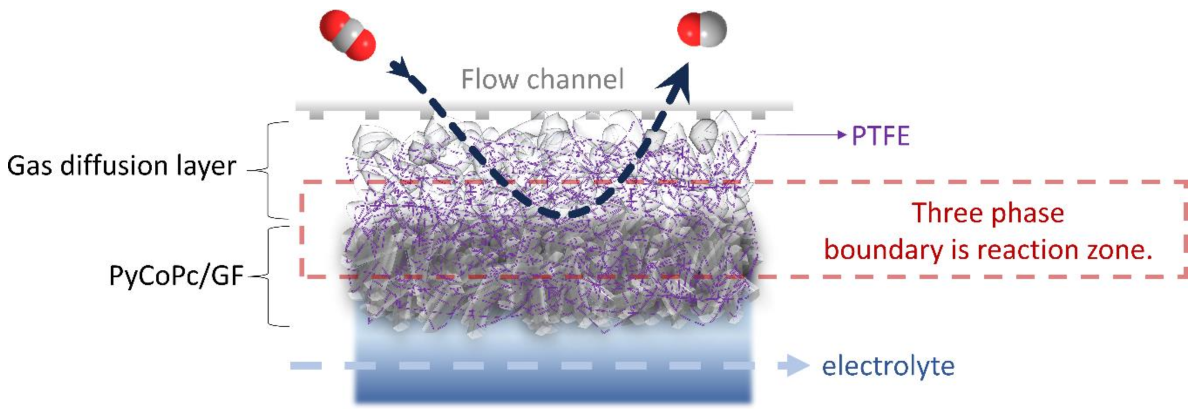

Supplementary Fig. 61 Schematic of the GDE configuration.

Fabrication of a gas diffusion electrode (GDE) established a stable CO_2-catalyst-electrolyte three-phase interface, significantly enhancing CO_2 mass transport efficiency. Direct CO_2 supply to the electrode facilitated replacement of the catholyte (0.5 M $KHCO_3$) in the H-type cell with 1 M KOH, avoiding bulk dissolution. This substitution reduces the cathodic overpotential, thereby lowering the full-cell voltage and improving the performance of both electrodes.

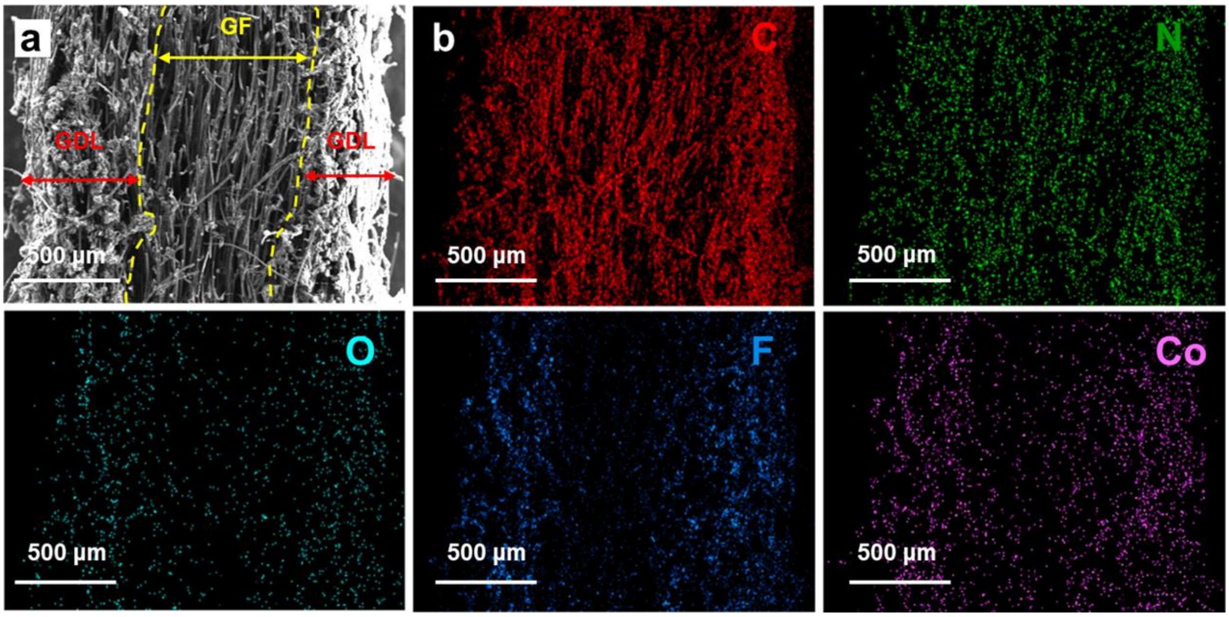

Supplementary Fig. 62 Cross-sectional views of PyCoPc/GF-GDE: (a) SEM and (b) EDX elemental mapping images.

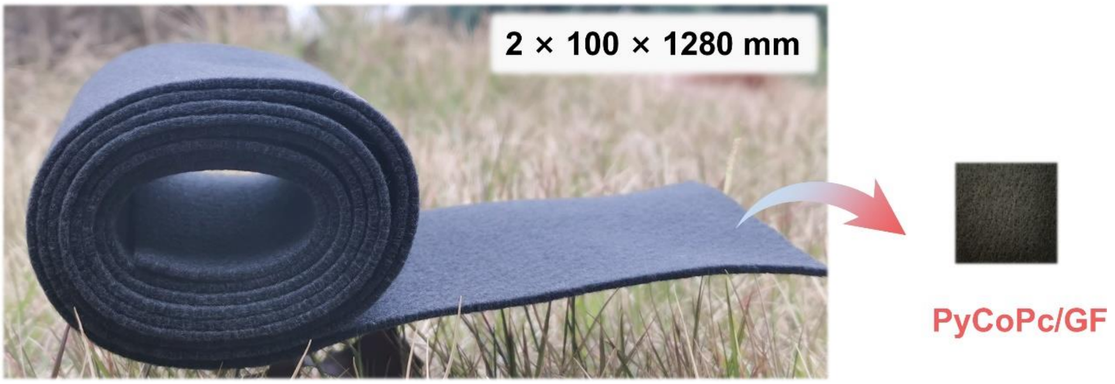

Supplementary Fig. 63 Large-size preparation of PyCoPc/GF electrode with dimensions of 2 mm × 100 mm × 1280 mm.

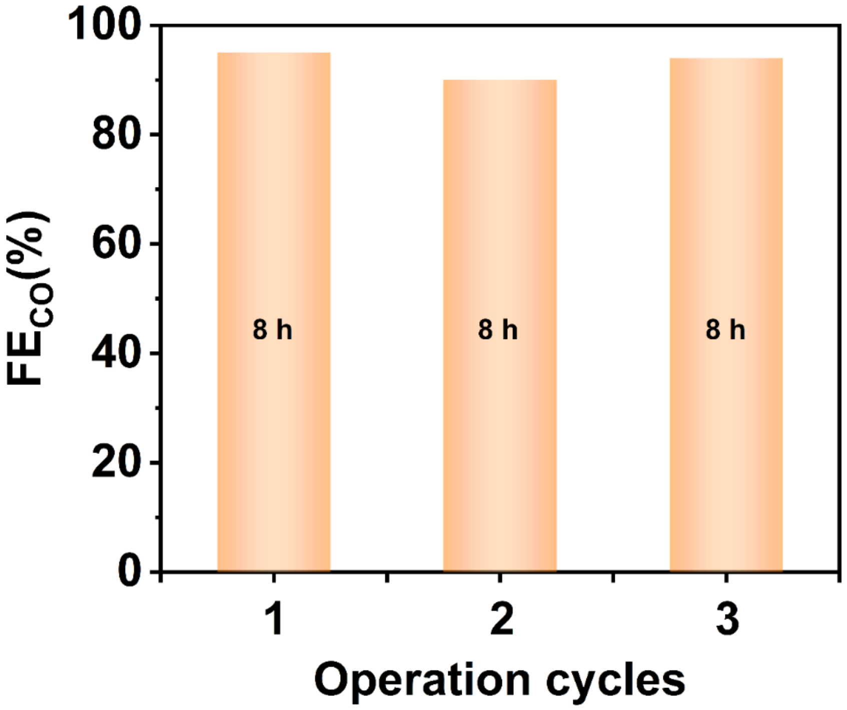

Supplementary Fig. 64 Performance of cathodic ECR in a flow cell.

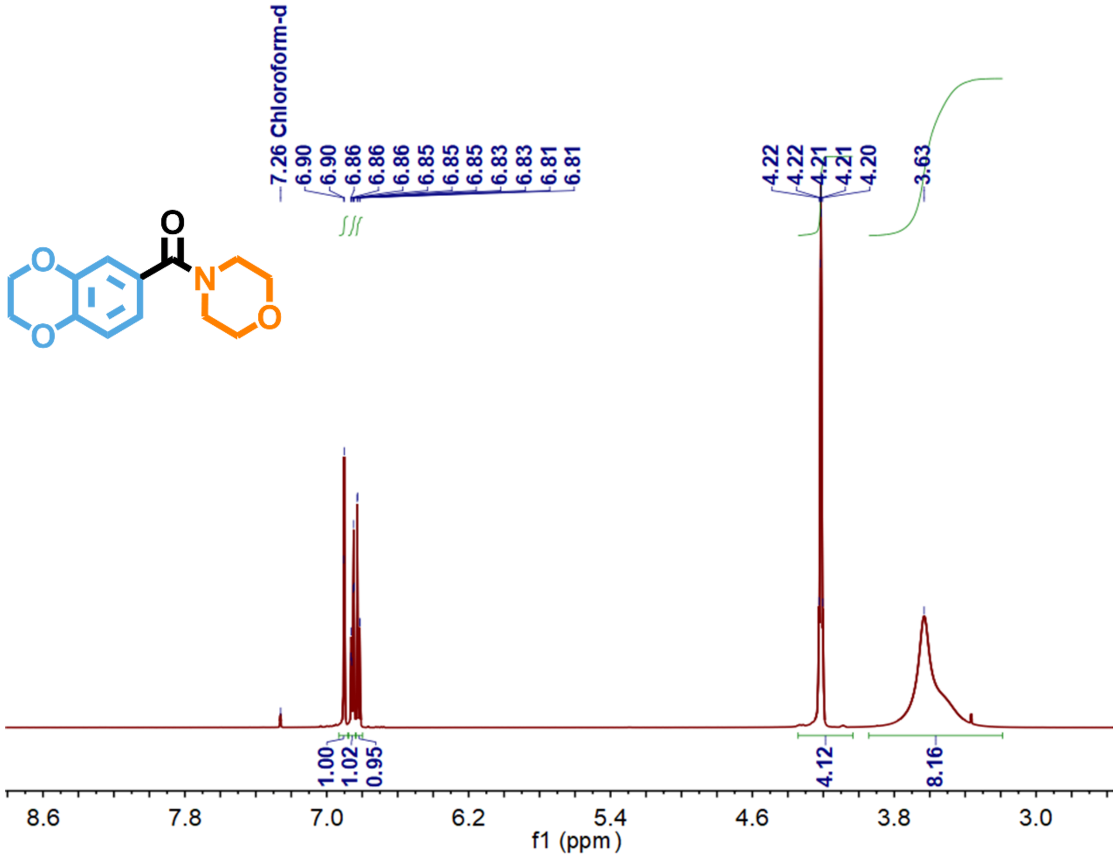

Supplementary Fig. 65 ^{1}H NMR spectrum of **A5** prepared in the flow cell.

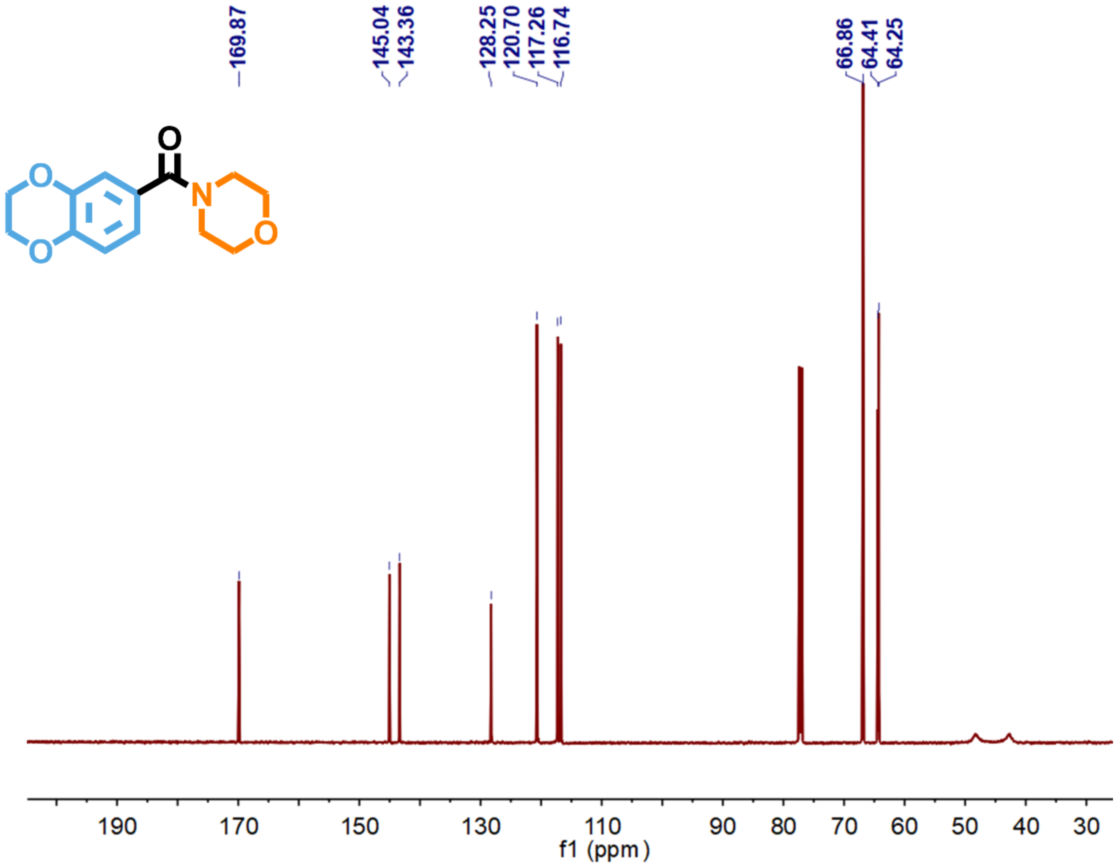

Supplementary Fig. 66 ^{13}C NMR spectrum of **A5** prepared in the flow cell.

Supplementary Tables

Supplementary Table 1 Performance comparison of PyCoPc/GF and other reported electrocatalysts for the electrosynthesis of DMF.

Product	Electrocatalyst	Electrolyte	Reactants	FE(%)	Productivity (μmol h^{-1} cm^{-2})	Ref.
Anode	PyCoPc/GF	0.7 M K_2CO_3 + 100 mM KI	methanol and dimethylamine	~91	~850 (H-type cell)	This work
				~74	~2753 (flow cell)	This work
	OMS-Co/NC	0.7 M K_2CO_3	methanol and dimethylamine	~44	~221 (H-type cell)	7
	Graphite flake	0.7 M K_2CO_3	trimethylamine	~40	~6.25 (MEA)	8
	WO_2–NiOOH/Ni	0.5 M $KHCO_3$	methanol and dimethylamine	~50	~438 (single cell)	9
Cathode	Pd/Cu-V_{Cu}	0.5 M $KHCO_3$	CO_2 and dimethylamine	~37	~192 (flow cell)	10
	Ag/Cu	0.1 M $KHCO_3$ + 0.02 M KNO_3	CO_2 and NO_3^-	~0.32	~0.009 (flow cell)	11
	InN_3	0.1 M $KHCO_3$	CO_2 and dimethylamine	~22	~0.248 (H-type cell)	12

MEA: membrane-electrode assembly.

Supplementary Table 2 Co contents in GF-based electrodes as determined by ICP-OES.

Samples	Co contents
CoPc/GF	0.0033%
PyCoPc/GF	0.0046%

Supplementary Table 3 Crystal data and structure refinement for **A5** ($C_{13}H_{15}NO_4$).

Empirical formula	$C_{13}H_{15}NO_4$
Formula weight	249.26
Crystal system	orthorhombic
Space group	*Pbca*
Temperature(K)	278.7(10)
a/Å	13.8375(4)
b/Å	7.9610(2)
c/Å	21.9580(8)
α(deg)	90
β(deg)	90
γ(deg)	90
V/Å^3	2418.90(13)
Z	8
Dc(g.cm^{-3})	1.369
μ/mm^{-1}	0.102
Radiation	Mo $K\alpha$ (λ = 0.71073)
F(000)	1056.0
GOF on F^2	1.055
R1, wR2[$I > 2\sigma(I)$]α	0.0426, 0.1130
R1, wR2 (all data)α	0.0658, 0.1244

α: $R_1 = \sum||F_o| - |F_c||/\sum|F_o|$, $wR_2 = \{\sum w[(F_o)^2 - (F_c)^2]^2/\sum w[(F_o)^2]^2\}^{1/2}$

Supplementary Table 4 Fractional atomic coordinates (×10^4) and equivalent isotropic displacement parameters (Å^2×10^3) for A5 ($C_{13}H_{15}NO_4$).

Atom	*x*	*y*	*z*	U_{eq}(Å)
O1	2782.4(6)	6780.2(13)	4721.8(4)	72.2(3)
O2	1169.0(5)	5475.3(11)	5371.5(4)	61.6(2)
O3	5225.1(6)	7206.9(10)	6511.3(5)	71.0(3)
O4A	6398(4)	1555(6)	6972(2)	83.0(12)
O4B	6518(10)	1759(17)	6803(7)	78(3)
C1A	1185(2)	5697(4)	4700.4(13)	56.4(6)
C1B	1079(2)	6507(5)	4861.8(16)	64.9(8)
C2A	1893(2)	6217(5)	4436.2(14)	67.4(7)
C2B	1784(2)	7193(4)	4542.7(15)	66.8(7)
C3	2863.6(7)	6176.8(14)	5304.7(5)	48.8(2)
C4	2077.7(7)	5541.9(13)	5622.1(5)	48.6(3)
C5	2205.6(8)	4959.9(16)	6207.4(6)	60.0(3)
C6	3102.9(8)	4998.6(16)	6476.6(6)	59.6(3)
C7	3893.4(7)	5629.0(13)	6161.1(5)	48.5(3)
C8	3762.8(8)	6219.5(14)	5576.5(5)	51.6(3)
C9	4873.0(8)	5816.1(13)	6445.7(5)	51.3(3)
C10A	6359.3(13)	4582(2)	6807.5(11)	62.8(4)
C10B	6117(4)	4536(9)	7148(3)	68.3(15)
C11A	6581.7(19)	3146(3)	7228.2(12)	81.7(6)
C11B	6827(5)	3405(10)	6843(4)	84.3(19)
C12A	5429(2)	1440(3)	6788.2(11)	67.1(6)
C12B	5604(6)	1640(11)	6492(4)	82(2)
C13A	5159.7(12)	2758.2(19)	6330.1(8)	55.6(4)
C13B	4839(4)	2702(6)	6782(3)	60.9(11)
N1A	5382.9(15)	4422(3)	6569.7(9)	52.9(5)
N1B	5183(5)	4465(8)	6808(2)	53.1(14)

U_{eq} is defined as 1/3 of the trace of the orthogonalised U_{ij} tensor.

Supplementary Note 1: Technoeconomic analysis

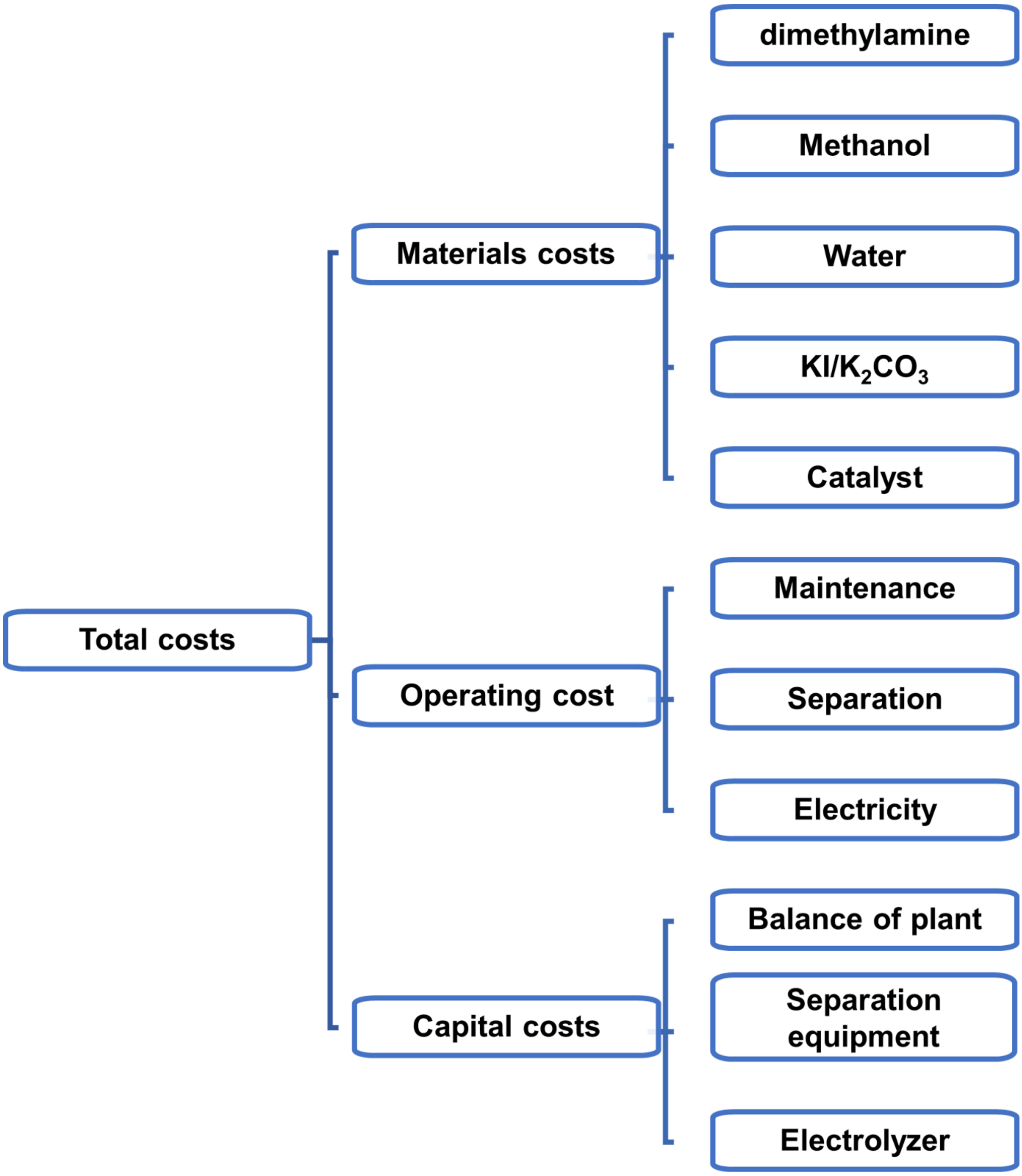

Supplementary Fig. 67 TEA model used for the production of DMF.

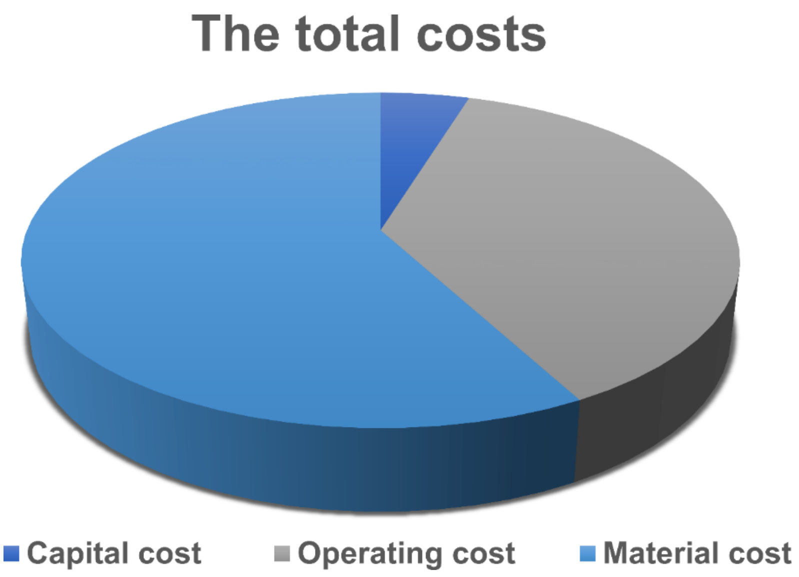

Supplementary Fig. 68 Total costs assessment in the TEA of DMF production.

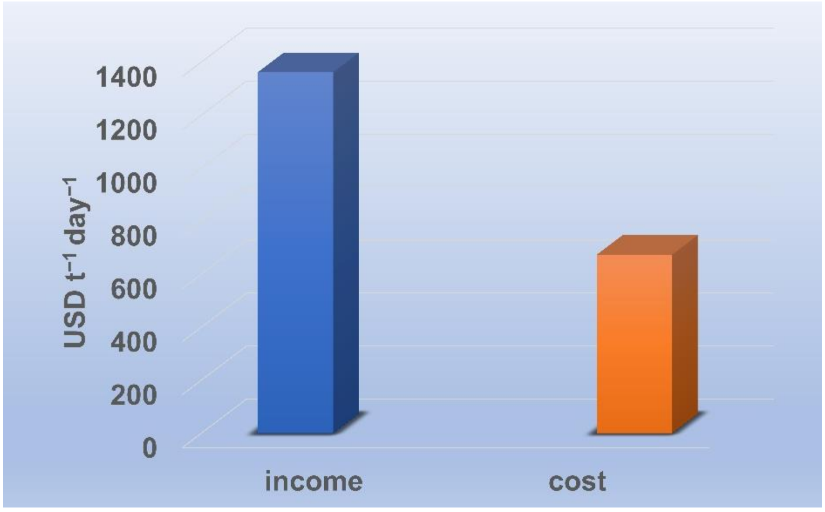

Supplementary Fig. 69 Income and cost assessment in the TEA of DMF production.

Techno-economic analysis (TEA) of the DMF electrosynthesis

A preliminary techno-economic analysis (TEA) of the DMF production was conducted based on a previously reported model with a modification.[13, 14] Some reasonable assumptions are made to produce 1 ton (t) of DMF per day.

Assumptions:

(1) In this work, the FE of DMF is approximately 73%; the cell voltage is approximately 1.0 V, and the operating current density is −200 mA cm^{-2} (Fig. 7).

(2) The lifetime of the plant is assumed to be 10 years, and the working time is set as 350 days per year.

(3) The cost of Balance of Plant is assumed to be 50% of the electrolyzer cost.

(4) The separation equipment cost is set as 150% of the electrolyzer cost.

(5) The electricity price is assumed to be 0.05 USD kWh^{-1}.

(6) The maintenance cost is set as 2.5% of the capital cost.

(7) The separation cost is set as 150% of the electricity cost.

(8) The market price of MeOH (99.9%, Shandong Alliance Chemical Group Co., Ltd. China) is approximately 264 USD t^{-1}, the market price of dimethylamine (40 wt%, Huainan Kedi Chemical Technology Co., Ltd. China) is approximately 215 USD t^{-1}, and the market price of DMF (99.9%, Shandong Jinyueyuan New Materials Co., Ltd. China) is approximately 1362 USD t^{-1}.

To produce 1 t of DMF, some basic parameters can be calculated:

(1) The required total charge was calculated as follows. The FE of DMF is 73% (1.0 V, 200 mA cm^{-2}) obtained from the electrolyzer.

Q = (Mass of DMF × F × n) / (Molar mass of DMF × FE of DMF) (1)

= 7.23×10^9 C

(2) The total current required was calculated as follows.

$I = Q/t$ (2)

= 83681 A

(3) The required area of the electrolyzer was calculated as follows.

Electrolyzer area = I / current density (3)

= 83681 A / 0.2 A cm^{-2}

= 41.84 m^2

(4) The power required was calculated as follows.

Power = 1.0 V × 83681 A = 83.68 kW

(5) The electrolyzer cost per area was calculated as follows.

Electrolyzer cost per area = 450 USD kW^{-1} × 1.0 V × 0.2 A cm^{-2}

= 900 USD m^{-2}

Capital cost:

(1) Electrolyzer

Electrolyzer cost = 41.84 m^2 × 900 USD m^{-2} = 37656 USD

(2) Separation Equipment

Separation equipment cost = 37656 USD × 150% = 56484 USD

(3) Balance of plant

Cost of balance of plant = 37656 USD × 50% = 18828 USD

Capital cost = (cost of electrolyzer + cost of balance of plant + cost of separation equipment) / (Lifetime of plant × m_{DMF} per day) (4)

= (37656 USD + 56484 USD +18828 USD) / (10 years × 350 days × 1 t)

= 32 USD t^{-1} day^{-1}

Operating cost:

(1) Electricity

Electricity cost = 83.68 kW × 24 h × 0.05 USD kWh^{-1}

= 100.4 USD t^{-1} day^{-1}

(2) Maintenance

Maintenance cost = 32 USD t^{-1} day^{-1} × 2.5% = 0.8 USD t^{-1} day^{-1}

(3) Separation

Separation cost = 100.4 USD t^{-1} day^{-1} × 150% = 150.6 USD t^{-1} day^{-1}

Operating cost = Electricity cost + Maintenance cost + Separation cost (5)

= 100.4 USD t^{-1} day^{-1} + 0.8 USD t^{-1} day^{-1} + 150.6 USD t^{-1} day^{-1}

= 251.8 USD t^{-1} day^{-1}

Material cost:

(1) MeOH

To produce 1 t of DMF per day, 0.44 t of MeOH is consumed.

MeOH cost = 0.44 t MeOH × 264 USD t^{-1} = 116.2 USD t^{-1} day^{-1}

(2) Dimethylamine

To produce 1 t of DMF per day, 1 t of dimethylamine is consumed.

Dimethylamine cost = 1 t of dimethylamine × 215 USD t^{-1} = 215 USD t^{-1} day^{-1}

(3) Catalyst

The catalyst PyCoPc loading is 3.83×10^{-2} mg cm^{-2} based on the ICP result of Co. The total PyCoPc required is calculated as follows:

Mass of PyCoPc = 3.83×10^{-2} mg cm^{-2} × 41.84 m^2 = 0.016 kg_{PyCoPc}

In our case, assuming the PyCoPc lifespan is 24 h, the number of catalyst changes is 1.

The catalyst cost required per day for 1 t of DMF production can be calculated below.

Catalyst cost = 3571 USD kg_{PyCoPc}^{-1} × 0.016 kg_{PyCoPc} × 1 t^{-1} day^{-1}

= 57 USD t^{-1} day^{-1}

(4) Water, K_2CO_3 and KI

Based on the local water price, the water cost is approximately 0.36 USD t^{-1} day^{-1}. The K_2CO_3 cost is set as 200% of the water cost, which is approximately 0.72 USD t^{-1} day^{-1}. The KI cost is set as 100% of the water cost, which is approximately 0.36 USD t^{-1} day^{-1}.

Material cost = MeOH cost + Dimethylamine cost + Catalyst cost + Water cost + K_2CO_3 cost + KI cost (6)

= 116.2 USD t^{-1} day^{-1} + 215 USD t^{-1} day^{-1} + 57 USD t^{-1} day^{-1} + 0.36 USD t^{-1} day^{-1} + 0.72 USD t^{-1} day^{-1} + 0.36 USD t^{-1} day^{-1}

= 389.6 USD t^{-1} day^{-1}

The total cost:

The total cost = Capital cost + Operating cost + Material cost (7)

= 32 USD t^{-1} day^{-1} + 251.8 USD t^{-1} day^{-1} + 389.6 USD t^{-1} day^{-1}

= 673.4 USD t^{-1} day^{-1}

The daily profit:

Daily profit = income – the total cost (8)

= 1362 USD t^{-1} day^{-1} − 673.4 USD t^{-1} day^{-1}

= 688.6 USD t^{-1} day^{-1}

REFERENCE

[1] G. P. Mrug, S. P. Bondarenko, V. P. Khilya, M. S. Frasinyuk, Synthesis and aminomethylation of 7-hydroxy-5-methoxyisoflavones. *Chem. Nat. Compd.* **2013**, *49*, 235-241.

[2] H. Heaney, G. Papageorgiou, R. F. Wilkins, The generation of iminium ions using chlorosilanes and their reactions with electron rich aromatic heterocycles. *Tetrahedron* **1997**, *53*, 2941-2958.

[3] J. Kan, Y. Chen, J. Gao, L. Wan, T. Lei, P. Ma, J. Jiang, Synthesis, self-assembly, and semiconducting properties of phenanthroline-fused phthalocyanine derivatives. *J. Mater. Chem.* **2012**, *22*, 15695.

[4] P. Ma, J. Kan, Y. Zhang, C. Hang, Y. Bian, Y. Chen, N. Kobayshi, J. Jiang, The first solution-processable n-type phthalocyaninato copper semiconductor: tuning the semiconducting nature via peripheral electron-withdrawing octyloxycarbonyl substituents. *J. Mater. Chem.* **2011**, *21*, 18552.

[5] J. Su, J.-J. Zhang, J. Chen, Y. Song, L. Huang, M. Zhu, B. I. Yakobson, B. Z. Tang, R. Ye, Building a stable cationic molecule/electrode interface for highly efficient and durable CO_2 reduction at an industrially relevant current. *Energy Environ. Sci.* **2021**, *14*, 483-492.

[6] C. Zhao, X. Dai, T. Yao, W. Chen, X. Wang, J. Wang, J. Yang, S. Wei, Y. Wu, Y. Li, Ionic exchange of metal-organic frameworks to access single nickel sites for efficient electroreduction of CO_2. *J. Am. Chem. Soc.* **2017**, *139*, 8078-8081.

[7] Y. He, X. Xiao, X. Li, D.-D. Ma, Q.-L. Zhu, Q. X., Unlocking high-efficiency electrosynthesis of *N,N*-dimethylformamide through the synergy of three-dimensional ordered macroporous superstructure and implanted nano-tentacles. *Appl. Catal. B-Environ.* **2026**, *382*, 125989.

[8] M. Jin, A.-Z. Li, Y. Wang, J. Li, H. Zhou, B.-J. Li, H. Duan, Electrosynthesis of *N,N*-dimethylformamide from market-surplus trimethylamine coupled with hydrogen production. *Green Chem.* **2023**, *25*, 5936-5944.

[9] W. Li, H. Jiang, X. Zhang, B. Lei, L. Li, H. Zhou, M. Zhong, Sustainable electrosynthesis of *N,N*-dimethylformamide via relay catalysis on synergistic active

sites. *J. Am. Chem. Soc.* **2024**, *146*, 21968-21976.

[10] Y. Fan, T. Liu, Y. Yan, Z. Xia, Y. Lu, Y. Pan, R. Wang, D. Xie, Z. Zhu, T. T. T. Nga, C.-L. Dong, Y. Jing, Y. Li, S. Wang, Y. Zou, Electrochemical synthesis of formamide by C–N coupling with amine and CO_2 with a high faradaic efficiency of 37.5%. *Chem* **2024**, *10*, 2437-2449.

[11] S. Yan, S. Chen, M. McKee, A. Terry, R. Weisbarth, N. Kornienko, Total electrosynthesis of *N,N*-dimethylformamide from CO_2 and NO_3^-. *Adv. Sci.* **2024**, *12*, 2414431.

[12] J. Zheng, S. Xu, L. Sun, X. Pan, Q. Xie, G. Zhao, Boosting the reduction of CO_2 and dimethylamine for C–N bonding to synthesize DMF via modulating the electronic structures of indium single atoms. *Energy Environ. Sci.* **2025**, *18*, 3614-3622.

[13] J. Li, X. Liu, S.-M. Xu, M. Xu, Y. Wang, Y. Lyu, A.-Z. Li, Y. Wang, X. Wang, T. Zhou, H. Zhou, Y. Peng, X. Li, L. Zheng, H. Duan, Sustainable oxime production via the electrosynthesis of hydroxylamine in a free state. *Nat. Synth.* **2025**, *4*, 1598–1609.

[14] N. Meng, J. Shao, H. Li, Y. Wang, X. Fu, C. Liu, Y. Yu, B. Zhang, Electrosynthesis of formamide from methanol and ammonia under ambient conditions. *Nat. Commun.* **2022**, *13*, 5452.
