# Supplementary material for: Redox-mediated domino electrosynthesis of N,N-dimethylformamide with industrial-relevant productivity and modularized cathodic integration
Source: Nat Commun. 2026 Apr 17;17:5349. doi: 10.1038/s41467-026-71637-z (PMC13272958; doi:10.1038/s41467-026-71637-z)
Supplement: Supplementary file 2 — Description of Additional Supplementary Files [file 41467_2026_71637_MOESM2_ESM.pdf]

Description of Additional Supplementary Files

File Name: Supplementary Data 1

Description: DFT calculation data for Figure 5b.
