# Supplementary material for: Redox-mediated domino electrosynthesis of N,N-dimethylformamide with industrial-relevant productivity and modularized cathodic integration
Source: Nat Commun. 2026 Apr 17;17:5349. doi: 10.1038/s41467-026-71637-z (PMC13272958; doi:10.1038/s41467-026-71637-z)
Supplement: Supplementary file 4 — Transparent Peer Review File [file 41467_2026_71637_MOESM4_ESM.pdf]

nature portfolio

Peer Review File

Redox-Mediated Domino Electrosynthesis of N,N-Dimethylformamide with Industrial-Relevant Productivity and Modularized Cathodic Integration

Corresponding Author: Professor Qi-Long Zhu

This file contains all reviewer reports in order by version, followed by all author rebuttals in order by version.

Attachments originally included by the reviewers as part of their assessment can be found at the end of this file.

Version 0:

Reviewer comments:

Reviewer #1

(Remarks to the Author)
See attached file

Reviewer #2

(Remarks to the Author)
In this work, the authors report a I2-mediated DMF electrolysis and achieve a great electrochemical performance. However, the reviewer considers a major revision is necessary for publishing in Nature Communications.
1. The catalyst characterization should be supplemented. The XRD patters only shows a characteristic peak of GC, which may be attributed the low loading of PyCoPc. ICP-OES or ICP-MS of PyCoPc/GC and XRD of pure PyCoPc are necessary. Besides, the SEM images with higher magnification and high-resolution TEM should be added to reveal the surface morphology and structure. Furthermore, the AC-STEM is also suggested to conduct.
2. For the electrochemical study, the detailed LSV curves under different conditions should be added (various electrodes, and KI concentrations). Besides, is O2 or I2 generated during the reaction? Furthermore, the usage of BPM shows a slight increase of FEDMF while it may also raise the cell voltage. The authors consider that the enhancement originates from cross-over of anodic reactants, while the phenomenon of cross-over is not noticeable in H cell. So, this opinion is unconvincing and the authors should consider carefully the enhancement after using BPM. Furthermore, there is no obvious decay after 150 hours Why not continue testing? Does the amount of KI change after the reaction? The concentration of KI after the reaction should also be quantified to evaluate the potential degradation. The post-reaction characterization of the electrode should be analyzed deeply.
3. The assignment of the peaks near 1650 cm-1 may be attributed to interfacial water and the authors should analyze the results of in situ ATR-IR carefully. In addition, the poor yield of DMF from electrooxidation of TMDM is abnormal and should be carefully examined. What are the other products in this reaction. In the Figure 4e, the I2 and *CH2O species may generate simultaneously and the authors should consider carefully. It is recommended that authors carefully describe the reaction mechanism in the Introduction section to help readers understand.
4. The authors should consider whether a carbonate-mediated pathway could be involved in the reaction? The isotopic labeling experiment may be beneficial for mechanism studies. Similarly, is this I2-mediated strategy universally applicable? For instance, could this strategy be extended to the electrooxidation of methanol (MeOH) and other nitrogenous species? Furthermore, the technoeconomic assessment is useful to evaluate economic viability.
5. The 2.4 section has little relevance to the central theme of this article. The authors should discuss more clearly how this section supports the key I2-mediated strategy. The -200 mA in Figure 6c should be changed into -200 mA cm-2.

Reviewer #3

(Remarks to the Author)
My suggestions are as follows:

1. There is a mismatch between the title and the manuscript content: certain key aspects (e.g., the cathodic reaction) are not adequately reflected in the title. Additionally, the description of Ampakine synthesis in the abstract refers to a non-electrochemical process, which is inconsistent with the title’s focus on electrosynthesis.
2. The authors should elaborate on the significance of Ampakine (e.g., its pharmacological value or industrial relevance) in the Introduction section.
3. X-ray absorption spectroscopy (XAS) data of commercial CoPc should be included for comparison, to clarify the influence of peripheral pyrrolidone substituents on the intrinsic Co-N4 sites.
4. The current characterization techniques employed (e.g., XRD, XAS, XPS) are insufficient to confirm the single-molecular integrated structure of PyCoPc. The authors are required to employ additional techniques such as HRTEM to verify this—given that CoPc molecules may form a multilayer structure via interfacial π-π stacking interactions. Simultaneously, the uniformity of PyCoPc loading on the GF support should be validated.
5. A detailed process for the stability test conducted in the H-type cell should be provided. The conversion rate of dimethylamine (DMA) must be calculated, and LSV curves for DMF electrosynthesis in the presence and absence of KI should be supplied to verify the mediating role of I-.
6. The cobalt content in the PyCoPc/GF catalyst should be quantified.
7. The effect of I- on the formation of the *CH2O intermediate should be clarified through in situ ATR-IR characterization, including control experiments without KI over PyCoPc/GF.
8. A detailed explanation for the peak shift observed in the quasi-in situ NMR spectrum (Figure 4f) is required.
9. The influence of the reaction solution composition (line 346) on the reaction mechanism should be elucidated. The bipolar membrane employed in the flow system may enhance catalytic activity by facilitating OH- transport; thus, additional studies to investigate the effect of pH on DMF electrosynthesis activity are recommended. Furthermore, in contrast to CO2-saturated catholyte, the introduction of CO2 into an alkaline solution may fail to maintain the stability of the cathodic environment—this phenomenon’s implications on overall reaction performance should be assessed.

Version 1:

Reviewer comments:

Reviewer #1

(Remarks to the Author)
All questions made to authors were clarified and new data analysis were incorporated in the manuscript, therefore I recommend the manuscript for publication.

Reviewer #2

(Remarks to the Author)
The authors have addressed my comments and the current manuscript could be accepted in Nature Communications.

Reviewer #3

(Remarks to the Author)
The revised manuscript is suitable for publication.

Open Access This Peer Review File is licensed under a Creative Commons Attribution 4.0 International License, which permits use, sharing, adaptation, distribution and reproduction in any medium or format, as long as you give appropriate credit to the original author(s) and the source, provide a link to the Creative Commons license, and indicate if changes were made.
In cases where reviewers are anonymous, credit should be given to 'Anonymous Referee' and the source.
The images or other third party material in this Peer Review File are included in the article's Creative Commons license, unless indicated otherwise in a credit line to the material. If material is not included in the article's Creative Commons license and your intended use is not permitted by statutory regulation or exceeds the permitted use, you will need to obtain permission directly from the copyright holder.
To view a copy of this license, visit https://creativecommons.org/licenses/by/4.0/

Response letter

We would like to express our sincere thanks to the reviewers for their valuable comments and constructive suggestions. We have tried our best to revise our manuscript according to the suggestions by the reviewers. The changes made based on review comments in the revised manuscript have been highlighted in Yellow. In addition, we made the required changes to the article's formatting. The format-related changes are highlighted in Blue. The following is the point-by-point response to the comments made by the reviewers:

Response to comments from the reviewer # 1:

General Comments. In the related manuscript, the authors propose a molecularly modified electrode (PyCoPc/GF) for selective methanol oxidation, coupled with a CO_2 reduction process in a bipolar membrane flow cell. The idea of using molecular Co phthalocyanine sites for oxidation–reduction paired electrosynthesis is **scientifically appealing**. However, several key aspects of the electrochemical characterization, mechanistic elucidation, and catalyst stability are insufficiently supported by data. Therefore, I do not recommend acceptance in its current form, but the work could be reconsidered after major revision and substantial experimental clarification. Below are specific, constructive points to improve the manuscript, listed point-by point.

Response to Reviewer 1:

We would like to express our sincere thanks to this reviewer for her/his valuable comments and constructive suggestions, which are helpful to significantly improve the manuscript. We have carefully revised the manuscript according to your comments. The following is the response to each comment.

Comment 1.

No cyclic voltammograms (CVs) or linear sweep voltammograms (LSVs) are shown for the anode in methanol oxidation conditions. The SI mentions such measurements, but the only voltammograms displayed correspond to CO_2 reduction. Please include LSV/CV data for PyCoPc/GF, CoPc/GF, and GF electrodes in the actual anolyte (MeOH:DMA/K_2CO_3 ± KI). These are crucial to demonstrate intrinsic activity, onset

potentials, and kinetic differences.

Response: Thanks for your comment. We have supplemented the manuscript with LSV curves obtained under varying KI concentrations (Supplementary Fig. 19) and for different catalysts (Supplementary Fig. 25). As shown in Supplementary Fig. 19, the addition of KI significantly increased the current and induced a negative shift in the onset potential, from approximately 0.42 V without KI to about 0.30 V with 100 mM KI. Furthermore, Supplementary Fig. 25 reveals that PyCoPc/GF exhibits a significantly higher current density than both CoPc/GF and GF, and a much lower onset potential (0.30 V) compared to 0.44 V for CoPc/GF and 0.53 V for GF, demonstrating its superior catalytic activity.

Changes made in Revised Manuscript:

Page 9, lines 24-27.

Linear sweep voltammetry (LSV) was initially employed to evaluate the effect of KI on the catalytic reaction. As shown in Supplementary Fig. 19, the addition of KI significantly increased the current and induced a negative shift in the onset potential, from approximately 0.42 V without KI to about 0.30 V with 100 mM KI.

Page 11, lines 14-16.

PyCoPc/GF exhibits a significantly higher current density than both CoPc/GF and GF, and a much lower onset potential of 0.30 V compared to CoPc/GF (0.44 V) and GF (0.53 V), demonstrating its superior catalytic activity (Supplementary Fig. 25).

Changes made in Revised Supporting Information:

Page 27

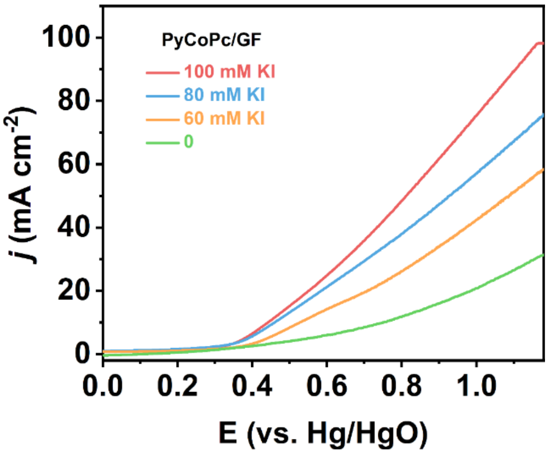

Supplementary Fig. 19 LSV curves of PyCoPc/GF with different concentrations of KI.

Page 33

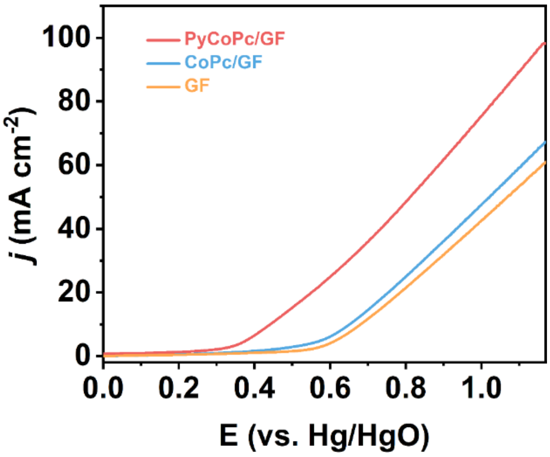

Supplementary Fig. 25 LSV curves of PyCoPc/GF, CoPc/GF and GF in 0.7 M K_2CO_3 with 100 mM KI.

Comment 2.

In the SI, it is mentioned that Pt counter electrode was used in a three-electrode compartment cell. even in iodide-containing media and under strongly cathodic potentials. Platinum can undergo cathodic corrosion, forming soluble species that may redeposit on the anode, compromising the catalytic interpretation. Could authors, please comment on that?

Response: Thanks for the comment. All electrochemical tests employed a membrane to separate the cathode and anode, thereby preventing corrosion-induced redeposition of the cathode catalyst onto the anode and avoiding adverse effects on catalytic performance. Except for the CO_2RR reaction at the cathode where a cation-exchange membrane was used, a bipolar membrane was employed for all other tests. We have clarified this point in the Supporting Information.

Changes made in Revised Supporting Information:

Page 4, lines 1-3.

For general reaction conditions, a bipolar membrane was employed to separate the compartments of the H-type electrolytic cell…

Page 5, lines 15-16.

Electrolysis-paired tandem synthesis was performed in a bipolar membrane separated H-

type electrolytic cell.

Page 6, line 28.

Flow cell test was performed in a bipolar membrane separated flow cell.

Comment 3.

The claim that "the architecture maintains structural integrity" is not supported beyond post-run SEM/EDX and XRD. As this is an important discussion/interpretation of the manuscript, more details post electrolysis should be provided.

Response: Thanks for your comment. We further characterized the post-reaction electrode using UV-Vis spectroscopy, XPS and Raman spectroscopy, which further confirmed the preservation of the catalyst structure. Prior to characterization, the post-reaction samples were sequentially rinsed with deionized water and methanol, followed by vacuum drying to remove surface contaminants. The UV-Vis spectrum of the post-reaction PyCoPc/GF retained the characteristic phthalocyanine absorption peak at 710 nm (Supplementary Fig. 29). Similarly, the Raman spectrum preserved the characteristic phthalocyanine peaks (Supplementary Fig. 30). Furthermore, the XPS spectrum confirmed the Co^{2+} peak position in the Co $2p_{3/2}$ region remained at approximately 780.4 eV, exhibiting no significant shift (Supplementary Fig. 31).

Changes made in Revised Supporting Information:

Page 4, lines 15-19

Upon completion of the test, to eliminate residual electrolytes and reaction products that could interfere with post-test electrode characterization, the electrodes were sequentially subjected to copious rinsing with deionized water, followed by ultrasonication in a beaker for 10 minutes, and then rinsed thoroughly with methanol three times. Finally, the electrodes were dried overnight in a vacuum oven at 60 °C under continuous vacuum.

Page 37.

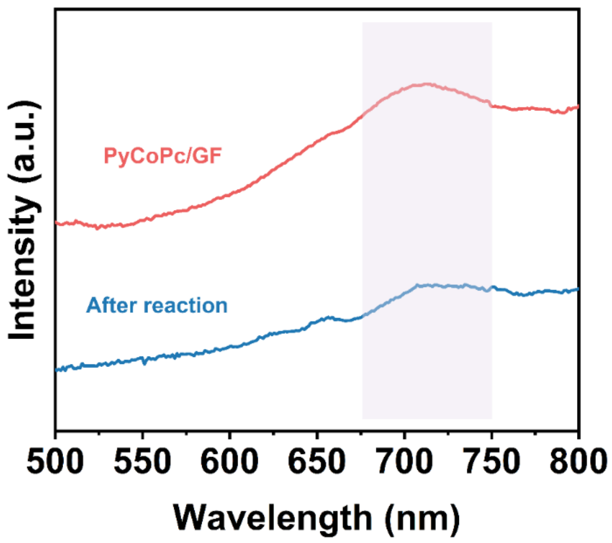

Supplementary Fig. 29 UV-Vis spectra of PyCoPc/GF before and after reaction.

Prior to characterization of the post-reaction catalyst, samples were sequentially rinsed with deionized water and methanol, followed by vacuum drying to remove surface contaminants. Detailed procedures are documented in the Experimental Section.

The Py-CoPc/GF electrode was subjected to pulverization and dispersion in methanol solution for characterization. After reaction, the characteristic phthalocyanine peak remained detectable at approximately 710 nm.

Page 38.

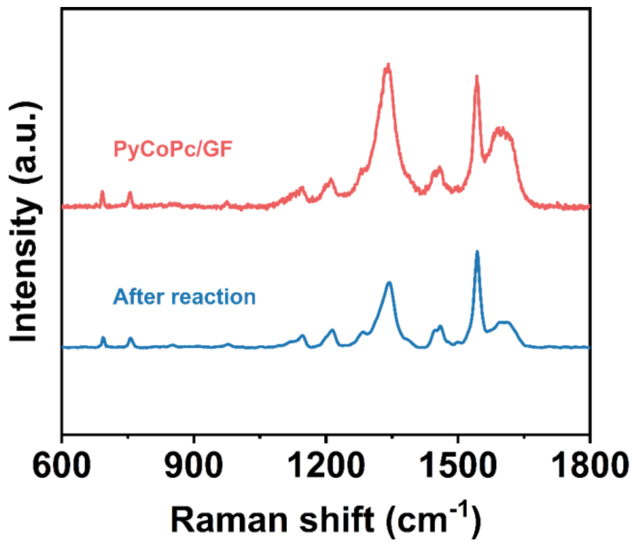

Supplementary Fig. 30 Raman spectra of PyCoPc/GF before and after reaction.

Page 39.

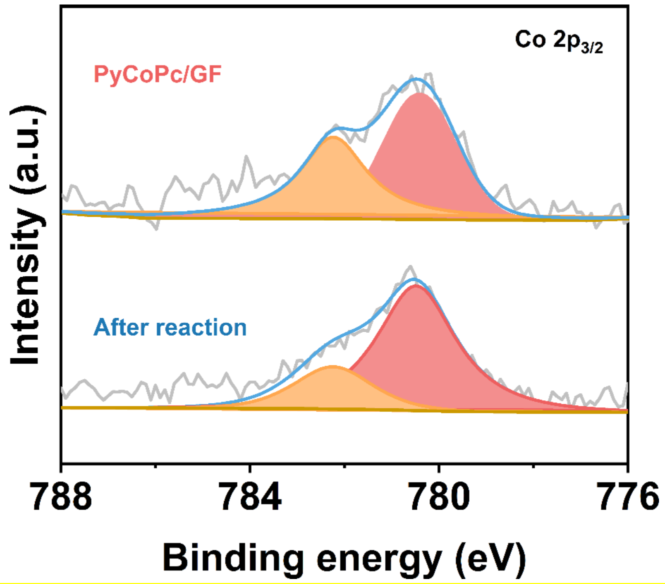

Supplementary Fig. 31 Co 2p XPS spectra of PyCoPc/GF before and after reaction.

After the reaction, the Co $2p_{3/2}$ XPS spectrum of PyCoPc/GF showed that the Co^{2+} peak was observed at 780.4 eV with no significant shift.

Changes made in Revised Manuscript:

Page 11, line 30.

Furthermore, the long-term stability test for 150 h demonstrates that PyCoPc/GF exhibits a consistent catalytic activity, capable of well maintaining the high FE_{DMF} (~90%), productivity (~839 μmol h^{-1} cm^{-2}) for DMF production (Fig. 3e and Supplementary Figs. 28-33).

Comment 4.

The proposed mechanism involves methanol oxidation to formaldehyde, followed by iodide-mediated transformation of TMDM to iminium and finally to DMF. However, the initial methanol oxidation step on PyCoPc/GF is not directly monitored. Please, provide formaldehyde quantification (e.g., by Nash test or GC/HPLC) during electrolysis to verify this step, and include control experiments without iodide to distinguish the intrinsic activity of PyCoPc from redox mediation by I_2/I^-.

Response: We thank the reviewers for their constructive comments. As the reviewers noted, detecting formaldehyde is crucial for understanding the reaction mechanism. However, DMA and formaldehyde can react rapidly under mild conditions to form TMDM (*Chem. Nat. Compd.* 2013, 49, 235-241; *Tetrahedron* 1997, 53, 2941-2958). In

our reaction system (15 mL of 0.7 M K_2CO_3, 12 mL of CH_3OH, 4 mL of DMA, 100 mM KI), the concentration of DMA reaches 1.05 M, enabling the electrochemically generated $^*CH_2O$ intermediate to react quickly and form TMDM. To further verify this reaction, we monitored it using NMR. Specifically, we took 500 µL of electrolyte (containing 525 µmol of DMA), added 100 µL of D_2O, and then added 4 µL of formaldehyde aqueous solution (53 µmol of HCHO). The mixture was rapidly shaken, and NMR detection was performed within less than 2 min after formaldehyde addition. The NMR results confirmed that formaldehyde and DMA rapidly generate TMDM.

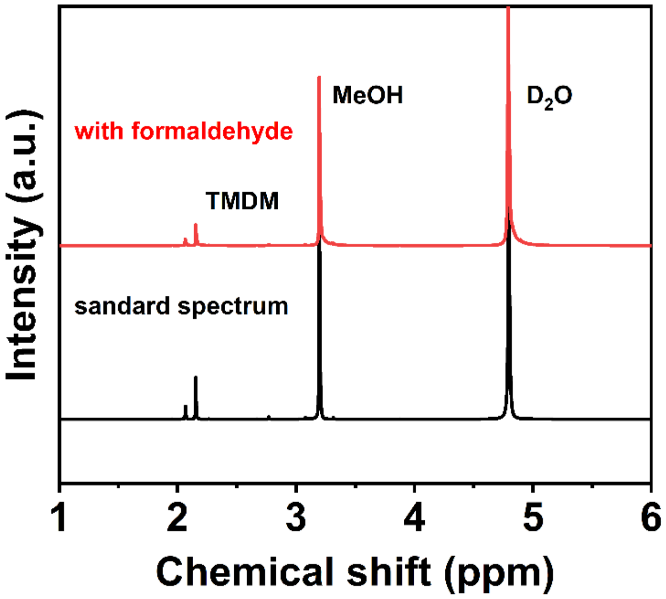

Changes made in Revised Supporting Information:

Page 10.

It should be noted that formaldehyde and DMA can rapidly react to form TMDM,[12, 13] making direct quantification of formaldehyde difficult, especially in the presence of high DMA concentrations.

Page 85, line 29 to Page 86 line 4.

[12] G. P. Mrug, S. P. Bondarenko, V. P. Khilya, M. S. Frasinyuk, Synthesis and aminomethylation of 7-hydroxy-5-methoxyisoflavones. *Chem. Nat. Compd.* **2013**, *49*, 235-241.

[13] H. Heaney, G. Papageorgiou, R. F. Wilkins, The generation of iminium ions using chlorosilanes and their reactions with electron rich aromatic heterocycles. *Tetrahedron* **1997**, *53*, 2941-2958.

Comment 5.

The manuscript attributes high performance to "increased exposure of active sites," but no electrochemically active surface area (ECSA) or Co site loading is reported. Please, provide double-layer capacitance (C_{dl}) or other ECSA measurements, Co loading by ICP, and ideally a turnover frequency (TOF) normalized to active Co sites.

Response: Thanks for the suggestions. As shown in Supplementary Fig. 26, PyCoPc/GF exhibits a C_{dl} value of 0.06 mF cm^{-2}, significantly higher than those of CoPc/GF (0.0076 mF cm^{-2}) and GF (0.0065 mF cm^{-2}). This indicates substantially more accessible active sites in PyCoPc/GF, which can be attributed to the pyrrolidone groups facilitating effective anchoring of PyCoPc molecules onto the GF substrate. These conclusions are consistent with ICP-OES results (Supplementary Table 2), showing cobalt loadings of 0.0046% for PyCoPc/GF versus 0.0033% for CoPc/GF. Furthermore, the enhanced intrinsic activity was corroborated by TOF measurements. It should be noted that while the C_{dl} was calculated, the exact number of catalytically active sites could not be directly determined. Therefore, the Co loading obtained from ICP analysis was utilized. Based on literature precedent (*Energy Environ. Sci.* 2021, 14, 483-492; *J. Am. Chem. Soc.* 2017, 139, 8078-8081), the ICP-measured Co content was approximated as the number of active sites. The measured TOF for PyCoPc/GF was 82.54 s^{-1}, higher than that for CoPc/GF (67.52 s^{-1}).

Changes made in Revised Manuscript:

Page 11, lines 21-22.

…as evidenced by electrochemical active surface area measurements (Supplementary Fig. 26).

Page 8, lines 17-19.

The Co content of PyCoPc/GF (0.0046%) is higher than that in CoPc/GF (0.0033%) (Supplementary Table 2).

Page 11, lines 24-27.

The turnover frequency (TOF) measurements were further corroborated the enhanced intrinsic activity. As shown in Supplementary Fig. 27, the measured TOF for PyCoPc/GF was 82.54 s^{-1}, higher than that for CoPc/GF (67.52 s^{-1}).

Changes made in Revised Supporting Information:

Page 34.

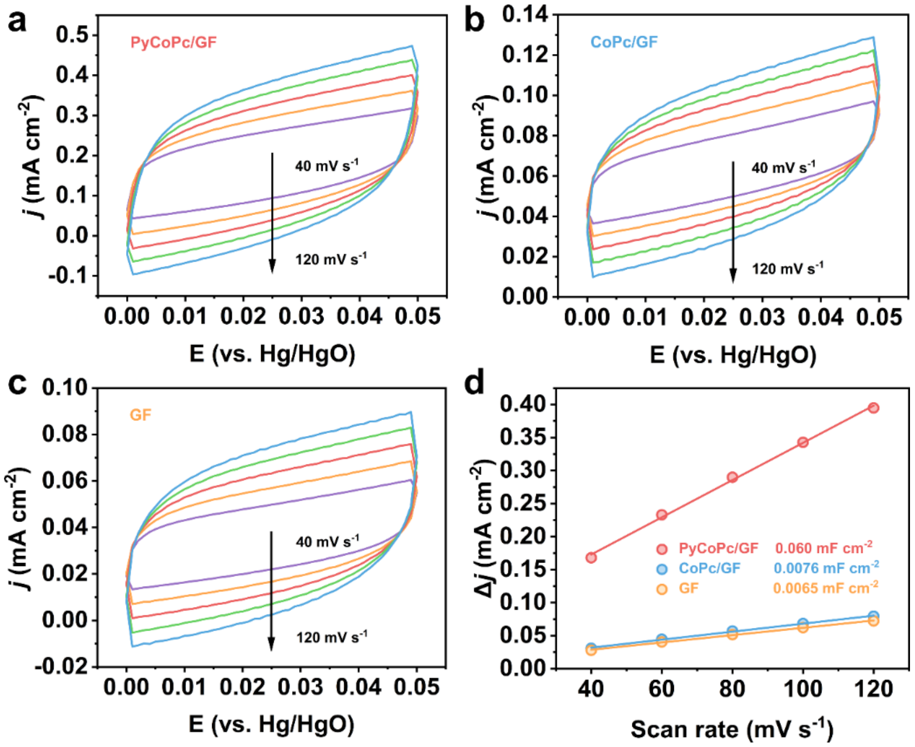

Supplementary Fig. 26 CV curves of (a) PyCoPc/GF, (b) CoPc/GF and (c) GF at different scan rates; (d) charge current density difference against scan rates.

The electrochemically active surface areas (ECSAs) can be used to evaluate the degree of exposure of active sites in catalysts and are typically proportional to the double-layer capacitance (C_{dl}). As shown in Supplementary Fig. 26d, PyCoPc/GF exhibits a significantly higher C_{dl} value than CoPc/GF and GF, indicating enhanced accessibility of active sites. This improvement is attributed to the pyrrolidone groups facilitating the anchoring of PyCoPc molecules onto the GF substrate, consistent with ICP-OES results (Supplementary Table 2).

Page 76.

Supplementary Table 2 Co contents in GF-based electrodes as determined by ICP-OES.

Samples	Co contents
CoPc/GF	0.0033%
PyCoPc/GF	0.0046%

Page 35.

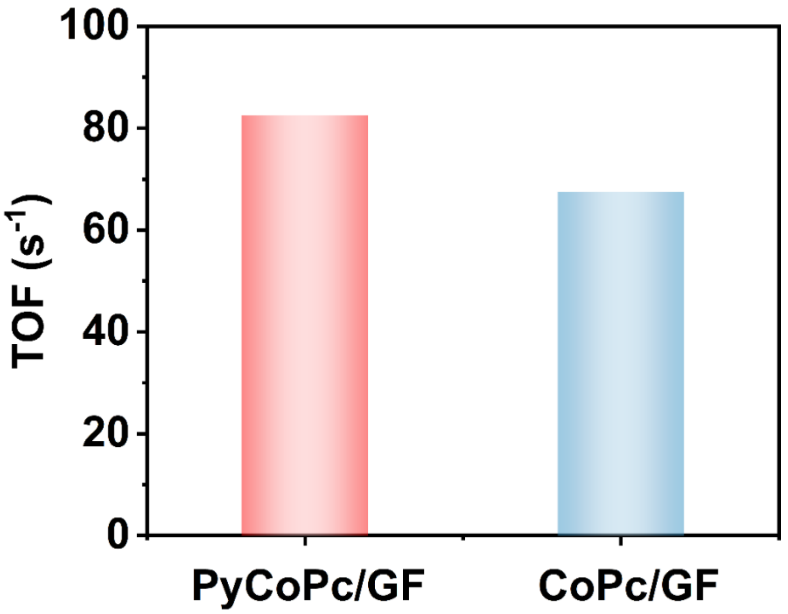

Supplementary Fig. 27 TOF values of PyCoPc/GF and CoPc/GF for DMF production.

It should be noted that the Co content determined by ICP can be approximated as the active site count in the TOF calculation.[16, 17]

Page 85, lines 12-18.

[16] J. Su, J.-J. Zhang, J. Chen, Y. Song, L. Huang, M. Zhu, B. I. Yakobson, B. Z. Tang, R. Ye, Building a stable cationic molecule/electrode interface for highly efficient and durable CO_2 reduction at an industrially relevant current. *Energy Environ. Sci.* **2021**, *14*, 483-492.

[17] C. Zhao, X. Dai, T. Yao, W. Chen, X. Wang, J. Wang, J. Yang, S. Wei, Y. Wu, Y. Li, Ionic exchange of metal-organic frameworks to access single nickel sites for efficient electroreduction of CO_2. *J. Am. Chem. Soc.* **2017**, *139*, 8078-8081.

Comment 6.

The mediator plays a central role, but its long-term stability is not examined. Please analyze iodide/iodine concentrations before and after extended electrolysis (e.g., by UV–vis or iodometric titration) to confirm that the mediator is not depleted or transformed irreversibly.

Response: Thanks for the comment. During extended stability testing, the electrolyte was replenished every 10 h (see Section *1.3.1, Electrochemical Measurements*), maintaining

the mediator concentration well above the reaction stoichiometry. Throughout the reaction, a portion of the generated I_2 participated in the reaction, while the excess I_2 exists in equilibrium with I^- in the electrolyte via the reaction $I_3^- \rightleftharpoons I_2 + I^-$. Both forms are considered part of the effective iodine content. Post-reaction, I_2 and I_3^- were quantitatively reduced to I^- using Na_2SO_3, and the I^- concentration was determined by UV-Vis spectrophotometry. Analysis revealed an active iodine content of 92 mM after reaction (Supplementary Fig. 34), compared to the initial KI concentration of 100 mM, demonstrating the reversibility and stability of the iodine-based mediator system under operational conditions.

Changes made in Revised Supporting Information:

Page 42.

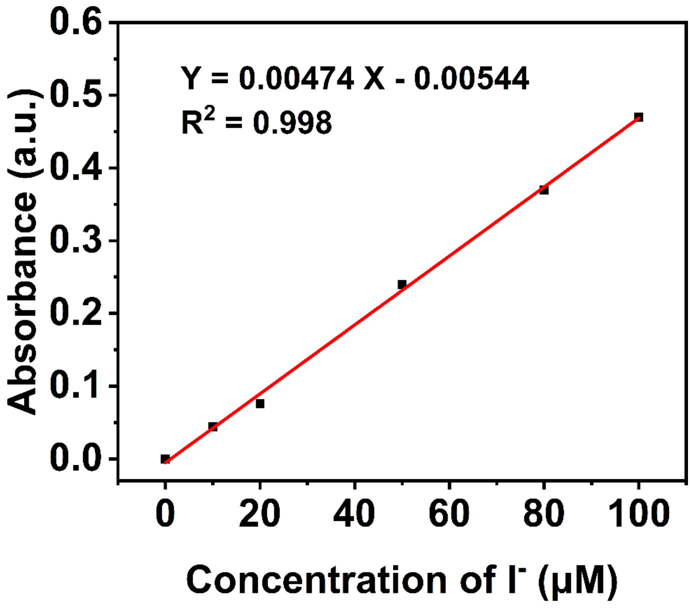

Supplementary Fig. 34 UV-Vis spectrophotometric calibration curve for I^- quantification.

During extended stability testing, the electrolyte was replenished every 10 h, maintaining the mediator concentration well above the reaction stoichiometry. Throughout the reaction, a portion of the generated I_2 participated in the reaction, while the excess I_2 exists in equilibrium with I^- in the electrolyte via the reaction $I_3^- \rightleftharpoons I_2 + I^-$. Both forms are considered part of the effective iodine content. Post-reaction, I_2 and I_3^- were quantitatively reduced to I^- using Na_2SO_3, and the I^- concentration was determined by UV-Vis spectrophotometry.

Changes made in Revised Manuscript:

Page 11 line 30 to Page 12 line 3.

Critically, quantification of active iodine species post-reaction revealed 92 mM residual concentration (Supplementary Fig. 34), representing 92% retention compared to the initial KI concentration (100 mM), confirming mediator reversibility and system stability.

Comment 7.

Recent literature (Broersen et al. *J. Am. Chem. Soc. 2025, 147, 12, 10298–10308*), showed the electrochemical *N*-formylation of amines via methanol oxidation occurs via iso(cyanides) intermediate. Please provide details about parallel reaction.

Response: Thanks for the comment. We have carefully reviewed the manuscript referenced by the reviewer, which reports the electrochemical synthesis of *N*-methylformamide from methanol and methylamine in $NaClO_4$, NaOH or H_2SO_4 electrolytes. *In situ* FTIR and control experiments reveal two distinct reaction pathways: direct oxidation of a hemiaminal intermediate and a novel route involving the formation of methylisocyanide, which subsequently undergoes hydration to yield *N*-methylformamide. Notably, in addition to the $NaClO_4$ system, characteristic peaks attributable to isocyanide intermediates were observed at 2145 cm^{-1} in the *in situ* FTIR spectra of both NaOH and H_2SO_4 electrolytes.

However, detailed analysis of our *in situ* ATR-IR spectra detected no significant signals between 2100 and 2200 cm^{-1}, suggesting the absence of cyanide or isocyanide intermediates in our system (Supplementary Fig. 45). Besides, formaldehyde react rapidly with dimethylamine form tetramethylmethanediamine (TMDM). Through controlled experiments and *quasi-in situ* NMR spectroscopy (Fig. 4a-d), we identified key intermediates: an iodinated species (1-iodo-*N,N,N',N'*-tetramethylmethanediamine) and an iminium cation ((dimethylaminomethylene)dimethylammonium cation). Notably, Zhang et al. (*Nat. Commun.* 2022, 13, 5452) reported that during electrochemical formamide synthesis from methanol and NH_3 in 0.5 M $NaHCO_3$ electrolyte, the α-PtO_2 catalyst exhibits dual reaction pathways involving both hemiaminal and nitrile intermediates, whereas β-NiOOH and α-FeOOH catalysts follow only the hemiacetal

pathway. These findings suggest that the reaction mechanism may be significantly influenced by factors such as electrolyte composition and catalyst.

Changes made in Revised Supporting Information:

Page 53

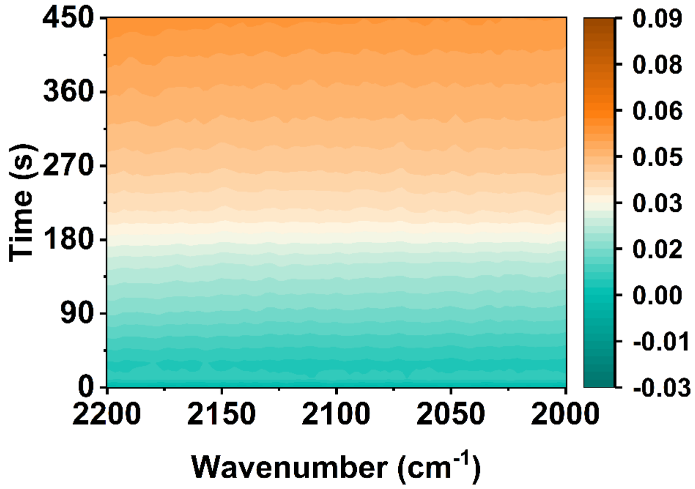

Supplementary Fig. 45 Magnified view of *in situ* ATR-IR spectrum for the DMF electrosynthesis over PyCoPc/GF in 0.7 M K_2CO_3 with 100 mM KI.

Changes made in Revised Manuscript:

Page 14, lines 10-14.

Recent literature suggests that amide formation may proceed via intermediates such as methylisocyanide or nitrile species.[34, 35] However, our in situ ATR-IR spectroscopy analysis detected no significant signals in the 2100–2200 cm^{-1} region (Supplementary Fig. 45). This absence may arise from the significant influence of factors such as electrolyte composition and catalyst on the reaction mechanism.

Page 24, lines 26-32.

[34] P. J. L. Broersen, L. Wielhouwer, G. Rothenberg, A. C. Garcia, Electrochemical N-Formylation of Amines: Mechanistic Insights and Sustainable Synthesis of Formamides via a Methylisocyanide Intermediate. *J. Am. Chem. Soc.* **2025**, *147*, 10298–10308.

[35] N. Meng, J. Shao, H. Li, Y. Wang, X. Fu, C. Liu, Y. Yu, B. Zhang, Electrosynthesis of formamide from methanol and ammonia under ambient conditions. *Nat. Commun.* **2022**, *13*, 5452.

Response to comments from the reviewer # 2:

General Comments. In this work, the authors report a I_2-mediated DMF electrolysis and achieve a great electrochemical performance. However, the reviewer considers a major revision is necessary for publishing in Nature Communications.

Response to Reviewer 2:

We would like to express our sincere thanks to this reviewer for her/his valuable comments and constructive suggestions, which are helpful to significantly improve the manuscript. We have carefully revised the manuscript according to your comments. The following is the response to each comment.

Comment 1.

The catalyst characterization should be supplemented. The XRD patters only shows a characteristic peak of GC, which may be attributed the low loading of PyCoPc. ICP-OES or ICP-MS of PyCoPc/GC and XRD of pure PyCoPc are necessary. Besides, the SEM images with higher magnification and high-resolution TEM should be added to reveal the surface morphology and structure. Furthermore, the AC-STEM is also suggested to conduct.

Response: Thanks for the comment.

1. We used ICP-OES to determine the Co content of GF-based electrode. As shown in Supplementary Table 2, the Co content of PyCoPc/GF (0.0046%) is higher than that in CoPc/GF (0.0033%), due to the pyrrolidone group substitution facilitating anchoring on the GF substrate.

Changes made in Revised Manuscript:

Page 8, lines 17-19.

The Co content of PyCoPc/GF (0.0046%) is higher than that in CoPc/GF (0.0033%) (Supplementary Table 2).

Changes made in Revised Supporting Information:

Page 76.

Supplementary Table 2 Co contents in GF-based electrodes as determined by ICP-OES.

Samples	Co contents
CoPc/GF	0.0033%
PyCoPc/GF	0.0046%

2. We have provided the XRD pattern of pure PyCoPc in Supplementary Fig. 12. As shown in Supplementary Fig. 12, the peak at 27° can be attributed to the π-π stacking of PyCoPc molecules (*J. Mater. Chem.* 2011, 21, 18552-18559; *J. Mater. Chem.* 2012, 22, 15695-15701.). Compared to this, PyCoPc/GF displays only characteristic peaks of graphitic carbon (Supplementary Fig. 11), providing additional evidence against molecular aggregation.

Changes made in Revised Manuscript:

Page 8, lines 11.

Furthermore, the X-ray diffraction (XRD) patterns of single-molecule-integrated electrodes display only characteristic peaks of graphitic carbon (Supplementary Fig. 11), unlike those of PyCoPc (Supplementary Fig. 12), providing additional evidence against molecule aggregation.

Changes made in Revised Supporting Information:

Page 20

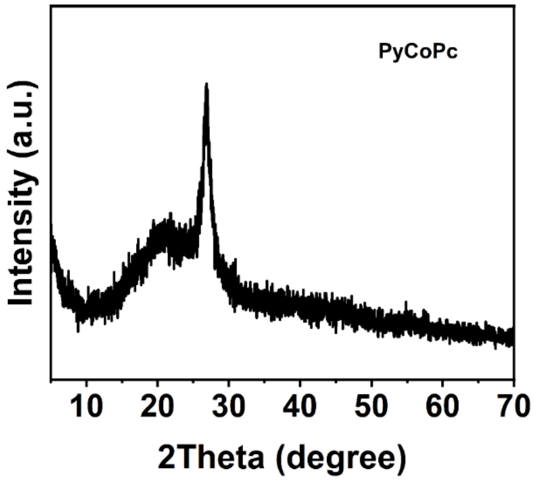

Supplementary Fig. 12 XRD patterns of PyCoPc.

It should be noted that the peak at 27° can be attributed to the π-π stacking of PyCoPc molecules. [14, 15]

Page 86, lines 5-11.

[14] J. Kan, Y. Chen, J. Gao, L. Wan, T. Lei, P. Ma, J. Jiang, Synthesis, self-assembly, and semiconducting properties of phenanthroline-fused phthalocyanine derivatives. *J. Mater. Chem.* **2012**, *22*, 15695.

[15] P. Ma, J. Kan, Y. Zhang, C. Hang, Y. Bian, Y. Chen, N. Kobayshi, J. Jiang, The first solution-processable n-type phthalocyaninato copper semiconductor: tuning the semiconducting nature via peripheral electron-withdrawing octyloxycarbonyl substituents. *J. Mater. Chem.* **2011**, *21*, 18552.

3. As shown in Supplementary Fig. 7, high-magnification SEM images reveal that the PyCoPc/GF surface exhibits a smooth fibrous morphology devoid of discernible particulate aggregates. To further characterize the nanostructure of PyCoPc/GF, the integrated electrode was subjected to ball milling using a ball mill (Supplementary Fig. 8). The powder collected from the milling jar was then characterized by TEM and AC-HAADF-STEM. Collectively, the SEM (Supplementary Fig. 8), TEM (Supplementary Fig. 9), and AC-HAADF-STEM (Supplementary Fig. 10a) results provide conclusive evidence for the single-molecular dispersion of PyCoPc on the GF substrate, attributable to enhanced anchoring facilitated by the pyrrolidone group substitution. In contrast, the lower solubility of CoPc resulted in partial single-molecular dispersion with inevitable aggregation (Supplementary Fig. 10b).

Changes made in Revised Manuscript:

Page 8, lines 9-17.

To better characterize the nanostructure of PyCoPc/GF, the integrated electrode was ball-milled (Supplementary Fig. 8) prior to transmission electron microscopy (TEM) and spherical aberration-corrected high-angle annular dark-field scanning transmission electron microscopy (AC-HAADF-STEM) characterization. TEM and AC-HAADF-STEM analyses further confirm the single-molecular dispersion of PyCoPc on the GF surface (Supplementary Figs. 9 and 10a), attributable to enhanced anchoring facilitated by the pyrrolidone group substitution. In contrast, the lower solubility of CoPc resulted in partial single-molecular dispersion with inevitable aggregation (Supplementary Fig. 10b).

Changes made in Revised Supporting Information:

Page 15.

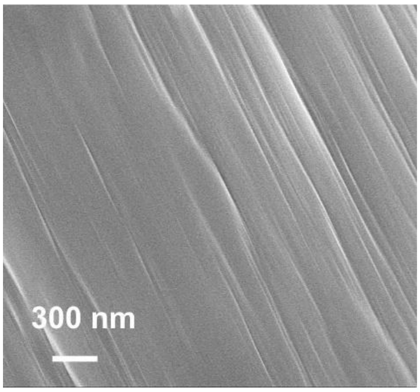

Supplementary Fig. 7 SEM image of PyCoPc/GF with high magnification.

Page 16.

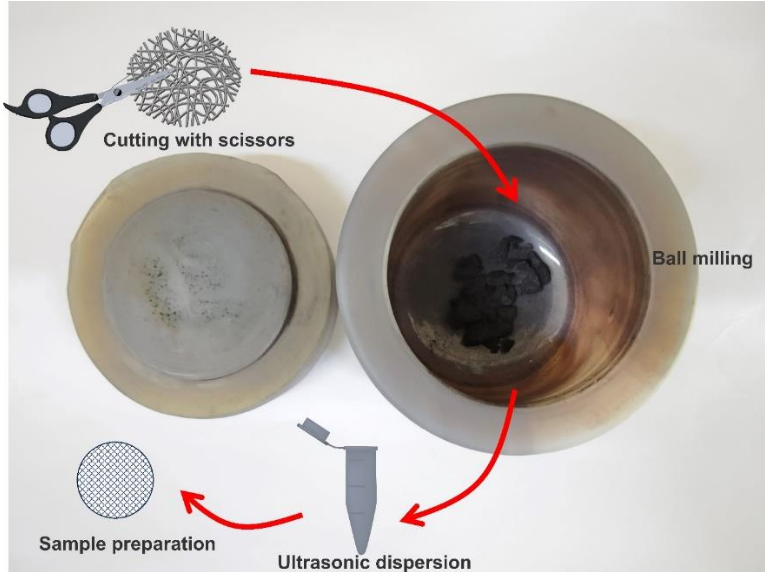

Supplementary Fig. 8 Schematic of ball milling-based sample preparation.

Page 17.

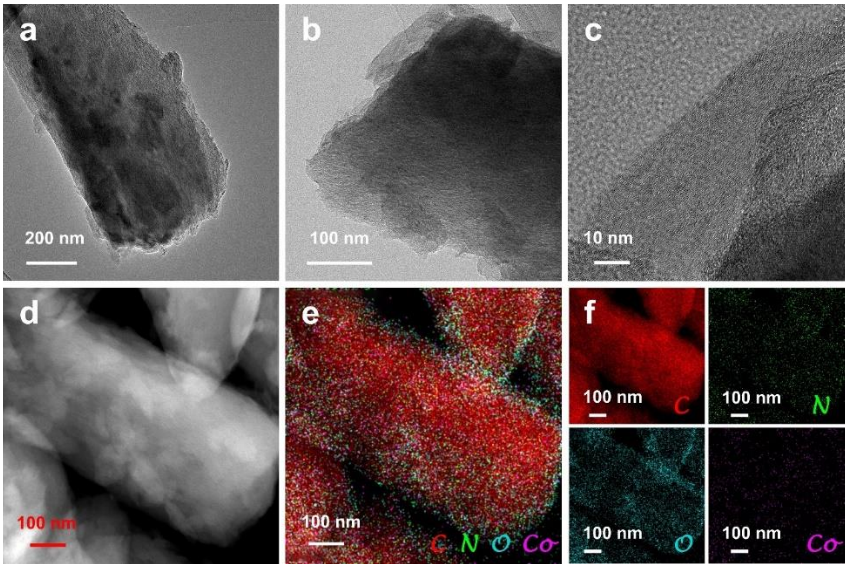

Supplementary Fig. 9 (a, b) TEM images; (c) HRTEM image; (d) HADDF-STEM image and (e, f) corresponding EDX elemental mapping images of PyCoPc/GF after ball milling.

Page 18.

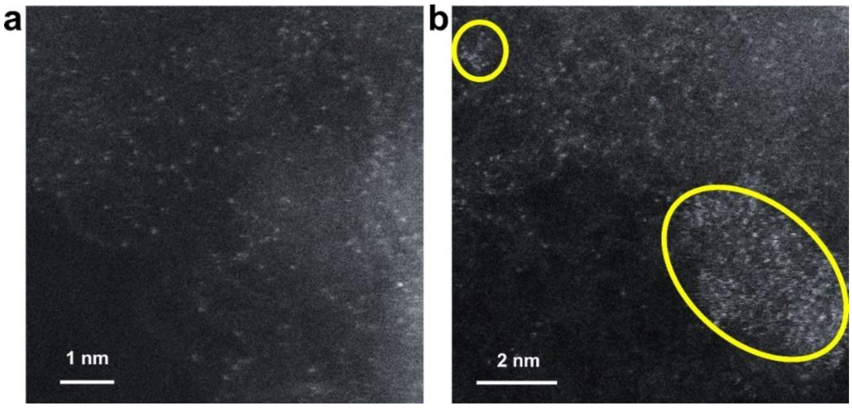

Supplementary Fig. 10 AC-HADDF-STEM images of (a) PyCoPc/GF, (b) CoPc/GF.

<u>Comment 2.</u>

For the electrochemical study, the detailed LSV curves under different conditions should be added (various electrodes, and KI concentrations). Besides, is O_2 or I_2 generated during the reaction? Furthermore, the usage of BPM shows a slight increase of FE_{DMF} while it may also raise the cell voltage. The authors consider that the enhancement originates from cross-over of anodic reactants, while the phenomenon of cross-over is not noticeable in H cell. So, this opinion is unconvincing and the authors should consider carefully the enhancement after using BPM. Furthermore, there is no obvious decay after 150 hours

Why not continue testing? Does the amount of KI change after the reaction? The concentration of KI after the reaction should also be quantified to evaluate the potential degradation. The post-reaction characterization of the electrode should be analyzed deeply.

Response: Thanks for the comment.

1. We have supplemented the manuscript with LSV curves obtained under varying KI concentrations (Supplementary Fig. 19) and for different catalysts (Supplementary Fig. 25). As shown in Supplementary Fig. 19, the addition of KI significantly increased the current and induced a negative shift in the onset potential, from approximately 0.42 V without KI to about 0.30 V at a KI concentration of 100 mM. Furthermore, Supplementary Fig. 25 reveals that PyCoPc/GF exhibits a significantly higher current density than both CoPc/GF and GF, and a much lower onset potential (0.30 V) compared to 0.44 V for CoPc/GF and 0.53 V for GF, demonstrating its superior catalytic activity.

Changes made in Revised Manuscript:

Page 9, lines 24-27.

Linear sweep voltammetry (LSV) was initially employed to evaluate the effect of KI on the catalytic reaction. As shown in Supplementary Fig. 19, the addition of KI significantly increased the current and induced a negative shift in the onset potential, from approximately 0.42 V without KI to about 0.30 V at a KI concentration of 100 mM.

Page 11, lines 14-16.

PyCoPc/GF exhibits a significantly higher current density than both CoPc/GF and GF, and a much lower onset potential of 0.30 V compared to CoPc/GF (0.44 V) and GF (0.53 V), demonstrating its superior catalytic activity (Supplementary Fig. 25).

Changes made in Revised Supporting Information:

Page 27

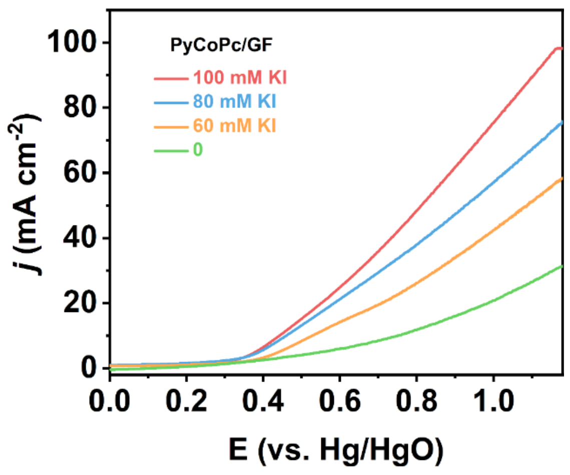

Supplementary Fig. 19 LSV curves of PyCoPc/GF with different concentrations of KI.

Page 33

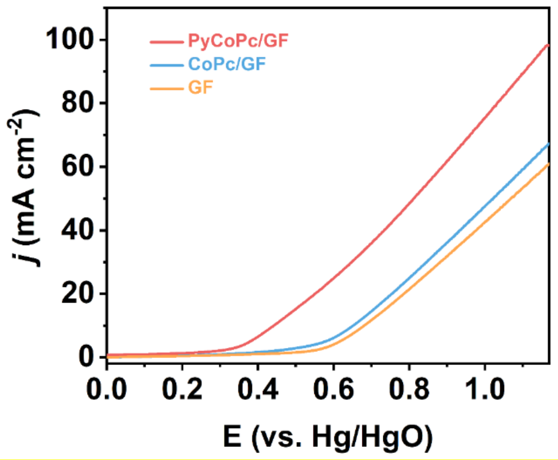

Supplementary Fig. 25 LSV curves of PyCoPc/GF, CoPc/GF and GF in 0.7 M K_2CO_3 with 100 mM KI.

2. During the reaction, I_2 is generated while the OER is suppressed in the presence of high concentrations of methanol and DMA, resulting in no detectable bubble formation.

3. Thank you for your insightful comments. While organic crossover may be negligible during short-term testing, it significantly compromises both cathode and anode reactions during prolonged operation, particularly in tandem configurations. The bipolar membrane (BM) effectively suppresses this crossover. Furthermore, as described in the reaction mechanism (Fig. 5a), alkaline conditions promote DMF formation. Critically, provides additional OH^- to the anode by electrochemically dissociating water at its intermediate interfacial layer, thereby promoting DMF

electrosynthesis (*Angew. Chem. Int. Ed.* 2026, 65, e17470; *Appl. Energy* 2025, 394, 126176). Although all three membrane types demonstrated comparable performance in H-cell testing, BM was ultimately selected as optimal for our system due to its combined advantages.

Changes made in Revised Manuscript:

Page 10, line 14 to Page 11, line 7.

As shown in Supplementary Fig. 23, all three membranes exhibited comparable catalytic activity, with the bipolar membrane (BM) achieving a marginally higher FE_{DMF} (91%). BM was selected for subsequent testing due to its dual functionality of providing additional OH^- to the anode through electrochemical water dissociation at the interfacial layer, thereby promoting DMF electrosynthesis, while effectively suppressing organic crossover from the anolyte to the catholyte during prolonged operation to prevent interference with electrode reactions in both compartments.[31, 32]

Page 24, lines 15-21.

[31] W. Zhu, Q.-C. Chen, Y. Chen, J. E. Huang, G. Su, H. Liu, W. Ni, Y. Chen, J. Yu, B. Peng, J. Li, S. Lee, S. Hao, Y. Shen, H. Ze, B. Zhou, X.-Y. Li, Y. Ji, S. Yang, C. Tian, Y. Liang, K. Xie, E. H. Sargent, High-concentration alcohol generation in bipolar membrane CO electrolyzer. *Angew. Chem. Int. Ed.* **2026**, *65*, e17470.

[32] F. Meng, D. Cheng, M. Lin, K. Zuo, Z. B. Zhang, Review on crossover minimization and catalytic layer-promoted water dissociation in bipolar membranes. *Appl. Energy* **2025**, *394*, 126176.

4. In this study, a 150 h stability test conducted in an H-type electrolytic cell demonstrates robust operational stability. While longer-term stability evaluation employing advanced systems such as flow cells will be systematically pursued in future work to further validate industrial applicability.

5. Thanks for the comment. During extended stability testing, the electrolyte was replenished every 10 h (see Section *1.3.1, Electrochemical Measurements*), maintaining the mediator concentration well above the reaction stoichiometry. Throughout the reaction, a portion of the generated I_2 participated in the reaction, while the excess I_2 exists in equilibrium with I^- in the electrolyte via the reaction I_3^-

$\rightleftharpoons I_2 + I^-$. Both forms are considered part of the effective iodine content. Post-reaction, I_2 and I_3^- were quantitatively reduced to I^- using Na_2SO_3, and the I^- concentration was determined by UV-Vis spectrophotometry. Analysis revealed an active iodine content of 92 mM after reaction (Supplementary Fig. 34), compared to the initial KI concentration of 100 mM, demonstrating the reversibility and stability of the iodine-based mediator system under operational conditions.

Changes made in Revised Supporting Information:

Page 42.

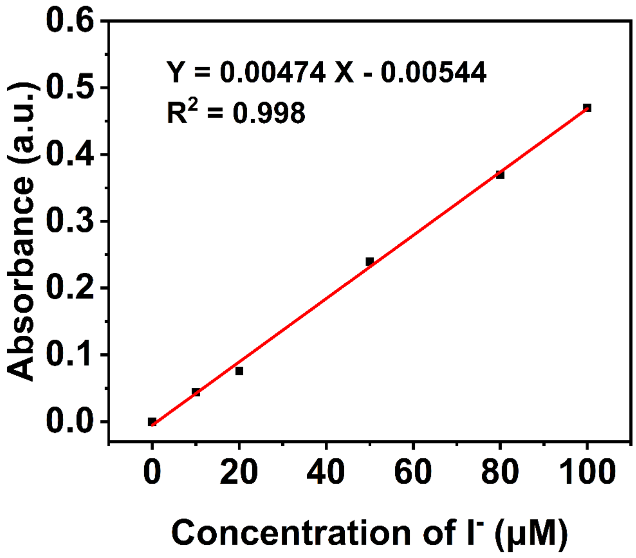

Supplementary Fig. 34 UV-Vis spectrophotometric calibration curve for I^- quantification.

During extended stability testing, the electrolyte was replenished every 10 h, maintaining the mediator concentration well above the reaction stoichiometry. Throughout the reaction, a portion of the generated I_2 participated in the reaction, while the excess I_2 exists in equilibrium with I^- in the electrolyte via the reaction $I_3^- \rightleftharpoons I_2 + I^-$. Both forms are considered part of the effective iodine content. Post-reaction, I_2 and I_3^- were quantitatively reduced to I^- using Na_2SO_3, and the I^- concentration was determined by UV-Vis spectrophotometry.

Changes made in Revised Manuscript:

Page 11 line 30 to Page 12 line 3.

Critically, quantification of active iodine species post-reaction revealed 92 mM residual

concentration (Supplementary Fig. 34), representing 92% retention compared to the initial KI concentration (100 mM), confirming mediator reversibility and system stability.

6. We further characterized the post-reaction electrode using UV-Vis spectroscopy, XPS and Raman spectroscopy, which further confirmed the preservation of the catalyst structure. Prior to characterization, the post-reaction samples were sequentially rinsed with deionized water and methanol, followed by vacuum drying to remove surface contaminants. The UV-Vis spectrum of the post-reaction PyCoPc/GF retained the characteristic phthalocyanine absorption peak at 710 nm (Supplementary Fig. 29). Similarly, the Raman spectrum preserved the characteristic phthalocyanine peaks (Supplementary Fig. 30). Furthermore, the XPS spectrum confirmed the Co^{2+} peak position in the Co $2p_{3/2}$ region remained at approximately 780.4 eV, exhibiting no significant shift (Supplementary Fig. 31).

Changes made in Revised Supporting Information:

Page 4, lines 15-19

Upon completion of the test, to eliminate residual electrolytes and reaction products that could interfere with post-test electrode characterization, the electrodes were sequentially subjected to copious rinsing with deionized water, followed by ultrasonication in a beaker for 10 minutes, and then rinsed thoroughly with methanol three times. Finally, the electrodes were dried overnight in a vacuum oven at 60 °C under continuous vacuum.

Page 37.

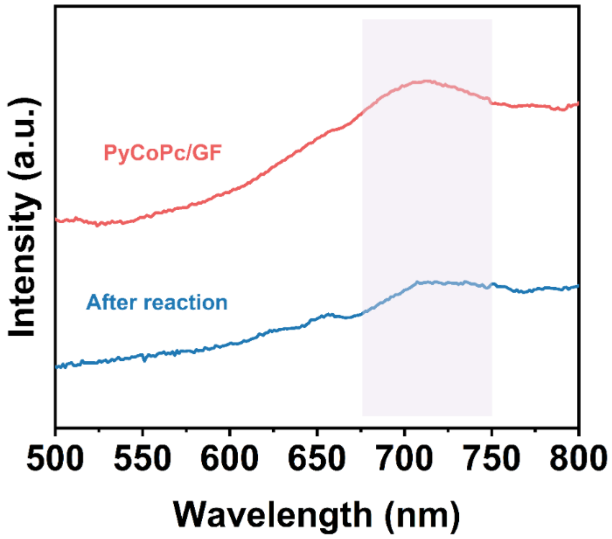

Supplementary Fig. 29 UV-Vis spectra of PyCoPc/GF before and after reaction.

Prior to characterization of the post-reaction catalyst, samples were sequentially rinsed with deionized water and methanol, followed by vacuum drying to remove surface contaminants. Detailed procedures are documented in the Experimental Section.

The Py-CoPc/GF electrode was subjected to pulverization and dispersion in methanol solution for characterization. After reaction, the characteristic phthalocyanine peak remained detectable at approximately 710 nm.

Page 38.

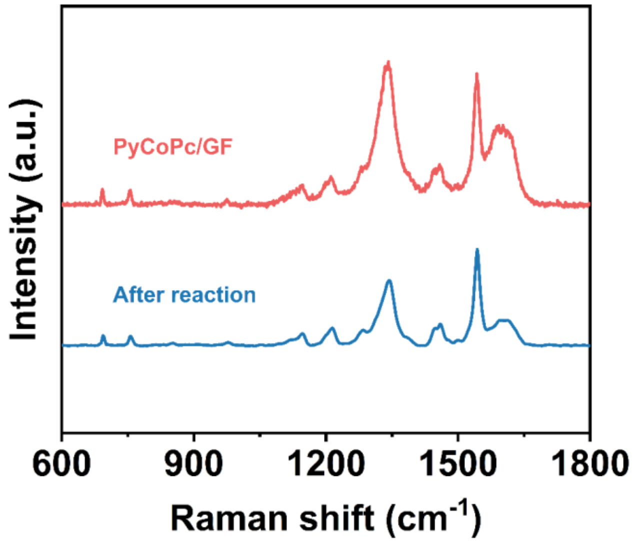

Supplementary Fig. 30 Raman spectra of PyCoPc/GF before and after reaction.

Page 39.

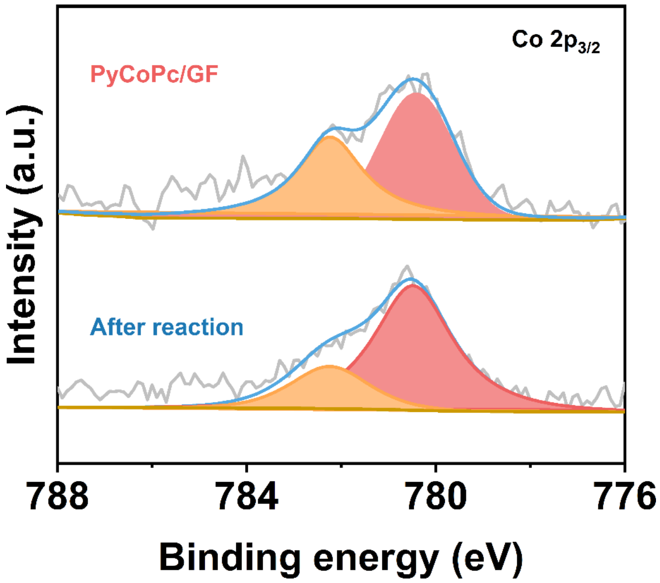

Supplementary Fig. 31 Co 2p XPS spectra of PyCoPc/GF before and after reaction.

After the reaction, the Co 2p$_{3/2}$ XPS spectrum of PyCoPc/GF showed that the Co^{2+} peak was observed at 780.4 eV with no significant shift.

Changes made in Revised Manuscript:

Page 11, line 30.

Furthermore, the long-term stability test for 150 h demonstrates that PyCoPc/GF exhibits a consistent catalytic activity, capable of well maintaining the high FE_{DMF} (~90%), productivity (839 μmol h^{-1} cm^{-2}) for DMF production (Fig. 3e and Supplementary Figs. 28-33).

Comment 3.

The assignment of the peaks near 1650 cm^{-1} may be attributed to interfacial water and the authors should analyze the results of in situ ATR-IR carefully. In addition, the poor yield of DMF from electrooxidation of TMDM is abnormal and should be carefully examined. What are the other products in this reaction. In the Figure 4e, the I_2 and *CH_2O species may generate simultaneously and the authors should consider carefully. It is recommended that authors carefully describe the reaction mechanism in the Introduction section to help readers understand.

Response: Thanks for the comment.

1. During the *in situ* ATR-IR testing, we rigorously subtracted the solvent peaks to eliminate their influence. Based on our proposed mechanism, an equilibrium is maintained between water elimination and addition. We supplemented the analysis with the *in situ* ATR-IR spectra obtained with and without KI addition (Supplementary Fig. 36), which revealed distinct changes in the formation of the *CH_2O and DMF. Furthermore, based on references (Chem 2024, 10, 2437-2449; Green Chem. 2023, 25, 5936-5944), we assigned the peak at 1662 cm^{-1} to *CH_2O and the peak at 1623 cm^{-1} to DMF.

Changes made in Revised Manuscript:

Page 13, lines 9-16.

As shown in Supplementary Fig. 36, the peak at 1662 cm^{-1} corresponds to *CH_2O derived from methanol partial oxidation, which subsequently reacts with DMA in 0.7 M K_2CO_3 to form TMDM rapidly. Compared to the KI-free system, KI addition significantly

enhances *CH_2O intermediate generation and facilitates rapid DMF production via I_2-mediated electro-oxidative decomposition of TMDM, as evidenced by the characteristic DMF peak at 1623 cm^{-1}.[6, 8]

Changes made in Revised Supporting Information:

Page 44

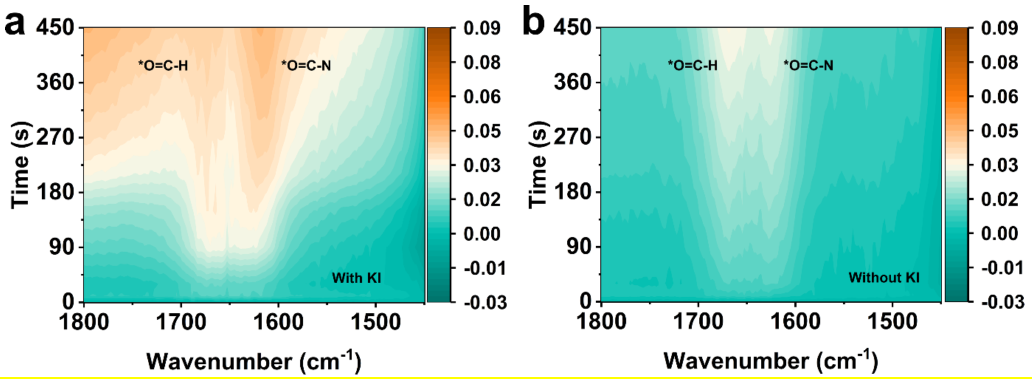

Supplementary Fig. 36 *In situ* ATR-IR spectrum for the DMF electrosynthesis over PyCoPc/GF in 0.7 M K_2CO_3 (a) with 100 mM KI, (b) without KI.

2. Direct electrolysis of TMDM yielded an S_{DMF}/S_{C12} ratio of only 0.49, whereas iodine-mediated electrolysis significantly enhanced this ratio to 2.34. This substantial improvement is attributed to the introduction of iodine, which facilitates the formation of iodinated intermediates that optimize the reaction pathway and promote DMF generation. In contrast, direct electro-oxidative dehydrogenation of TMDM proved inherently difficult, which aligns with DFT calculations (Fig. 5b). Besides, our prior work demonstrates that promoting the conversion of TMDM to DMF requires carefully designed catalysts (*Appl. Catal. B-Environ.* 2026, 382, 125989).

Changes made in Revised Manuscript:

Page 13, lines 23-25.

In contrast, direct electrolysis of TMDM without I_2 yielded an S_{DMF}/S_{C12} ratio of 0.49, which is attributed to the difficulty of the direct electro-oxidative dehydrogenation of TMDM.

Changes made in Revised Supporting Information:

Page 22, lines 34-37.

[12] Y. He, X. Xiao, X. Li, D.-D. Ma, Q.-L. Zhu, Q. Xu, Unlocking high-efficiency

electrosynthesis of *N,N*-dimethylformamide through the synergy of three-dimensional ordered macroporous superstructure and implanted nano-tentacles. *Appl. Catal. B-Environ.* **2026**, *382*, 125989.

3. We conducted GC (Supplementary Fig. 20), GC-MS (Supplementary Fig. 21), and NMR (Supplementary Fig. 22) analyses to characterize byproducts. These analyses identified formate and TMDM as the primary side products, with concomitant I_2 generation, collectively confirming that *in situ* electrogenerated I_2 enables highly selective electrosynthesis of DMF. We have reemphasized this point in the Revised Manuscript.

Changes made in Revised Manuscript:

Page 9, lines 2-4.

Further analyses of the post-electrolysis electrolyte by GC, gas chromatography-mass spectrometry (GC-MS), and NMR confirm that *in situ* electrogenerated I_2 facilitates highly selective electrosynthesis of DMF (Fig. 3b and Supplementary Figs. 20-22).

4. As noted by the Reviewer, the I_2 and $*CH_2O$ species may generate simultaneously. Accordingly, we have revised Fig. 5a to reflect this mechanistic insight.

Changes made in Revised Manuscript:

Page 14, Fig. 5a.

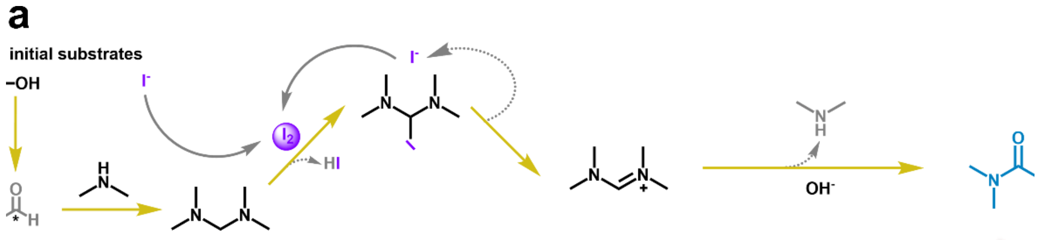

Page 15, line 7.

It should be noted that I_2 and $*CH_2O$ species may generate simultaneously.

5. We appreciate the suggestion and have presented a detailed discussion of the I_2-mediated promoted DMF electrosynthesis mechanism in Mechanistic Study. To enhance clarity regarding the reaction pathway, we have added a schematic diagram of the DMF electrosynthesis mechanism from methanol and DMA in the Introduction section (*Appl. Catal. B-Environ.* 2026, 382, 125989; *Nat. Commun.* 2025, 16, 3564.).

Changes made in Revised Supporting Information:

Page 10, Supplementary Fig. 2.

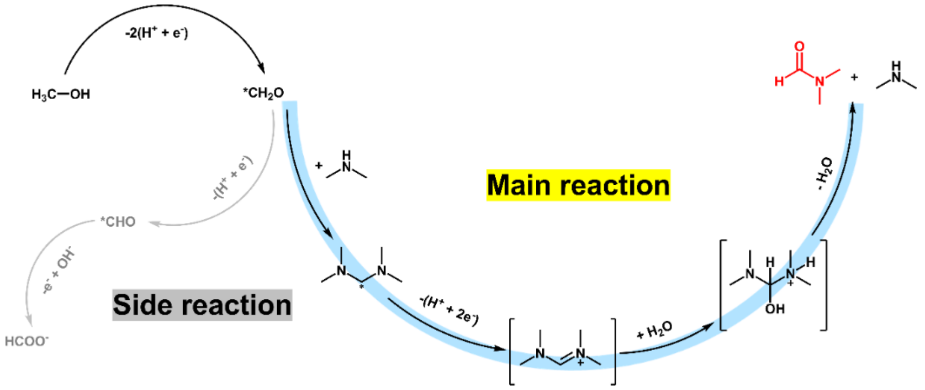

Supplementary Fig. 2 Proposed reaction mechanism of electrosynthesis of DMF from methanol and DMA.

It should be noted that formaldehyde and DMA can rapidly react to form TMDM,[12, 13] making direct quantification of formaldehyde difficult, especially in the presence of high DMA concentrations.

Page 85, line 29 to Page 86 line 4.

[12] G. P. Mrug, S. P. Bondarenko, V. P. Khilya, M. S. Frasinyuk, Synthesis and aminomethylation of 7-hydroxy-5-methoxyisoflavones. *Chem. Nat. Compd.* **2013**, *49*, 235-241.

[13] H. Heaney, G. Papageorgiou, R. F. Wilkins, The generation of iminium ions using chlorosilanes and their reactions with electron rich aromatic heterocycles. *Tetrahedron* **1997**, *53*, 2941-2958.

Changes made in Revised Manuscript:

Page 4, lines 1-2.

…in the electrochemical synthesis of DMF (Supplementary Fig. 2).[11, 12]

Page 22, lines 34-37.

[12] Y. He, X. Xiao, X. Li, D.-D. Ma, Q.-L. Zhu, Q. X., Unlocking high-efficiency electrosynthesis of *N,N*-dimethylformamide through the synergy of three-dimensional ordered macroporous superstructure and implanted nano-tentacles. *Appl. Catal. B-Environ.* **2026**, *382*, 125989.

Comment 4.

The authors should consider whether a carbonate-mediated pathway could be involved in the reaction? The isotopic labeling experiment may be beneficial for mechanism studies. Similarly, is this I_2-mediated strategy universally applicable? For instance, could this strategy be extended to the electrooxidation of methanol (MeOH) and other nitrogenous species? Furthermore, the technoeconomic assessment is useful to evaluate economic viability.

Response: Thanks for the comment.

1. We further tested the experiment with ^{13}C-labeled K_2CO_3. GC-MS results indicate that CO_3^{2-} did not participate in the reaction.

Changes made in Revised Supporting Information:

Page 45.

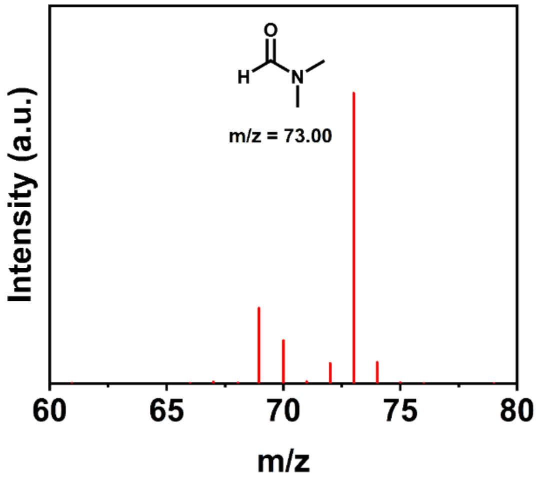

Supplementary Fig. 37 GC-MS spectrum of DMF using ^{13}C-labeled K_2CO_3.

Changes made in Revised Manuscript:

Page 13, lines 13-15.

Furthermore, experiments with ^{13}C-labeled K_2CO_3 confirmed that CO_3^{2-} in the electrolyte were not involved in the reaction (Supplementary Fig. 37).

2. We propose that the key step in the I_2-mediated strategy involves the generation of an iodinated intermediate. Building upon our earlier work demonstrating the synthesis of N-C-N bonded compounds like *N,N,N',N'*-tetraethylmethanediamine and dimorpholinomethane through amine substrate scope expansion (*Nat. Commun.* 2025, 16, 3564). Building upon this foundation, we applied the I_2-mediated strategy and the

productivity of *N,N*-diethylmethylamine and *N*-formylmorpholine were determined to 383 and 145 μmol h^{-1} cm^{-2}, respectively, demonstrating the versatility of this approach.

Changes made in Revised Supporting Information:

Page 43.

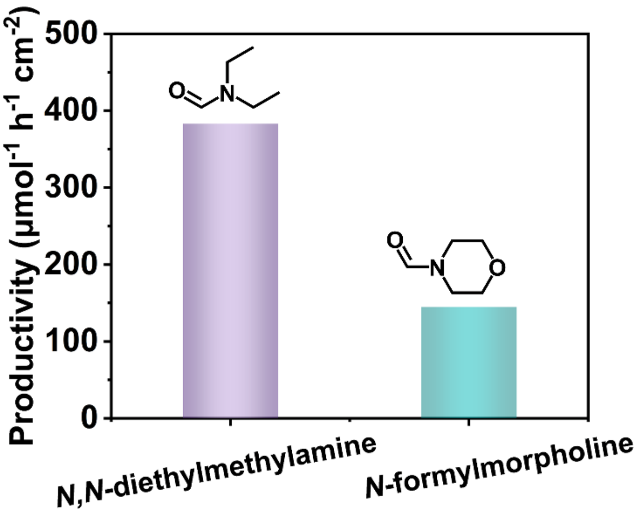

Supplementary Fig. 35 Substrate scope for the I_2-mediated strategy.

Under the optimal conditions for electrosynthesis in DMF, dimethylamine was replaced with equivalent volumes of 40 wt% diethylamine and morpholine solutions.

Changes made in Revised Manuscript:

Page 11, lines 3-6.

To further demonstrate the versatility of the I_2-mediated strategy, the substrate scope was expanded to additional amines, affording *N,N*-diethylmethylamine and *N*-formylmorpholine with productivity of 383 and 145 μmol h^{-1} cm^{-2}, respectively (Supplementary Fig. 35).

3. Based on a modified version of the previously reported model (*Nat. Synth.* 2025, 4, 1598–1609; *Nat. Commun.* 2022, 13, 5452.), we conducted a preliminary technical-economic analysis (TEA) for DMF production. The detailed calculation methodology is provided in Supplementary Note 1. Targeting a daily production capacity of 1 tone (t) of DMF and incorporating several reasonable assumptions, we defined:

   The total cost = Capital cost + Operating cost + Material cost (Supplementary Fig. 67)

   Daily profit = income – the total cost

This analysis yielded a daily profit of 688.6 USD t^{-1} day^{-1}, indicating its potential for practical implementation.

Changes made in Revised Manuscript:

Page 19, lines 15-18.

Technoeconomic analysis (TEA, Supplementary Note 1 and Supplementary Figs. 67-69) shows that the daily profit of DMF from this electrosynthesis can reach 688.6 USD t^{-1} day^{-1}, highlighting its potential for practical implementation.

Changes made in Revised Supporting Information:

Page 79-84.

4. Supplementary Note 1

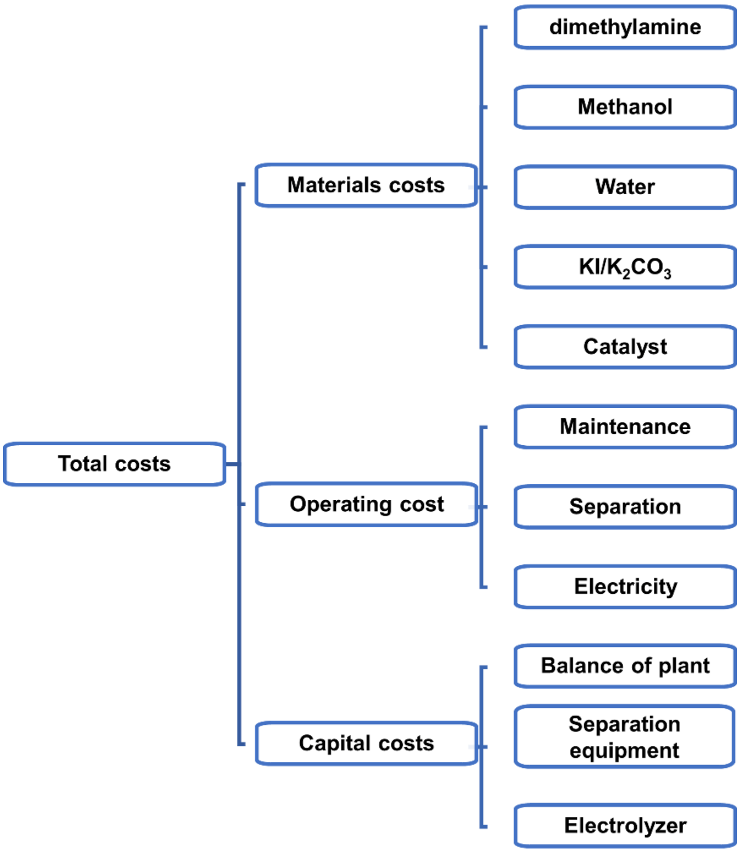

Supplementary Fig. 67 TEA model used for the production of DMF.

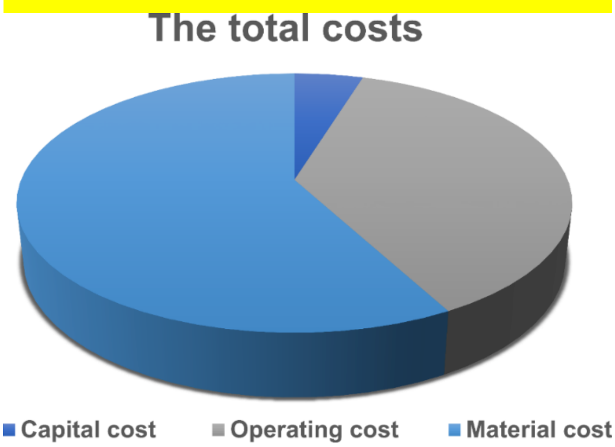

Supplementary Fig. 68 Total costs assessment in the TEA of DMF production.

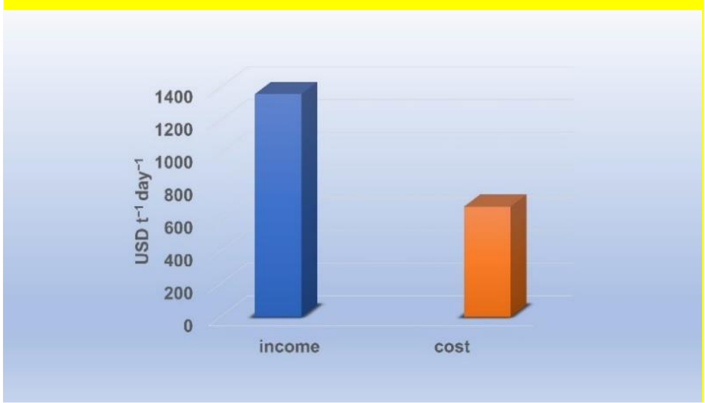

Supplementary Fig. 69 Income and cost assessment in the TEA of DMF production.

Techno-economic analysis (TEA) of the DMF electrosynthesis

A preliminary techno-economic analysis (TEA) of the DMF production was conducted based on a previously reported model with a modification.[19, 20] Some reasonable assumptions are made to produce 1 ton (t) of DMF per day.

Assumptions:

(1) In this work, the FE of DMF is approximately 73%; the cell voltage is approximately 1.0 V, and the operating current density is −200 mA cm^{-2} (Fig. 7).

(2) The lifetime of the plant is assumed to be 10 years, and the working time is set as 350 days per year.

(3) The cost of Balance of Plant is assumed to be 50% of the electrolyzer cost.

(4) The separation equipment cost is set as 150% of the electrolyzer cost.

(5) The electricity price is assumed to be 0.05 USD kWh^{-1}.

(6) The maintenance cost is set as 2.5% of the capital cost.

(7) The separation cost is set as 150% of the electricity cost.

(8) The market price of MeOH (99.9%, Shandong Alliance Chemical Group Co., Ltd. China) is approximately 264 USD t^{-1}, the market price of dimethylamine (40 wt%, Huainan Kedi Chemical Technology Co., Ltd. China) is approximately 215 USD t^{-1}, and the market price of DMF (99.9%, Shandong Jinyueyuan New Materials Co., Ltd. China) is approximately 1362 USD t^{-1}.

To produce 1 t of DMF, some basic parameters can be calculated:

(1) The required total charge was calculated as follows. The FE of DMF is 73% (1.0 V, 200 mA cm^{-2}) obtained from the electrolyzer.

Q = (Mass of DMF × F × n) / (Molar mass of DMF × FE of DMF)

$= 7.23 \times 10^{9}$ C

(2) The total current required was calculated as follows.

$I = Q/t$

= 83681 A

(3) The required area of the electrolyzer was calculated as follows.

Electrolyzer area = I / current density

= 83681 A / 0.2 A cm^{-2}

= 41.84 m^2

(4) The power required was calculated as follows.

Power = 1.0 V × 83681 A = 83.68 kW

(5) The electrolyzer cost per area was calculated as follows.

Electrolyzer cost per area = 450 USD kW^{-1} × 1.0 V × 0.2 A cm^{-2}

= 900 USD m^{-2}

Capital cost:

(1) Electrolyzer

Electrolyzer cost = 41.84 m^2 × 900 USD m^{-2} = 37656 USD

(2) Separation Equipment

Separation equipment cost = 37656 USD × 150% = 56484 USD

(3) Balance of plant

Cost of balance of plant = 37656 USD × 50% = 18828 USD

Capital cost = (cost of electrolyzer + cost of balance of plant + cost of separation equipment) / (Lifetime of plant × m_{DMF} per day)

= (37656 USD + 56484 USD +18828 USD) / (10 years × 350 days × 1 t)

= 32 USD t^{-1} day^{-1}

Operating cost:

(1) Electricity

Electricity cost = 83.68 kW × 24 h × 0.05 USD kWh^{-1}

= 100.4 USD t^{-1} day^{-1}

(2) Maintenance

Maintenance cost = 32 USD t^{-1} day^{-1} × 2.5% = 0.8 USD t^{-1} day^{-1}

(3) Separation

Separation cost = 100.4 USD t^{-1} day^{-1} × 150% = 150.6 USD t^{-1} day^{-1}

Operating cost = Electricity cost + Maintenance cost + Separation cost

= 100.4 USD t^{-1} day^{-1} + 0.8 USD t^{-1} day^{-1} + 150.6 USD t^{-1} day^{-1}

= 251.8 USD t^{-1} day^{-1}

Material cost:

(1) MeOH

To produce 1 t of DMF per day, 0.44 t of MeOH is consumed.

MeOH cost = 0.44 t MeOH × 264 USD t^{-1} = 116.2 USD t^{-1} day^{-1}

(2) Dimethylamine

To produce 1 t of DMF per day, 1 t of dimethylamine is consumed.

Dimethylamine cost = 1 t of dimethylamine × 215 USD t^{-1} = 215 USD t^{-1} day^{-1}

(3) Catalyst

The catalyst PyCoPc loading is 3.83 × 10^{-2} mg cm^{-2} based on the ICP result of Co. The total PyCoPc required is calculated as follows:

Mass of PyCoPc = 3.83 × 10^{-2} mg cm^{-2} × 41.84 m^2 = 0.016 kg_{PyCoPc}

In our case, assuming the PyCoPc lifespan is 24 h, the number of catalyst changes is 1.

The catalyst cost required per day for 1 t of DMF production can be calculated below.

Catalyst cost = 3571 USD kg_{PyCoPc}^{-1} × 0.016 kg_{PyCoPc} × 1 t^{-1} day^{-1}

= 57 USD t^{-1} day^{-1}

(4) Water, K_2CO_3 and KI

Based on the local water price, the water cost is approximately 0.36 USD t^{-1} day^{-1}. The K_2CO_3 cost is set as 200% of the water cost, which is approximately 0.72 USD t^{-1} day^{-1}. The KI cost is set as 100% of the water cost, which is approximately 0.36 USD t^{-1} day^{-1}.

Material cost = MeOH cost + Dimethylamine cost + Catalyst cost + Water cost + K_2CO_3 cost + KI cost

= 116.2 USD t^{-1} day^{-1} + 215 USD t^{-1} day^{-1} + 57 USD t^{-1} day^{-1} + 0.36 USD t^{-1} day^{-1} + 0.72 USD t^{-1} day^{-1} + 0.36 USD t^{-1} day^{-1}

= 389.6 USD t^{-1} day^{-1}

The total cost:

The total cost = Capital cost + Operating cost + Material cost

= 32 USD t^{-1} day^{-1} + 251.8 USD t^{-1} day^{-1} + 389.6 USD t^{-1} day^{-1}

= 673.4 USD t^{-1} day^{-1}

The daily profit:

Daily profit = income – the total cost

= 1362 USD t^{-1} day^{-1} – 673.4 USD t^{-1} day^{-1}

= 688.6 USD t^{-1} day^{-1}

Changes made in Revised Supporting Information:

Page 86, lines 2-7.

[22] J. Li, X. Liu, S.-M. Xu, M. Xu, Y. Wang, Y. Lyu, A.-Z. Li, Y. Wang, X. Wang, T. Zhou, H. Zhou, Y. Peng, X. Li, L. Zheng, H. Duan, Sustainable oxime production via the electrosynthesis of hydroxylamine in a free state. *Nat. Synth.* **2025**, *4*, 1598–1609.

[23] N. Meng, J. Shao, H. Li, Y. Wang, X. Fu, C. Liu, Y. Yu, B. Zhang, Electrosynthesis of formamide from methanol and ammonia under ambient conditions. *Nat. Commun.* **2022**, *13*, 5452.

Comment 5.

The 2.4 section has little relevance to the central theme of this article. The authors should discuss more clearly how this section supports the key I_2-mediated strategy. The –200 mA

in Figure 6c should be changed into –200 mA cm^{-2}.

Response: Thanks for the comment.

1. To enhance the manuscript's content completeness and logical coherence, the manuscript title has been revised in response to reviewer suggestions to "Redox-Mediated Domino Electrosynthesis of *N,N*-Dimethylformamide with Industrial-Relevant Productivity and Modularized Cathodic Integration". Conventional electrosynthesis approaches predominantly focus on optimizing a single half-reaction while neglecting the other, leading to reduced spatiotemporal and energy efficiencies. To address this limitation, this work employs a paired electrosynthesis strategy utilizing a PyCoPc/GF as a bifunctional catalyst, where simultaneous DMF production occurs at the anode alongside electrocatalytic CO_2 reduction to CO at the cathode. Furthermore, we implemented thermocatalytic cascade reactions to upgrade the cathodic CO into high-value amides, ultimately achieving the co-production of amides at both electrodes. Detailing the paired electrosynthesis strategy, serves as a critical complement to the DMF electrosynthesis methodology, effectively demonstrating its applicability and utility within a paired electrolysis framework and highlighting broader application scenarios.

Changes made in Revised Manuscript:

Page 1.

Redox-Mediated Domino Electrosynthesis of *N,N*-Dimethylformamide with Industrial-Relevant Productivity and Modularized Cathodic Integration

Page 15, lines 20-25.

In conventional electrosynthesis, most studies focus solely on one half-reaction while neglecting the other, leading to reduced spatiotemporal and energy efficiencies. The paired electrosynthesis strategy enables the simultaneous generation of high-value-added products at both the cathode and anode, aligning with green principles.[36-38] Furthermore, the products of electrosynthesis can be further upgraded by performing electro-thermal cascade reactions.[39-41]

2. We have revised the value –200 mA in Fig. 7c to –200 mA cm^{-2}.

Changes made in Revised Manuscript:

Page 17, Fig. 7c.

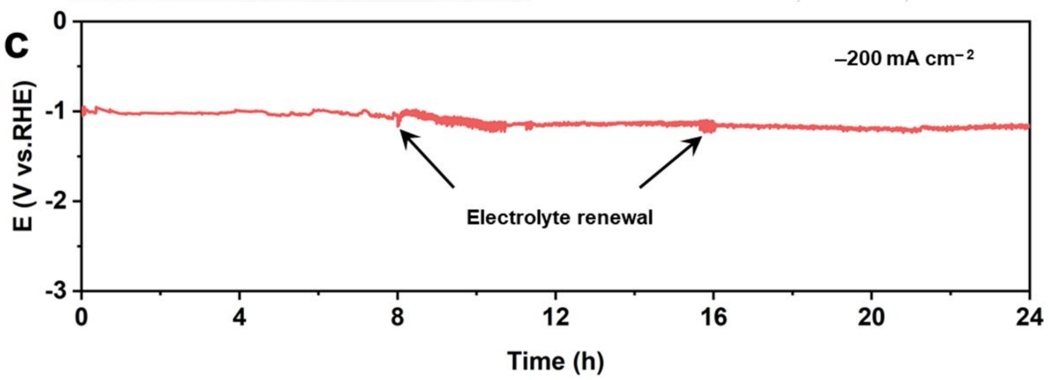

Response to comments from the reviewer # 3:

Response to Reviewer 3:

We would like to express our sincere thanks to this reviewer for her/his valuable comments and constructive suggestions, which are helpful to significantly improve the manuscript. We have carefully revised the manuscript according to your comments. The following is the response to each comment.

Comment 1.

There is a mismatch between the title and the manuscript content: certain key aspects (e.g., the cathodic reaction) are not adequately reflected in the title. Additionally, the description of Ampakine synthesis in the abstract refers to a non-electrochemical process, which is inconsistent with the title's focus on electrosynthesis.

Response: Thanks for the comment. We have revised the title:

Redox-Mediated Domino Electrosynthesis of *N,N*-Dimethylformamide with Industrial-Relevant Productivity and Modularized Cathodic Integration

Changes made in Revised Manuscript:

Page 1

Redox-Mediated Domino Electrosynthesis of *N,N*-Dimethylformamide with Industrial-Relevant Productivity and Modularized Cathodic Integration

Comment 2.

The authors should elaborate on the significance of Ampakine (e.g., its pharmacological value or industrial relevance) in the Introduction section.

Response: Thanks for the suggestion. We synthesized compound A5, an analogue of AMPA receptor positive allosteric modulators structurally inspired by reference compounds such as CX546 and CX614. This compound shows promise for research into psychiatric disorders including Alzheimer's disease and depression (*Neuropharmacology* 2001, 41, 650-663; *Mol. Pharmacol.* 2000, 58, 802-813.). The relevant descriptions in the manuscript have been carefully revised.

Changes made in Revised Manuscript:

Page 2, line 19.

…gram-level amide-containing generic drug production at –200 mA cm^{-2}.

Page 5, line 11.

…producing the amide-containing generic drug (**A5**) with a 86% yield…

Page 17, line 12.

…single-crystal structure of the generic drug **A5** obtained at the cathode.

Page 19, lines 19-24.

To valorize the cathodically generated CO, an electro-thermal cascade upgrading process was implemented for the synthesis of **A5** as a representative example. This compound is an analogue of AMPA receptor positive allosteric modulators, structurally inspired by reference compounds such as CX546 and CX614, shows promise for research into psychiatric disorders like Alzheimer's disease and depression.[46, 47]

Page 20, line 14.

…gram-level of amide-containing generic drug at the cathode…

Page 24, lines 21-26.

[46] A. C. Arai, M. Kessler, G. Rogers, G. Lynch, Effects of the potent ampakine CX614 on hippocampal and recombinant AMPA receptors: interactions with cyclothiazide and GYKI 52466. *Mol. Pharmacol.* ***2000****, 58,* 802-813.

[47] N. Nagarajan, C. Quast, A. R. Boxall, M. Shahid, C. Rosenmund, mechanism and impact of allosteric AMPA receptor modulation by the Ampakine™ CX546. *Neuropharmacology* **2001**, *41,* 650-663.

<u>Comment 3.</u>

X-ray absorption spectroscopy (XAS) data of commercial CoPc should be included for comparison, to clarify the influence of peripheral pyrrolidone substituents on the intrinsic Co-N_4 sites.

Response: Thanks for the suggestion. Under the synergistic action of peripheral pyrrolidone groups and the GF substrate, the characteristic peak position of the intrinsic Co-N_4 coordination site in PyCoPc/GF undergoes a 0.4 eV blue shift from 7716.5 eV in

commercial CoPc to 7716.9 eV.

Changes made in Revised Manuscript:

Page 7, Fig. 2h and 2i.

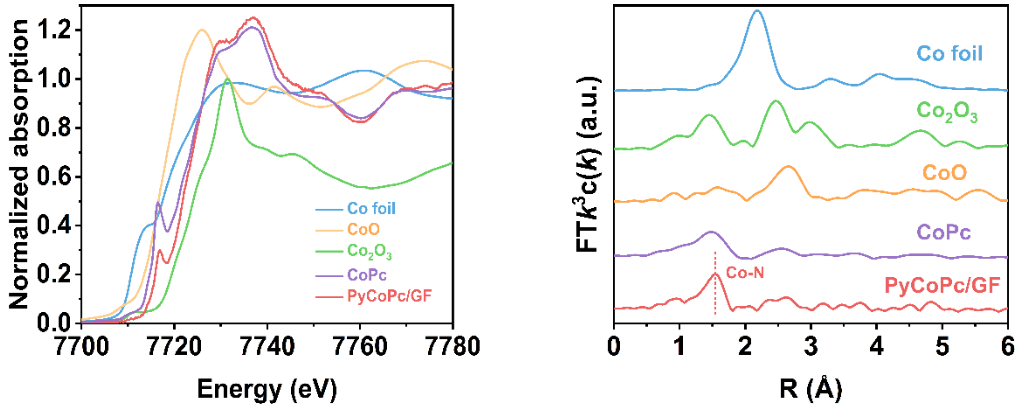

Page 8, lines 5-8.

Comparing to commercial CoPc, the Co-N4 moieties in PyCoPc/GF exhibit a 0.4 eV blue shift from 7716.5 eV to 7716.9 eV due to the synergistic action of peripheral pyrrolidone groups and the GF substrate.

Comment 4.

The current characterization techniques employed (e.g., XRD, XAS, XPS) are insufficient to confirm the single-molecular integrated structure of PyCoPc. The authors are required to employ additional techniques such as HRTEM to verify this—given that CoPc molecules may form a multilayer structure via interfacial π-π stacking interactions. Simultaneously, the uniformity of PyCoPc loading on the GF support should be validated.

Response: Thanks for the comment.

1. As shown in Supplementary Fig. 7, high-magnification SEM images reveal that the PyCoPc/GF surface exhibits a smooth fibrous morphology devoid of discernible particulate aggregates. To further characterize the nanostructure of PyCoPc/GF, the integrated electrode was subjected to ball milling using a ball mill (Supplementary Fig. 8). The powder collected from the milling jar was then characterized by TEM and AC-HAADF-STEM. Collectively, the SEM (Supplementary Fig. 8), TEM (Supplementary Fig. 9), and AC-HAADF-STEM (Supplementary Fig. 10a) results provide conclusive evidence for the single-molecular dispersion of PyCoPc on the GF

substrate, attributable to enhanced anchoring facilitated by the pyrrolidone group substitution. In contrast, the lower solubility of CoPc resulted in partial single-molecular dispersion with inevitable aggregation (Supplementary Fig. 10b).

Changes made in Revised Manuscript:

Page 8, lines 9-17.

To better characterize the nanostructure of PyCoPc/GF, the integrated electrode was ball-milled (Supplementary Fig. 8) prior to transmission electron microscopy (TEM) and spherical aberration-corrected high-angle annular dark-field scanning transmission electron microscopy (AC-HAADF-STEM) characterization. TEM and AC-HAADF-STEM analyses further confirm the single-molecular dispersion of PyCoPc on the GF surface (Supplementary Figs. 9 and 10a), attributable to enhanced anchoring facilitated by the pyrrolidone group substitution. In contrast, the lower solubility of CoPc resulted in partial single-molecular dispersion with inevitable aggregation (Supplementary Fig. 10b).

Changes made in Revised Supporting Information:

Page 15.

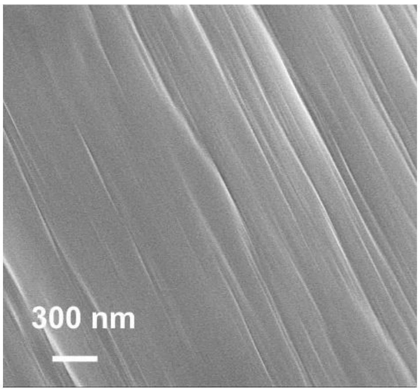

Supplementary Fig. 7 SEM image of PyCoPc/GF with high magnification.

Page 16.

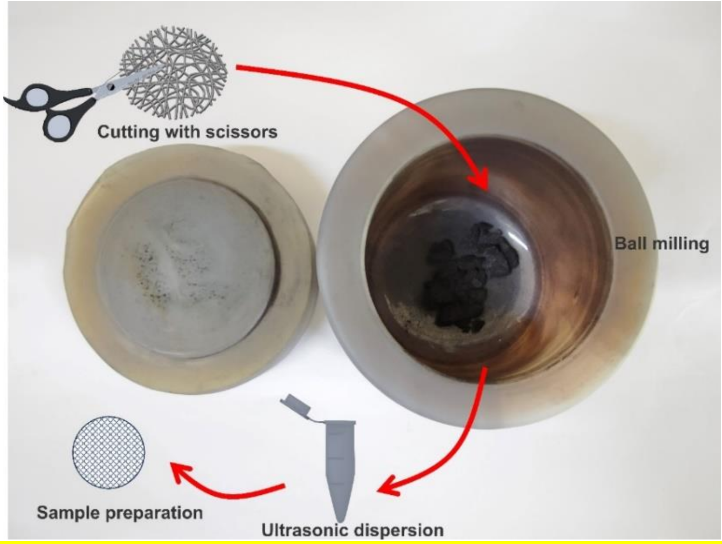

Supplementary Fig. 8 Schematic of ball milling-based sample preparation.

Page 17.

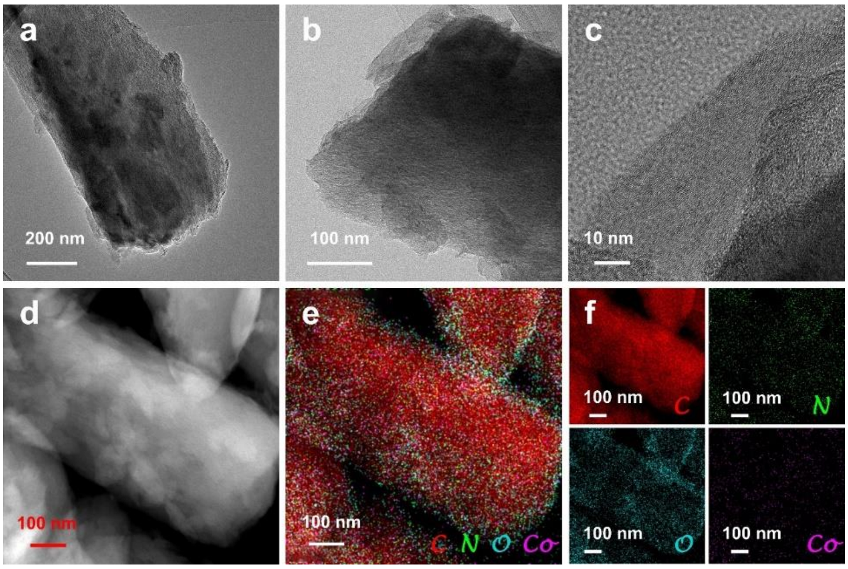

Supplementary Fig. 9 (a, b) TEM images; (c) HRTEM image; (d) HADDF-STEM image and (e, f) corresponding EDX elemental mapping images of PyCoPc/GF after ball milling.

Page 18.

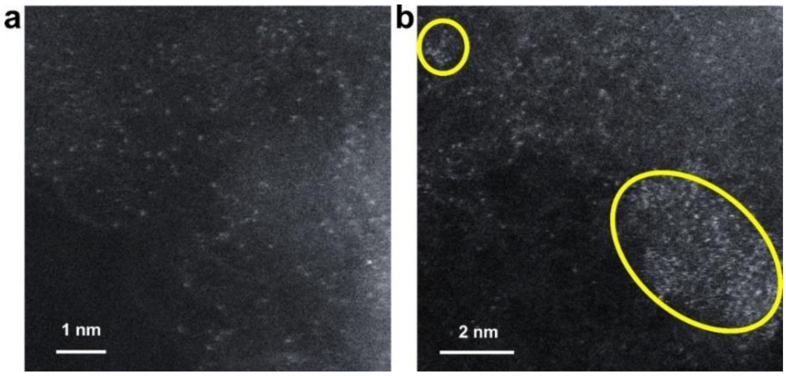

Supplementary Fig. 10 AC-HADDF-STEM images of (a) PyCoPc/GF, (b) CoPc/GF.

Comment 5.

A detailed process for the stability test conducted in the H-type cell should be provided. The conversion rate of dimethylamine (DMA) must be calculated, and LSV curves for DMF electrosynthesis in the presence and absence of KI should be supplied to verify the mediating role of I^-.

Response: Thanks for the comment.

1. We have provided a detailed description of the stability test procedure in the Supporting Information. The stability was conducted at a constant current density of 50 mA cm^{-2}, with periodic replacement of both the anolyte and catholyte every 10 h, accompanied by quantitative analysis of the reaction products. To mitigate interference from generated products on the electrodes and membrane, the electrode was rinsed with methanol and fresh electrolyte, and the membrane was rinsed with electrolyte during each electrolyte exchange.

Changes made in Revised Supporting Information:

Page 3, lines 10-14.

Stability testing in an H-type cell was conducted at a constant current density of 50 mA cm^{-2}, with periodic replacement of both the anolyte and catholyte every 10 h accompanied by quantitative analysis of the reaction products. During each electrolyte exchange, the electrode was rinsed alternately with methanol and fresh electrolyte, while the membrane surface was rinsed with electrolyte to remove surface-adsorbed products.

2. We calculated the DMA conversion rate during the 150 h stability test process, and the average value was 35% (Supplementary Fig. 28).

Changes made in Revised Supporting Information

Page 36.

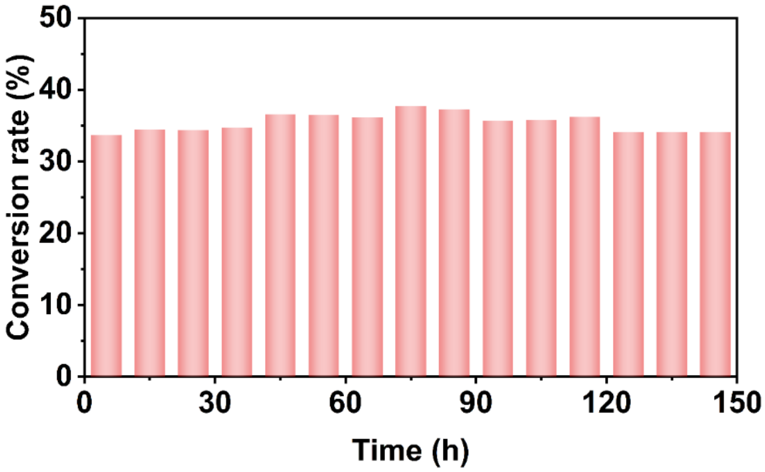

Supplementary Fig. 28 DMA conversion rate of PyCoPc/GF during the stability test at 50 mA cm^{-2}.

Changes made in Revised Manuscript:

Page 11, lines 26-29.

Furthermore, the long-term stability test for 150 h demonstrates that PyCoPc/GF exhibits consistent catalytic activity, capable of well maintaining the high FE_{DMF} (~90%), DMF productivity (839 μmol h^{-1} cm^{-2}) and a DMA conversion rate of ~35% (Fig. 3e and Supplementary Figs. 28-33).

3. We have supplemented the manuscript with LSV curves obtained under varying KI concentrations (Supplementary Fig. 19) and for different catalysts (Supplementary Fig. 25). As shown in Supplementary Fig. 19, the addition of KI significantly increased the current and induced a negative shift in the onset potential, from approximately 0.42 V without KI to about 0.30 V at a KI concentration of 100 mM.

Changes made in Revised Manuscript:

Page 9, lines 24-27.

Linear sweep voltammetry (LSV) was initially employed to evaluate the effect of KI on the catalytic reaction. As shown in Supplementary Fig. 19, the addition of KI significantly increased the current and induced a negative shift in the onset potential, from approximately 0.42 V without KI to about 0.30 V at a KI concentration of 100 mM.

Changes made in Revised Supporting Information:

Page 27.

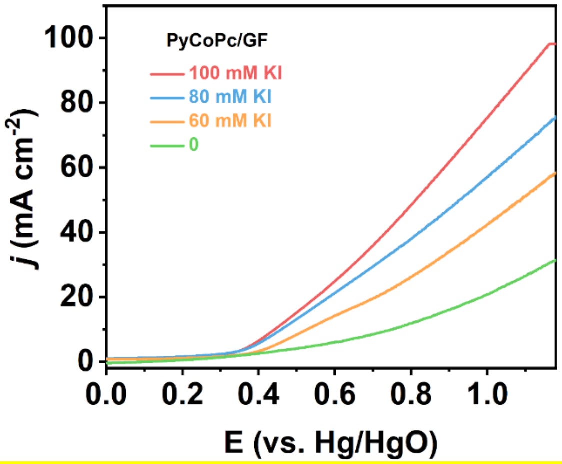

Supplementary Fig. 19 LSV cures of PyCoPc/GF with different concentrations of KI.

Comment 6.

The cobalt content in the PyCoPc/GF catalyst should be quantified.

Response: Thanks for the comment. We used ICP-OES to determine the Co content of GF-based electrode. As shown in Supplementary Table 2, the Co content of PyCoPc/GF (0.0046%) is higher than that in CoPc/GF (0.0033%), due to the pyrrolidone group substitution facilitating anchoring on the GF substrate.

Changes made in Revised Manuscript:

Page 8, lines 17-19.

The Co content of PyCoPc/GF (0.0046%) is higher than that in CoPc/GF (0.0033%) (Supplementary Table 2).

Changes made in Revised Supporting Information:

Page 76.

Supplementary Table 2 Co contents in GF-based electrodes as determined by ICP-OES.

Samples	Co contents
CoPc/GF	0.0033%
PyCoPc/GF	0.0046%

Comment 7.

The effect of I^- on the formation of the $*CH_2O$ intermediate should be clarified through

in situ ATR-IR characterization, including control experiments without KI over PyCoPc/GF.

Response: Thanks for the comment. We performed *in situ* ATR-IR tests with and without KI additives. As shown in Supplementary Fig. 36, the KI-containing system significantly enhances $*CH_2O$ intermediate generation and promotes its rapid conversion to DMF via I_2-mediated electro-oxidative decomposition of TMDM.

Changes made in Revised Manuscript:

Page 13, lines 9-16.

As shown in Supplementary Fig. 36, the peak at 1662 cm^{-1} corresponds to $*CH_2O$ derived from methanol partial oxidation, which subsequently reacts with DMA in 0.7 M K_2CO_3 to form TMDM rapidly. Compared to the KI-free system, KI addition significantly enhances $*CH_2O$ intermediate generation and facilitates rapid DMF production via I_2-mediated electro-oxidative decomposition of TMDM, as evidenced by the characteristic DMF peak at 1623 cm^{-1}.[6, 8]

Changes made in Revised Supporting Information:

Page 44.

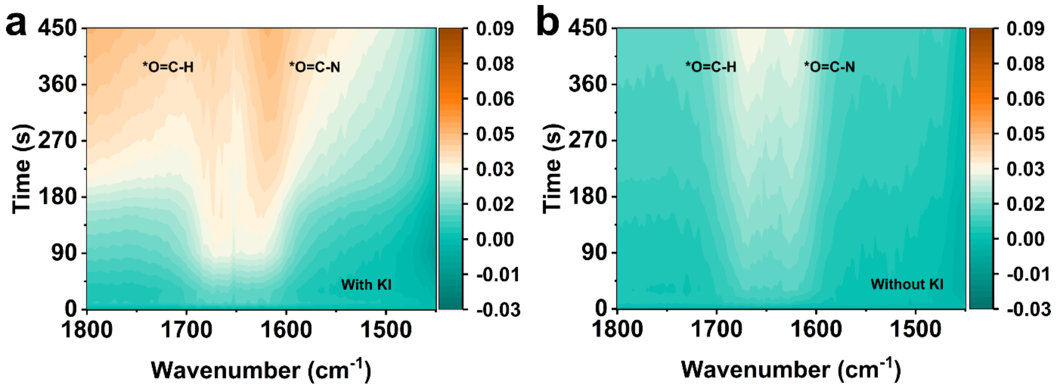

Supplementary Fig. 36 *In situ* ATR-IR spectrum for the DMF electrosynthesis over PyCoPc/GF in 0.7 M K_2CO_3 (a) with 100 mM KI, (b) without KI.

<u>Comment 8.</u>

A detailed explanation for the peak shift observed in the quasi-in situ NMR spectrum (Figure 4f) is required.

Response: Thanks for the comment. To further elucidate the chemical shift evolution of

key intermediates in *quasi-in situ* NMR, we repeated the measurements in $CDCl_3$ with enhanced temporal resolution. As shown in Supplementary Fig. 42 and Fig. 4c, I_2 rapidly reacts with TMDM to generate the highly reactive iodinated intermediate, 1-iodo-*N,N,N′,N′*-terametheylmethanediamine. As the reaction progresses, the integrated intensity of the C–H proton signal for this intermediate gradually increases and its chemical shift moves downfield, eventually reaching a steady state. This downfield shift is attributed to reduced electron density around the C–H bond due to strong electronic interactions with I_2 and the solvent, resulting in deshielding. Notably, the iminium cation exhibits a distinct upfield shift to 7.49 ppm in 0.7 M K_2CO_3 (Fig. 4d), compared to 8.53 ppm in $CDCl_3$. This contrast further illustrates the complex influence of the solvent environment on electronic structure and shielding effects.

Changes made in Revised Manuscript:

Page 12, Fig. 4c.

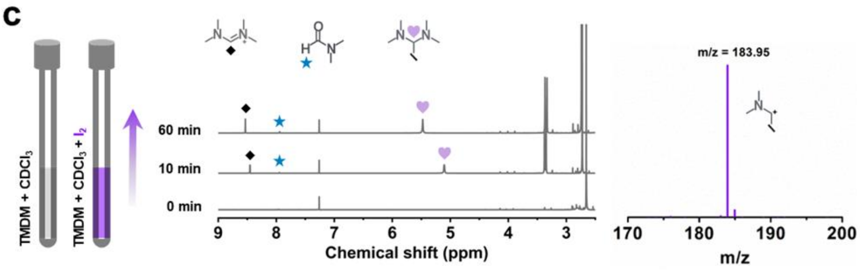

(c) formation and verification of iododiamine and iminium cation intermediates by *quasi-in situ* NMR and mass spectrometry;

Changes made in Revised Supporting Information:

Page 50.

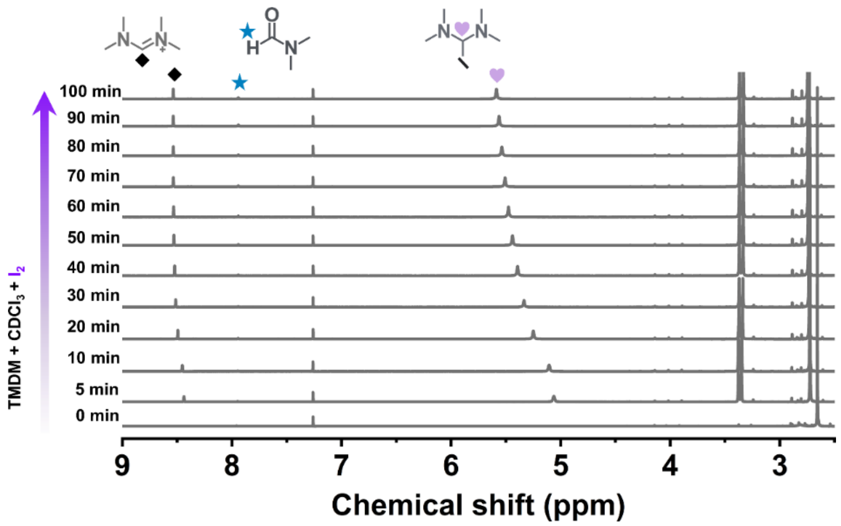

Supplementary Fig. 42 *Quasi-in situ* ^{1}H NMR spectra of TMDM in $CDCl_3$ with 10 mg I_2.

As shown in Supplementary Fig. 42 and Fig. 4c, I_2 rapidly reacts with TMDM to generate the highly reactive iodinated intermediate, 1-iodo-*N,N,N′,N′*-terametheylmethanediamine. As the reaction progresses, the integrated intensity of the C–H proton signal for this intermediate gradually increases and its chemical shift moves downfield, eventually reaching a steady state. This downfield shift is attributed to reduced electron density around the C–H bond due to strong electronic interactions with I_2 and the solvent, resulting in deshielding. Notably, the iminium cation exhibits a distinct upfield shift to 7.49 ppm in 0.7 M K_2CO_3 (Fig. 4d), compared to 8.53 ppm in $CDCl_3$ (Fig. 4c) This contrast further illustrates the complex influence of the solvent environment on electronic structure and shielding effects.

Comment 9.

The influence of the reaction solution composition (line 346) on the reaction mechanism should be elucidated. The bipolar membrane employed in the flow system may enhance catalytic activity by facilitating OH^- transport; thus, additional studies to investigate the effect of pH on DMF electrosynthesis activity are recommended. Furthermore, in contrast to CO_2-saturated catholyte, the introduction of CO_2 into an alkaline solution may fail to maintain the stability of the cathodic environment—this phenomenon's implications on overall reaction performance should be assessed.

Response: Thanks for the comment.

1. The electrolyte composition described in line 346 pertains to the cathode-side electrocatalytic CO_2 reduction. To more clearly illustrate the gas diffusion electrode (GDE), we have supplemented a schematic diagram of the GDE (Supplementary Fig. 61). Fabrication of the GDE established a stable CO_2-catalyst-electrolyte three-phase interface, significantly enhancing CO_2 mass transport efficiency. Crucially, since CO_2 is directly supplied to the electrode rather than dissolved in the bulk electrolyte, the original 0.5 M $KHCO_3$ in the H-type cell was replaced with 1 M KOH. This substitution reduces the cathodic reaction overpotential, thereby lowering the full-cell voltage and improving the performance of both electrodes. During flow cell testing, optimized control of CO_2 flow rate and electrolyte velocity maintained a robust three-phase interface, effectively mitigating pH fluctuations caused by CO_2 dissolution in the alkaline electrolyte. Additionally, the limited dissolution of CO_2 in the electrolyte volume (60 mL per cycle) had a negligible impact on pH. Post-reaction measurements confirmed that the catholyte pH remained stable at approximately 14.

Changes made in Revised Supporting Information:

Page 65.

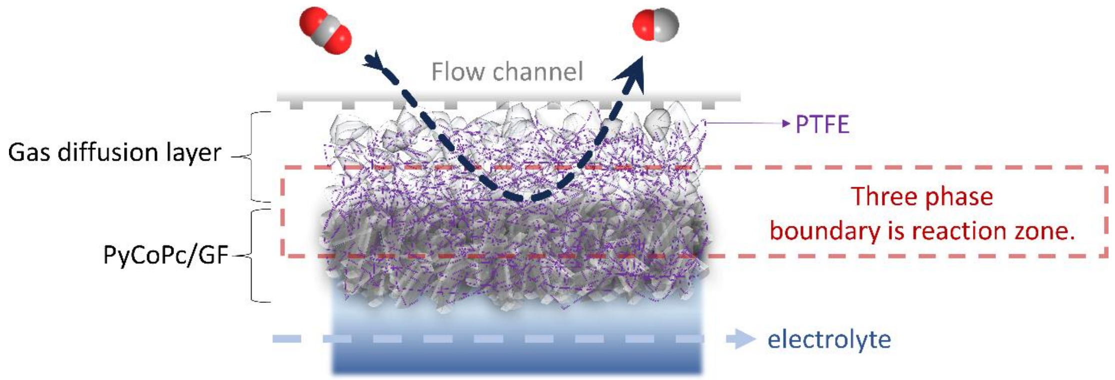

Supplementary Fig. 61 Schematic of the GDE configuration.

Fabrication of a gas diffusion electrode (GDE) established a stable CO_2-catalyst-electrolyte three-phase interface, significantly enhancing CO_2 mass transport efficiency. Direct CO_2 supply to the electrode facilitated replacement of the catholyte (0.5 M $KHCO_3$) in the H-type cell with 1 M KOH, avoiding bulk dissolution. This substitution reduces the cathodic overpotential, thereby lowering the full-cell voltage and improving the performance of both electrodes.

Changes made in Revised Manuscript:

Page 18, line 20.

…by constructing the gas diffusion electrode (GDE) with distinctive gas-liquid-solid triple-phase interfaces (Supplementary Fig. 61)…

Page 18, lines 18-19.

…substituting the catholyte from neutral 0.5 M $KHCO_3$ to 1 M KOH effectively reduces overall cell voltage while simultaneously improving the performance of both cathodic and anodic reactions.

2. The bipolar membrane was implemented to effectively suppress organic crossover from the anolyte to the catholyte, preventing interference with electrode reactions in both compartments, while simultaneously enhancing catalytic activity by providing additional OH^- to the anode through electrochemically dissociating water at its intermediate interfacial layer (*Angew. Chem. Int. Ed.* 2026, 65, e17470; *Appl. Energy* 2025, 394, 126176). Alkaline conditions promote DMF formation, as describe by the reaction mechanism (Fig. 5a). In representative H-type cell testing, the anolyte contained 15 mL of 0.7 M K_2CO_3, 12 mL MeOH, and 3 mL DMA. The baseline 0.7 M K_2CO_3 solution exhibits a pH of ~12, while the addition of 3 mL DMA elevates this value to ~14. Consequently, assessing pH effects would introduce significant experimental complexity and additional variables into the reaction system. Based on literature and prior research (*Green Chem.* 2023, 25, 5936-5944; *Appl. Catal. B: Environ.* 2026, 382, 125989; *Nat. Commun.* 2025, 16, 3564), the 0.7 M K_2CO_3 electrolyte promotes selective electro-oxidation of methanol to generate the $*CH_2O$ intermediate, while a 4:1 MeOH-to-DMA ratio enhances DMF generation.

Changes made in Revised Manuscript:

Page 10, line 14 to Page 11, line 7.

As shown in Supplementary Fig. 23, all three membranes exhibited comparable catalytic activity, with the bipolar membrane (BM) achieving a marginally higher FE_{DMF} (91%). BM was selected for subsequent testing due to its dual functionality of providing additional OH^- to the anode through electrochemical water dissociation at the interfacial layer, thereby promoting DMF electrosynthesis, while effectively suppressing organic

crossover from the anolyte to the catholyte during prolonged operation to prevent interference with electrode reactions in both compartments.[31, 32]

Page 24, lines 15-21.

[31] W. Zhu, Q.-C. Chen, Y. Chen, J. E. Huang, G. Su, H. Liu, W. Ni, Y. Chen, J. Yu, B. Peng, J. Li, S. Lee, S. Hao, Y. Shen, H. Ze, B. Zhou, X.-Y. Li, Y. Ji, S. Yang, C. Tian, Y. Liang, K. Xie, E. H. Sargent, High-concentration alcohol generation in bipolar membrane CO electrolyzer. *Angew. Chem. Int. Ed.* **2026**, *65*, e17470.

[32] F. Meng, D. Cheng, M. Lin, K. Zuo, Z. B. Zhang, Review on crossover minimization and catalytic layer-promoted water dissociation in bipolar membranes. *Appl. Energy* **2025**, *394*, 126176.

Changes made in Revised Supporting Information:

Page 4, lines 6-9.

Based on literature and prior research, the 0.7 M K_2CO_3 electrolyte promotes selective electro-oxidation of methanol to generate the $*CH_2O$ intermediate,[1-3] while a 4:1 MeOH-to-DMA ratio enhances DMF generation.

Page 84, lines 2-11.

[1] Y. He, X. Xiao, X. Li, D.-D. Ma, Q.-L. Zhu, Q. X., Unlocking high-efficiency electrosynthesis of *N,N*-Dimethylformamide through the synergy of three-dimensional ordered macroporous superstructure and implanted nano-tentacles. *Appl. Catal. B-Environ.* **2026**, *382*, 125989.

[2] Y. He, D.-D. Ma, K. Ma, X. Li, L. Han, X.-T. Wu, Q.-L. Zhu, Electrocatalytic N–C–N coupling over a hierarchically ordered open single-atom superstructure toward organonitrogen synthesis. *Nat. Commun.* **2025**, *16*, 3564.

[3] M. Jin, A.-Z. Li, Y. Wang, J. Li, H. Zhou, B.-J. Li, H. Duan, Electrosynthesis of *N,N*-dimethylformamide from market-surplus trimethylamine coupled with hydrogen production. *Green Chem.* **2023**, *25*, 5936-5944.

Referee Report
Title: Redox-Mediated Domino Electrosynthesis of *N,N*-Dimethylformamide with Industrial-Relevant Productivity
Manuscript: NCOMMS-25-83405

In the related manuscript, the authors propose a molecularly modified electrode (PyCoPc/GF) for selective methanol oxidation, coupled with a CO_2 reduction process in a bipolar membrane flow cell. The idea of using molecular Co–phthalocyanine sites for oxidation–reduction paired electrosynthesis is scientifically appealing. However, several key aspects of the electrochemical characterization, mechanistic elucidation, and catalyst stability are insufficiently supported by data. Therefore, I do not recommend acceptance in its current form, but the work could be reconsidered after major revision and substantial experimental clarification.
Below are specific, constructive points to improve the manuscript, listed point-by-point

1. No cyclic voltammograms (CVs) or linear sweep voltammograms (LSVs) are shown for the anode in methanol oxidation conditions. The SI mentions such measurements, but the only voltammograms displayed correspond to CO_2 reduction. Please include LSV/CV data for PyCoPc/GF, CoPc/GF, and GF electrodes in the actual anolyte (MeOH:DMA/K_2CO_3 ± KI). These are crucial to demonstrate intrinsic activity, onset potentials, and kinetic differences.

2. In the SI, it is mentioned that Pt counter electrode was used in a three-electrode compartment cell. even in iodide-containing media and under strongly cathodic potentials. Platinum can undergo cathodic corrosion, forming soluble species that may redeposit on the anode, compromising the catalytic interpretation. Could authors, please comment on that?

3. The claim that "the architecture maintains structural integrity" is not supported beyond post-run SEM/EDX and XRD. As this is an important discussion/interpretation of the manuscript, more details post electrolysis should be provided.

4. The proposed mechanism involves methanol oxidation to formaldehyde, followed by iodide-mediated transformation of TMDM to iminium and finally to DMF. However, the initial methanol oxidation step on PyCoPc/GF is not directly monitored. Please, provide formaldehyde quantification (e.g., by Nash test or GC/HPLC) during electrolysis to verify this step, and include control experiments without iodide to distinguish the intrinsic activity of PyCoPc from redox mediation by I□/I^-.

5. The manuscript attributes high performance to "increased exposure of active sites," but no electrochemically active surface area (ECSA) or Co-site loading is reported. Please, provide double-layer capacitance (Cdl) or

other ECSA measurements, Co loading by ICP, and ideally a turnover frequency (TOF) normalized to active Co sites.

6. The mediator plays a central role, but its long-term stability is not examined. Please analyze iodide/iodine concentrations before and after extended electrolysis (e.g., by UV–vis or iodometric titration) to confirm that the mediator is not depleted or transformed irreversibly.

7. Recent literature (Broersen et al. *J. Am. Chem. Soc. 2025, 147, 12, 10298–10308*), showed the electrochemical N-formylation of amines via methanol oxidation occurs via iso(cyanides) intermediate. Please provide details about parallel reaction.
